# Investigation of the Effect of 2,3-Dihydrobenzoic Acid Acid (2,3-DHBA) on the Lipid Profiles of MCF-7 and MDA-MB-231 Human Breast Cancer Cells via an Untargeted Lipidomic Approach

**DOI:** 10.3390/biom15091341

**Published:** 2025-09-19

**Authors:** Büşra Daş, Serap Şahin

**Affiliations:** 1 Department of Biochemistry, Faculty of Pharmacy, Sivas Cumhuriyet University, 58140 Sivas, Türkiye; bsrkaradag@hotmail.com; 2 Department of Biochemistry, Faculty of Pharmacy, Afyonkarahisar Health Sciences University, 03030 Afyonkarahisar, Türkiye

**Keywords:** 2,3-dihydroxybenzoic acid, MCF-7, MDA-MB-231, MTT, lipidomics, cytotoxic activity

## Abstract

Breast cancer (BC) is a primary cause of cancer-related mortality in women, making the development of novel therapeutic strategies essential. Altered lipid metabolism is a recognized hallmark of cancer, presenting a key therapeutic vulnerability. This study investigated the cytotoxic effects of the natural phenolic compound 2,3-DHBA on MCF-7 (luminal A) and MDA-MB-231 (triple-negative) human breast cancer cells and characterized the associated changes in their lipid profiles via an untargeted lipidomic approach. The in vitro cytotoxicity of 2,3-DHBA was assessed using the MTT assay at 24, 48, and 72 h against both cancer cell lines and non-cancerous L-929 fibroblasts. Following treatment with the 48-h IC_50_ concentrations (8.61 mM for MCF-7, 5.84 mM for MDA-MB-231), total lipids were extracted and analyzed. The results showed that 2,3-DHBA exerted potent time- and dose-dependent cytotoxic effects against both BC cell lines, with significantly higher selectivity for cancer cells over healthy fibroblasts. The more aggressive MDA-MB-231 line exhibited greater sensitivity. The lipidomic analysis revealed that 2,3-DHBA induced profound cell-specific alterations across all major lipid classes, including fatty acids, glycerolipids (GLs), glycerophospholipids (GPs), and sphingolipids (SPs). These changes suggest a multi-pronged mechanism involving the disruption of membrane integrity through GP remodeling, the attenuation of survival signaling via the GL network, and a critical shift in the sphingolipid rheostat towards pro-apoptotic ceramide accumulation. This study establishes a direct link between the cytotoxic activity of 2,3-DHBA and its ability to comprehensively reprogram the cancer cell lipidome, highlighting its potential as a sophisticated metabolic modulator for breast cancer therapy.

## 1. Introduction

The discovery of salicin and the subsequent synthesis of aspirin spurred the growth of a pharmaceutical industry focused on isolating and identifying pharmacologically active compounds from plants; this pursuit extended to the isolation of such drugs from herbal preparations [1,2]. The recognition that many foods also contain pharmacologically active compounds and that nutrients can thereby contribute to protection against lifestyle-related and age-associated diseases remains a significant area of contemporary research.

Phenolic compounds, representing a major group of water-soluble antioxidants, are aromatic secondary metabolites found abundantly in various plant parts, including fruits, vegetables, seeds, flowers, leaves, branches, and stems [3]. These compounds are broadly classified into flavonoids and phenolic acids. Both groups are characterized by the presence of one or more hydroxyl groups attached to a benzene ring; their biological activities differ based on factors such as the number and binding position of these hydroxyl groups [4,5]. Phenolic compounds significantly influence plant color, odor, and taste, and they protect plants from external stressors [6]. Many phenolic acids isolated from plants, including gallic, chlorogenic, caffeic, ellagic, and ferulic acids, are pharmacologically active and have demonstrated antimutagenic, antioxidant, anticarcinogenic, antimicrobial, antiviral, and antitumor effects [7,8,9]. These naturally occurring hydroxylated compounds, derived from benzoic and cinnamic acids, are classified into two main groups: hydroxybenzoic acids (benzoic, salicylic, vanillic, gallic acids) and hydroxycinnamic acids (p-coumaric, sinapic, caffeic, ferulic acids) [10]. 2,3-dihydroxybenzoic acid (2,3-DHBA) is among the dihydroxybenzoic acid (DHBA) compounds related to salicylic acid [11,12]. It is found in several fruit species in South and Southeast Asia, such as batoko plum, avocado, and blueberry, and its main dietary source includes Aspergillus-fermented soy products that are popular in Japan [13,14,15]. 2,3-DHBA is a naturally occurring iron carrier in plants, exhibiting a greater binding constant to iron than transferrin and lactoferrin. Consequently, 2,3-DHBA has been investigated as an effective oral iron-chelating agent and potential iron stabilizer in patients with β-thalassemia [16,17,18].

Given their diverse and crucial biological roles, the characterization of lipids is highly important for health research [19]. Lipidomics is a field dedicated to the comprehensive identification, quantification, and characterization of lipid species (the lipidome) and the study of their biological roles, including their metabolism and regulation by associated proteins and genes [20,21]. There are three (targeted, untargeted, and focused) different types of lipidomic methods [22,23]. Untargeted lipidomic methods record comprehensive mass spectrometry (MS) profile data from lipid extracts of biological samples to directly investigate changes in lipid profiles or identify abnormal lipid metabolites resulting from biological degradation or stimulation [24]. Lipids have been implicated in the progression of various cancers (e.g., prostate, breast, glioblastoma) and in early carcinogenesis [25,26,27,28,29]. Furthermore, alterations in lipid profiles are strongly correlated with diseases such as cardiovascular diseases, Parkinson’s disease, schizophrenia, atherosclerosis, Alzheimer’s disease, diabetes, multiple sclerosis, and polycystic ovary syndrome [30,31].

According to the WHO 2022 data, breast cancer represents the second most diagnosed cancer type, with approximately 2.3 million new cases worldwide, and it ranks fourth in cancer-related deaths (666,103 cases). In Türkiye, a total of 25,249 new cases and 7360 deaths occurred, ranking second in incidence and fifth in mortality [32]. For the treatment of cancer, the use of natural resources traditionally described as possible alternative and/or additional therapeutic agents may be preferred because the modern medicine that is available is expensive, toxic, and sometimes ineffective. The use of alternative therapies in cancer treatment is increasing rapidly in the USA, and it is stated that it corresponded to an industry of 1.2 billion dollars in 2023 [33]. It has been reported that 80% of cancer patients use alternative or supportive medicine, and at least one-third of them use alternative drugs in combination with chemotherapy treatment [34]. Epidemiological studies indicated that diets high in plant-derived polyphenols are linked to a reduced risk of developing chronic diseases, including neurodegenerative diseases, inflammation, cancer, cardiovascular diseases, type 2 diabetes, and obesity [35].

Despite the established roles of various phenolic acids and the known iron-chelating properties of 2,3-DHBA, comprehensive information regarding its specific therapeutic mechanisms against cancer, particularly breast cancer, and its impact on cellular lipid metabolism is limited. Therefore, we aimed to, firstly, investigate the in vitro cytotoxic activity of 2,3-DHBA against human breast cancer cell lines (MCF-7 and MDA-MB-231). We next performed an untargeted lipidomic analysis by characterizing changes in the lipid profiles of these breast cancer cells following incubation with 2,3-DHBA. This approach, which has not been previously applied in this specific context, allowed us to gain novel insights into the lipid-mediated cellular responses to 2,3-DHBA in breast cancer.

## 2. Materials and Methods

### 2.1. Cells and Reagents

Human breast cancer cell lines MCF-7 (ATCC^®^ HTB-22™) and MDA-MB-231 (ATCC^®^ HTB-26™), fetal bovine serum (FBS), and antibiotics (100 Units/mL penicillin and 100 μg/mL streptomycin) were (American Type Culture Collection (ATCC), Manassas, VA, USA). L-929 mouse fibroblast cells (NCTC clone 929, ECACC 85011425) were (European Collection of Authenticated Cell Cultures (ECACC, Salisbury, UK). Dulbecco’s Modified Eagle’s Medium (DMEM, D6429) (Merck, Darmstadt, Germany), Roswell Park Memorial Institute 1640 medium (RPMI-1640) (Merck, Darmstadt, Germany), MTT [3-(4,5-dimethylthiazol-2-yl)-2,5-diphenyltetrazolium bromide; M-2128]) (Merck, Darmstadt, Germany), trypsin-EDTA solution (T-3924) (Merck, Darmstadt, Germany), 2,3-dihydroxybenzoic acid (catalog number, 126209), chloroform, methanol, ammonium molybdate, hydrogen peroxide, dimethyl sulfoxide, sulfuric acid, and ascorbic acid were purchased from (Merck, Darmstadt, Germany). The lipid standards 1-heptadecanoyl-2-(9Z-tetradecenoyl)-sn-glycero-3-phosphocholine and 1-stearoyl-2-stearoyl(d35)-sn-glycero-3-phosphocholine were purchased from Avanti^®^ Lipids (Avanti^®^ Lipids, Birmingham, AL, USA).

### 2.2. Growth Condition of the Cells

Human breast cancer cells MCF-7 (ATCC^®^ HTB-22^TM^) and MDA-MB-231 (ATCC^®^ HTB-26^TM^) were cultured in DMEM medium, while L-929 cells were maintained in RPMI-1640 medium, supplemented with 10% (*v*/*v*) fetal bovine serum (FBS) and 1% antibiotic solution containing 100 Units/mL penicillin and 100 μg/mL streptomycin. Cells were incubated at 37 °C in a humidified atmosphere containing 5% CO_2_. All cell lines were subcultured upon reaching 70–80% confluence.

### 2.3. In Vitro Cytotoxic Activity Assay of 2,3-DHBA

The in vitro cytotoxic activity of 2,3-DHBA was evaluated using the MTT assay [36]. Briefly, cells were seeded in 96-well plates at a density of 1 × 10^5^ cells/mL (100 µL) in appropriate media and incubated for 24 h at 37 °C in a humidified atmosphere containing 5% CO_2_ to allow cell attachment. Following attachment, cells were treated with various concentrations (0.75–20 mM) of 2,3-DHBA and incubated for 24, 48, and 72 h. The compound was dissolved in DMSO and diluted in complete culture medium, with the same volume of DMSO added to control wells (the final DMSO concentration did not exceed 1% in any well). At the end of each incubation period, 10 μL of MTT solution (5 mg/mL in PBS, pH 7.2) was added to each well. After 2 h of incubation with MTT at 37 °C, the medium was carefully removed, and 100 μL of DMSO was added to dissolve the formazan crystals. Following 15 min of gentle shaking at room temperature, absorbance values were measured using an ELISA plate reader (BioTek Epoch, Santa Clara, CA, USA) at 570 nm. The IC_50_ values (concentration required to inhibit 50% of cell viability) of 2,3-DHBA at 24, 48, and 72 h were calculated using the GraphPad Prism 8 software (GraphPad Software, San Diego, CA, USA).

### 2.4. Lipidomics Analysis

#### 2.4.1. Lipid Isolation with the Bligh–Dyer Method

MCF-7 and MDA-MB-231 breast cancer cells were cultured in 25 cm^2^ flasks at a density of 1 × 10^5^ cells/mL. The IC_50_ concentrations of 2,3-DHBA determined by the MTT assay were applied to cells as follows: 8.61 mM for MCF-7 and 5.84 mM for MDA-MB-231 breast cancer cells for 48 h. Lipid extraction was performed after 48 h using the Bligh–Dyer method [37] from treated cells (*n* = 6). Cells incubated with DMSO served as controls (*n* = 6). Cells were washed with 1×PBS, scraped with a methanol–water mixture, and transferred to tubes. The extraction procedure involved adding methanol (2.5 mL), chloroform (1.25 mL), water (1.5 mL), and lipid standards (100 μL) to each tube. Tubes were vortexed for 30 s, incubated on ice for 10 min, and centrifuged at 300× *g* for 5 min. The bottom chloroform layer was carefully transferred to a new tube. The extraction was repeated with an additional 1.5 mL of chloroform, and the chloroform layers were combined. The pooled chloroform extracts were dried under nitrogen gas, reconstituted in 50–75 μL of methanol–chloroform (3:1, *v*/*v*), and stored at −80 °C until LC-MS/MS analysis.

#### 2.4.2. Lipid Phosphorus Assay

The lipid phosphorus content was quantified using the phosphorus assay [38]. Lipid extracts (8 μL) and sulfuric acid (400 μL) were combined in glass tubes and incubated at 200 °C for 1 h. Hydrogen peroxide (30%, 100 μL) was added to each tube while vortexing, followed by an additional incubation at 200 °C for 1.5 h. Molybdate reagent (4.6 mL; prepared by dissolving 1.1 g of ammonium molybdate tetrahydrate in 12.5 mL of sulfuric acid and diluting to 500 mL with ddH_2_O) was added and mixed by vortexing. Subsequently, ascorbic acid solution (15%, 100 μL) was added with vortexing. The tubes were heated for 7–10 min at 100 °C, and a 150 μL aliquot was transferred to measure the absorbance at 830 nm using a microplate reader (BioTek Epoch, Santa Clara, CA, USA).

#### 2.4.3. Determination of Lipid Types via Electrospray Ionization Mass Spectrometry (ESI-MS)

Lipid extract samples were prepared at a concentration of 500 pmol/μL via reconstitution in chloroform–methanol (2:1, *v*/*v*). ESI-MS analysis of lipid extracts was performed using an Agilent 6530 Accurate-Mass Q-TOF LC/MS mass spectrometer (Agilent Technologies, Santa Clara, CA, USA) as previously described [37,38,39]. The instrument operated with a nitrogen drying gas flow rate of 8 L/min at 350 °C and a nebulizer pressure of 30 psi. Mass spectra were acquired over a scanning range of 200–1200 *m*/*z* using 5 μL sample injections in both positive ion mode (PIM) and negative ion mode (NIM) for 5 min. The mobile phase consisted of acetonitrile–methanol–water (2:3:1) containing 0.1% ammonium formate. Qualitative identification of individual phospholipid molecular species was based on their calculated theoretical monoisotopic mass values and subsequent MS/MS analysis, with intensities normalized to either the total ion count (TIC) or the most abundant phospholipid, as previously described [40]. Tentative identification was assigned based on *m*/*z* ratios and comparison with the LIPID MAPS database (http://www.lipidmaps.org; accessed on 24 June 2025) [41].

MS^nth^ fragmentation analysis was performed using the same Agilent 6530 Accurate-Mass Q-TOF LC/MS system equipped with an ESI source via direct injection from the HPLC system. The nitrogen drying gas flow rate was maintained at 8.0 L/min at 350 °C, with the ion source and ion optic parameters optimized for the positive molecular ion of interest. The initial identification was typically based on the characteristic loss of the parent head group, followed by subsequent analysis of the resulting lysophospholipid fragment. When neutral loss scanning could not confirm the species identity, tentative identification was assigned based on *m*/*z* values and comparison with the LIPID MAPS database (http://www.lipidmaps.org; accessed on 24 June 2025) [41].

#### 2.4.4. Multivariate Statistical Analysis of Lipids

Multivariate analyses, including principal component analysis (PCA) and partial least squares discriminant analysis (PLS-DA), were performed using MetaboAnalyst 4.0 (http://www.metaboanalyst.ca/; accessed on 21 June 2025) [42]. Automatic peak detection and spectrum deconvolution were conducted with a peak width set to 0.5. Data were normalized to the sum and z-score scaled. No outliers were removed from the analysis. Significantly altered lipids were identified using volcano plot analysis, which combines fold change and *t*-test results. Significance criteria for volcano plot analysis were established as a fold change threshold of ≥2.00 and *p* ≤ 0.05. Lipids meeting these criteria were further characterized using MS/MS analysis for structural confirmation. Following identification, the total ion count was used to normalize each parent lipid level, and changes in the relative abundance of phospholipid species compared to their respective controls were determined [43,44].

### 2.5. Statistical Analysis

All measurements in the cytotoxic activity experiments were taken in six replicates (*n* = 6), and the results are given with standard deviations (±SD). IC_50_ values were determined using the GraphPad Prism7 graph and statistics program (GraphPad Software, San Diego, CA, USA). Statistical analyses were performed using GraphPad Prism 8 (GraphPad Software, San Diego, CA, USA). All data were tested for normality. For normally distributed groups, one-way ANOVA was applied to compare more than two groups. When a significant difference between groups was detected, a post hoc Dunnett’s (for multiple comparisons against the control) test was applied. For non-normally distributed groups, the Kruskal–Wallis test was applied to compare more than two groups. When a significant difference between groups was detected, a post hoc Dunn’s multiple-comparison test was applied. Significance was taken as * *p* < 0.05, ** *p* < 0.005, ^#^ *p* < 0.0005, and ^##^ *p* < 0.0001.

## 3. Results

### 3.1. In Vitro Cytotoxic Activity of 2,3-DHBA Against MCF-7 and MDA-MB-231 Cells

**Figure 1:**
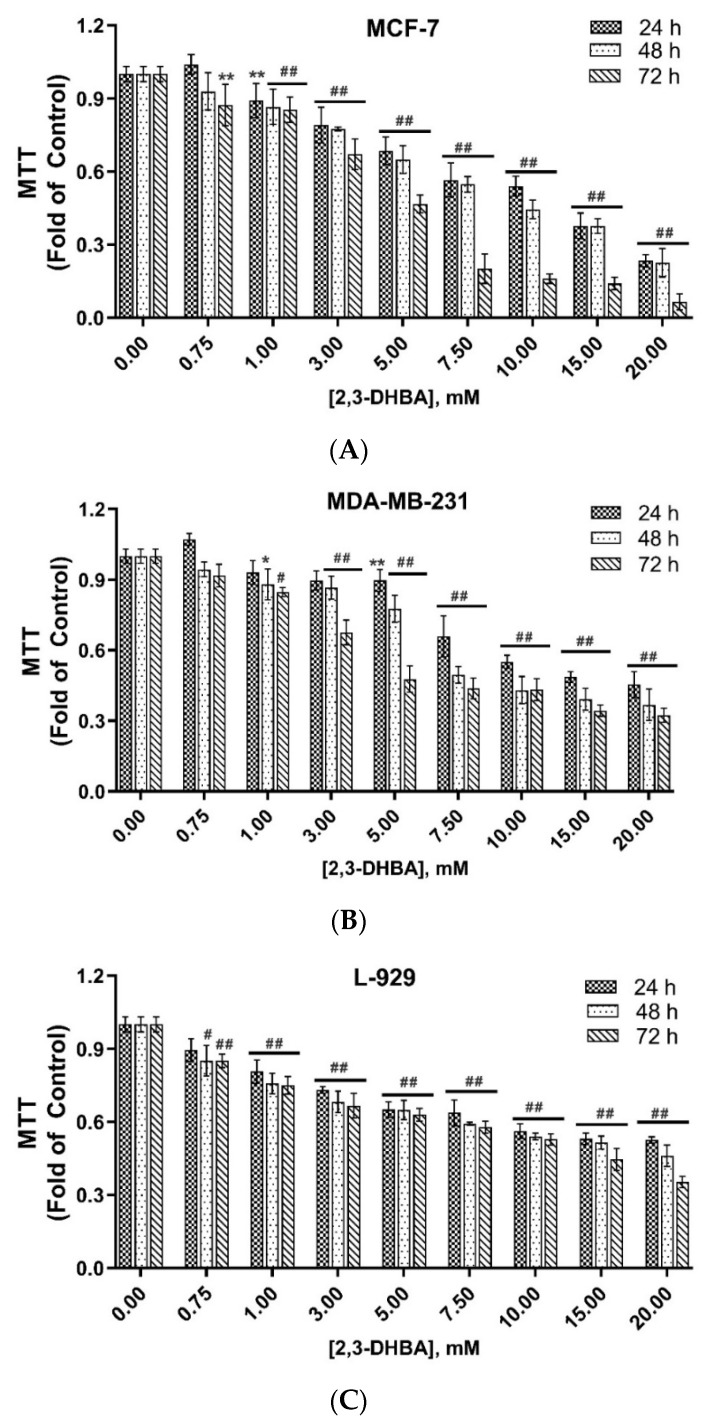
In vitro cytotoxic activity of 2,3-DHBA against (**A**) MCF-7 and (**B**) MDA-MB-231 human breast cancer cells and (**C**) L-929 healthy fibroblast cells. The results were given as ± SD (significance relative to control; * *p* < 0.05, ** *p* < 0.005, ^#^ *p* < 0.0005, ^##^ *p* < 0.0001) (*n* = 6).

The time- and concentration-dependent in vitro cytotoxic activities of 2,3-DHBA against MCF-7 and MDA-MB-231 breast cancer cell lines and L-929 healthy fibroblast cells were investigated using the MTT assay at 24 h, 48 h, and 72 h of incubation (Figure 1).

As shown in Figure 1A–C, a positive correlation was observed between higher 2,3-DHBA concentrations and increased in vitro cytotoxic activity. The IC_50_ values of 2,3-DHBA against MCF-7 and MDA-MB-231 breast cancer cells and L-929 healthy fibroblast cells were calculated for 24, 48, and 72 h (Table 1). The IC_50_ values of 2,3-DHBA for both breast cancer cells and healthy fibroblast cells decreased in a time-dependent manner. The IC_50_ concentrations of 2,3-DHBA against MDA-MB-231 breast cancer cells were consistently lower than those against MCF-7 cells. Additionally, the IC_50_ values for L-929 healthy fibroblast cells were higher than those for both MCF-7 and MDA-MB-231 breast cancer cells, demonstrating that 2,3-DHBA exhibits greater cytotoxicity against breast cancer cells and shows selective in vitro cytotoxic activity, preferentially targeting cancerous cells over healthy cells.

The selectivity index (SI) is an important parameter that indicates the selectivity of a given compound between normal and cancer cells. The expectation is that the compound should be less cytotoxic toward healthy cells compared to cancer cells. The SI was calculated by dividing the IC_50_ value of the compound on normal cells by the IC_50_ value of the compound on cancer cells. The SI values of 2,3-DHBA against MCF-7 and MDA-MB-231 breast cancer cells were calculated (Table 1). The results demonstrated that 2,3-DHBA was more selectively toxic toward MDA-MB-231 breast cancer cells, with SI values ranging from 1.90 to 3.54, compared to MCF-7 cells at 24, 48, and 72 h. Our results also indicated that the SI values of 2,3-DHBA against both MCF-7 and MDA-MB-231 cells increased with incubation time, suggesting enhanced selectivity with prolonged exposure.

### 3.2. Lipidomic Analysis

#### 3.2.1. Determination of Inorganic Phosphate

A standard curve of phosphorus solution was used to ensure concentration normalization of all samples obtained from lipid extraction using the Bligh–Dyer method from cancer cells. Phosphorus concentrations of all samples (with and without 2,3-DHBA treatment) were normalized to 500 pmol/μL using this standard curve, and ESI-MS analyses were subsequently performed using Q-TOF LC/MS.

#### 3.2.2. ESI-MS Analysis

ESI-MS data were collected in PIM and NIM using an Agilent 6530 Q-TOF LC/MS, with the specific parameters listed in the experimental section. Analysis of mass spectra obtained from MCF-7 control cells (for PIM, Figures S1 and S3; for NIM, Figures S5 and S7) and 2,3-DHBA-treated MCF-7 breast cancer cells (for PIM, Figures S2 and S4; for NIM, Figures S6 and S8), as well as from MDA-MB-231 control cells (for PIM, Figures S9 and S11; for NIM, Figures S13 and S15) and 2,3-DHBA-treated MDA-MB-231 breast cancer cells (for PIM, Figures S10 and S12; for NIM, Figures S14 and S16), revealed significant intensity variations in the *m*/*z* regions of 200–1200 and 600–900. These differences were observed in both PIM (Figures S1–S4 and S9–S12) and NIM (Figures S5–S8 and S13–S16).

#### 3.2.3. Multivariate Statistical Analysis for Lipids

**Figure 2:**
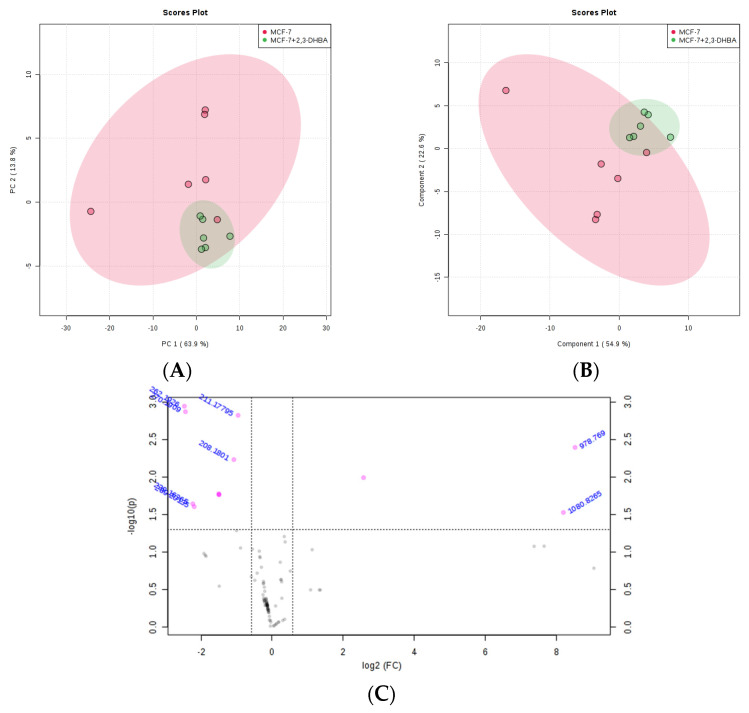
Effect of 2,3-DHBA exposure on the relative abundance of lipids in MCF-7 cells. MCF-7 cells were treated with either DMSO (control) or the IC_50_ concentration of 2,3-DHBA (8.61 mM) for PIM. Forty-eight hours after treatment, cells were collected, subjected to Bligh–Dyer extraction, and analyzed using ESI-MS. (**A**) PCA of lipids extracted from DMSO-treated cells (red) and 2,3-DHBA-treated cells (green). Each data point represents an individual replicate. The ovals represent 95% confidence intervals. (**B**) PLS-DA of lipids extracted from DMSO-treated cells (red) and 2,3-DHBA-treated cells (green). Each data point represents an individual replicate. The ovals represent 95% confidence intervals. (**C**) Volcano plot analysis comparing the log fold change of each *m*/*z* value (X-axis) to the significance value (Y-axis) for pairwise comparisons between the DMSO-treated (control) and 2,3-DHBA treatment groups. The identity of select *m*/*z* values is indicated next to each data point. Pink dots indicate significantly changed lipids, while gray dots indicate lipids that did not change significantly. All features presented are considered significant based on a fold change threshold of ≥2.00 and *t*-tests where *p* ≤ 0.05.

**Figure 3:**
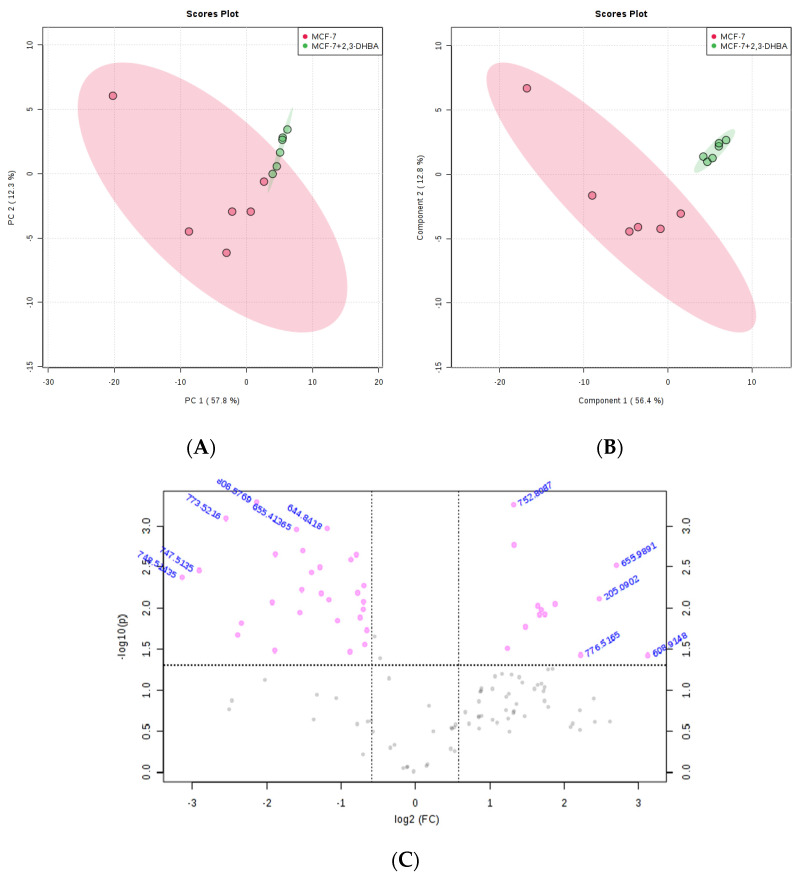
Effect of 2,3-DHBA exposure on the relative abundance of lipids in MCF-7 cells. MCF-7 cells were treated with either DMSO (control) or the IC_50_ concentration of 2,3-DHBA (8.61 mM) for NIM. Forty-eight hours after treatment, cells were collected, subjected to Bligh–Dyer extraction, and analyzed using ESI-MS. (**A**) PCA of lipids extracted from DMSO-treated cells (red) and 2,3-DHBA-treated cells (green). Each data point represents an individual replicate. The ovals represent 95% confidence intervals. (**B**) PLS-DA of lipids extracted from DMSO-treated cells (red) and 2,3-DHBA-treated cells (green). Each data point represents an individual replicate. The ovals represent 95% confidence intervals. (**C**) Volcano plot analysis comparing the log fold change of each *m*/*z* value (X-axis) to the significance value (Y-axis) for pairwise comparisons between the DMSO-treated (control) and 2,3-DHBA treatment groups. The identity of select *m*/*z* values is indicated next to each data point. Pink dots indicate significantly changed lipids, while gray dots indicate lipids that did not change significantly. All features presented are considered significant based on a fold change threshold of ≥2.00 and *t*-tests where *p* ≤ 0.05.

**Figure 4:**
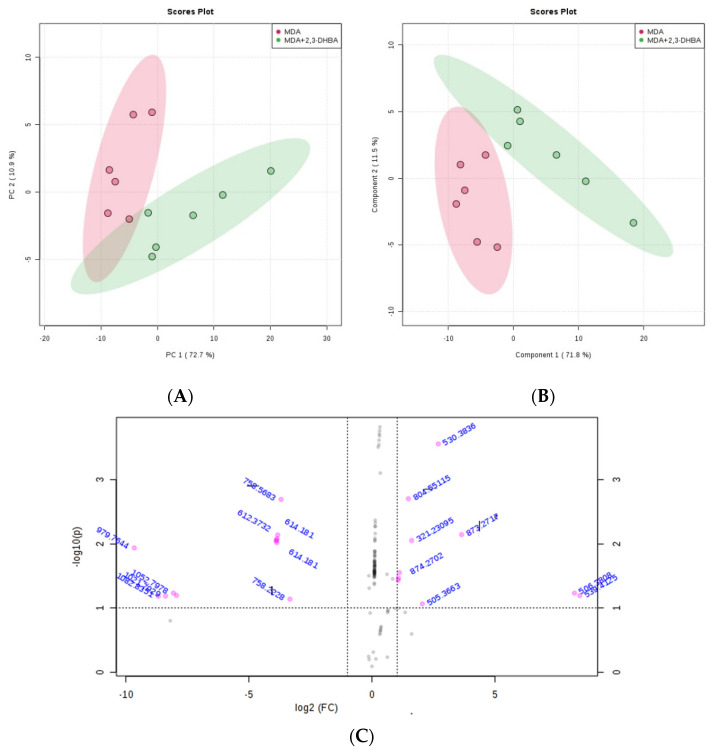
Effect of 2,3-DHBA exposure on the relative abundance of lipids in MDA-MB-231 cells. MDA-MB-231 cells were treated with either DMSO (control) or the IC_50_ concentration of 2,3-DHBA (5.48 mM) for PIM. Forty-eight hours after treatment, cells were collected, subjected to Bligh–Dyer extraction, and analyzed using ESI-MS. (**A**) PCA of lipids extracted from DMSO-treated cells (red) and 2,3-DHBA-treated cells (green). Each data point represents an individual replicate. The ovals represent 95% confidence intervals. (**B**) PLS-DA of lipids extracted from DMSO-treated cells (red) and 2,3-DHBA-treated cells (green). Each data point represents an individual replicate. The ovals represent 95% confidence intervals. (**C**) Volcano plot analysis comparing the log fold change of each *m*/*z* value (X-axis) to the significance value (Y-axis) for pairwise comparisons between DMSO-treated (control) and 2,3-DHBA treatment groups. The identity of select *m*/*z* values is indicated next to each data point. Pink dots indicate significantly changed lipids, while gray dots indicate lipids that did not change significantly. All features presented are considered significant based on a fold change threshold of ≥2.00 and *t*-tests where *p* ≤ 0.05.

**Figure 5:**
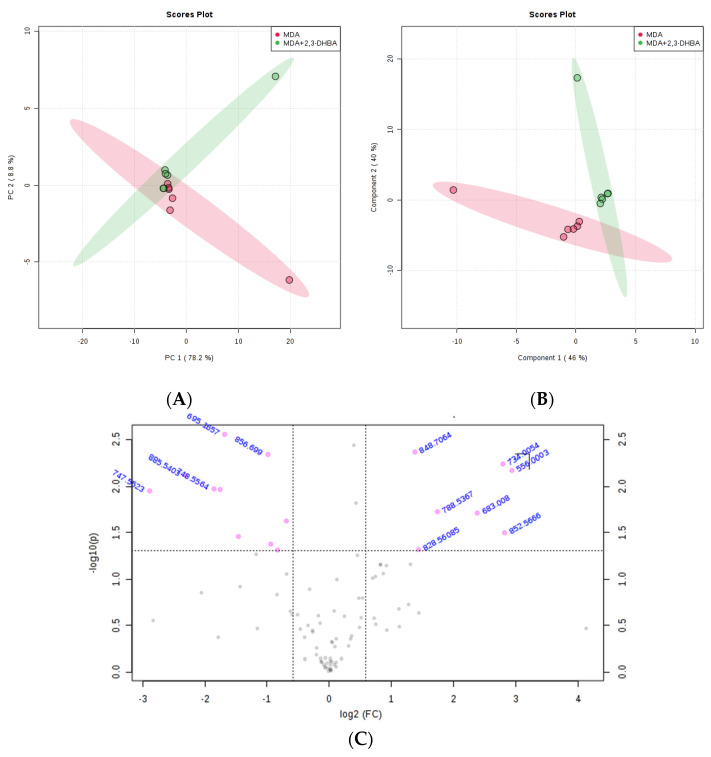
Effect of 2,3-DHBA exposure on the relative abundance of lipids in MDA-MB-231 cells. MDA-MB-231 cells were treated with either DMSO (control) or the IC_50_ concentration of 2,3-DHBA (5.48 mM) for NIM. Forty-eight hours after treatment, cells were collected, subjected to Bligh–Dyer extraction, and analyzed using ESI-MS. (**A**) PCA of lipids extracted from DMSO-treated cells (red) and 2,3-DHBA-treated cells (green). Each data point represents an individual replicate. The ovals represent 95% confidence intervals. (**B**) PLS-DA of lipids extracted from DMSO-treated cells (red) and 2,3-DHBA-treated cells (green). Each data point represents an individual replicate. The ovals represent 95% confidence intervals. (**C**) Volcano plot analysis comparing the log fold change of each *m*/*z* value (X-axis) to the significance value (Y-axis) for pairwise comparisons between the DMSO-treated (control) and 2,3-DHBA treatment groups. The identity of select *m*/*z* values is indicated next to each data point. Pink dots indicate significantly changed lipids, while gray dots indicate lipids that did not change significantly. All features presented are considered significant based on a fold change threshold of ≥2.00 and *t*-tests where *p* ≤ 0.05.

The MetaboAnalyst 4.0 (http://www.metaboanalyst.ca/; accessed on 21 June 2025) and LIPID MAPS (http://www.lipidmaps.org/; accessed on 24 June 2025) databases were used to process the ESI-MS data according to the specifications in the experimental section. MetaboAnalyst 4.0 was used to evaluate differences between groups using Excel data derived from the spectra shown in Figure 2, Figure 3, Figure 4 and Figure 5. Multivariate unsupervised principal component analysis (PCA) and partial least squares discriminant analysis (PLS-DA) of spectral data comparing the control and 2,3-DHBA-treated groups showed distinct clustering within both the MCF-7 cell lipidome (Figure 2A–C and Figure 3A–C) and MDA-MB-231 cell lipidome (Figure 4A–C and Figure 5A–C). Score plots for both PIM (Figure 2A,B and Figure 4A,B) and NIM (Figure 3A,B and Figure 5A,B) demonstrated clear separation between the control and 2,3-DHBA treatment groups. Volcano plot analysis, which combines *t*-test results (Figures S17, S19, S21 and S23) and the fold change (Figures S18, S20, S22 and S24) to identify lipid species with significant alterations between groups, was performed using the numerical mass-to-charge (*m*/*z*) ratios. This analysis compares the log fold change of each *m*/*z* value (X-axis) to the significance value (Y-axis). Volcano plot analysis of significantly altered *m*/*z* values for pairwise comparisons between the DMSO-treated (control) and 2,3-DHBA treatment groups was conducted for the MCF-7 cell lipidome in both PIM and NIM (Figure 2C and Figure 3C) and for the MDA-MB-231 cell lipidome in both PIM and NIM (Figure 4C and Figure 5C).

To determine the identity of the significant *m*/*z* values from the volcano plot, we consulted the LIPID MAPS database (http://www.lipidmaps.org/; accessed 24 June 2025), and the structures and classes of corresponding lipid species were determined through MS/MS verification. The results are presented for MCF-7 cells in positive ion mode (Table 2) and NIM (Table 3) and for MDA-MB-231 cells in PIM (Table 4) and NIM (Table 5).

**Figure 6:**
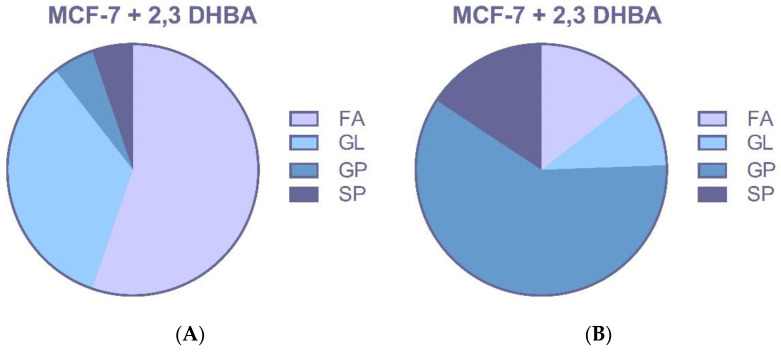
MCF-7 cells were treated with the IC_50_ of 2,3-DHBA (8.61 mM). Forty-eight hours after the treatment, cells were collected, subjected to Bligh–Dyer extraction, and analyzed using ESI–MS. Changed lipid classes: (**A**) PIM; (**B**) NIM. (FA: Fatty acid, GL: Glycerolipid, GP: Glycerophospholipid, SP: Sphingolipid).

**Figure 7:**
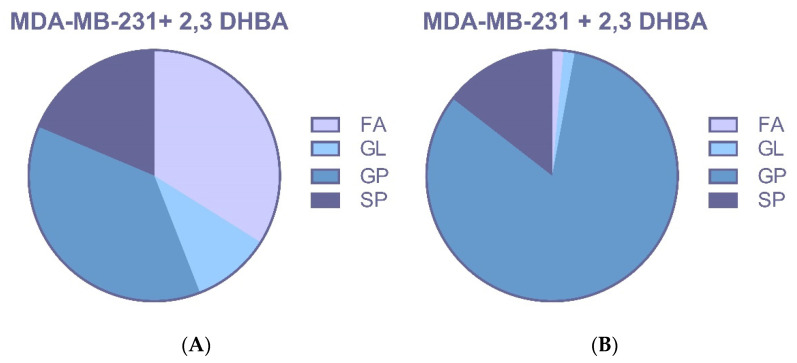
MDA-MB-231 cells were treated with the IC_50_ of 2,3-DHBA (5.48 mM). Forty-eight hours after the treatment, cells were collected, subjected to Bligh–Dyer extraction, and analyzed using ESI–MS. Changed lipid classes: (**A**) PIM; (**B**) NIM. (FA: Fatty acid, GL: Glycerolipid, GP: Glycerophospholipid, SP: Sphingolipid).

Treatment with 2,3-DHBA induced alterations in multiple lipid classes, including fatty acids, glycerolipids, glycerophospholipids, and sphingolipids in breast cancer cells (Figure 6A,B and Figure 7A,B). The identification of these lipid class changes represents a valuable approach for investigating lipid profile modifications in cancer, providing a foundation for the development of novel biomarkers and therapeutic strategies.

## 4. Discussion

There is growing interest in anticarcinogenic compounds that are naturally present in plant foods. Phenolic compounds found primarily in vegetables and fruits are recognized for their diverse biological activities, including antioxidant, anti-radical, anticancer, anti-inflammatory, anti-mutagenic, antiproliferative, antiviral, chemoprotective, chemotherapeutic, and immune-enhancing properties [7,8,9]. Phenolic acids have become the focus of current research attention due to their widespread presence in plants and foods, as well as their significant biological and pharmacological properties [45]. Identification of specific active ingredients with chemoprotective effects may facilitate the development of foods enriched with these compounds. Population studies have linked fruit- and vegetable-rich diets to a lower risk for chronic conditions such as heart disease, cancer, diabetes, Alzheimer’s, and cataracts [46]. Cancer remains one of the leading causes of death worldwide. Although multiple treatment options exist, including radiotherapy, surgical interventions, immunotherapy, and chemotherapy, cancer continues to pose a serious clinical challenge with profound negative social and economic impacts on human life. For these reasons, discovering synthetic and/or natural compounds suitable for cancer treatment remains crucial.

Salicylic acid metabolites, including 2,3-DHBA and 2,5-dihydroxybenzoic acid (2,5-DHBA), along with derivatives such as 2,4-dihydroxybenzoic acid (2,4-DHBA) and 2,6-dihydroxybenzoic acid (2,6-DHBA), have been reported to inhibit cyclin-dependent kinase 1 (CDK1) enzyme activity against HCT-116 human colorectal carcinoma cells. Studies have shown that 2,3-DHBA and 2,6-DHBA do not inhibit CDK2 and CDK4 but effectively inhibit CDK6 activity. Another derivative, 2,4,6-trihydroxybenzoic acid (2,4,6-THBA), has been found to effectively inhibit CDK1, 2, 4, and 6 activities. Molecular docking studies have demonstrated that these compounds potentially interact with CDK1 [47]. Sankaranarayanan et al. (2020) investigated the effects of 2,3-DHBA and 2,5-DHBA metabolites on all CDKs involved in cell cycle regulation (CDK 1, 2, 4, and 6) and their effects on colony formation in three different cancer cell lines, including MDA-MB-231 breast cancer cells, HCT-116, and HT-29 colon cancer cells [48]. The study revealed that 2,3-DHBA and 2,5-DHBA inhibited CDK1 enzyme activity starting at a concentration of 500 μM, while CDK2 and CDK4 activity was inhibited only at higher concentrations (>750 μM). While 2,3-DHBA inhibited CDK6 enzyme activity at 250 μM, 2,5-DHBA required concentrations of >750 μM for inhibition. The study found that 2,5-DHBA effectively inhibited colony formation (for 21 days) in HCT-116 and HT-29 cancer cell lines at concentrations of 250–500 μM and in MDA-MB-231 breast cancer cells at approximately 100 μM. Conversely, 2,3-DHBA effectively inhibited colony formation only in MDA-MB-231 cells at approximately 500 μM for 21 days. Both aspirin and salicylic acid failed to inhibit the four CDKs and colony formation. Similarly to our results, MDA-MB-231 cells were treated with the 2,3-DHBA (125–1000 µM, 0.125–1 mM) for 48 h, and it was found that, in total, >96% of the cells were viable.

Based on these results, it was suggested that 2,3-DHBA and 2,5-DHBA may contribute to the chemoprotective properties of aspirin through CDK inhibition. Additional research by [49] examined the cytotoxic effects of caffeic acid and gallic acid on MCF-7 human breast cancer cells using the MTT method, investigating p53, Mcl-1, and p21 gene expression levels using RT-PCR. The IC_50_ values for caffeic acid and gallic acid in the concentration range of 5–200 μg/mL at 48 h were 170 μg/mL and 18.5 μg/mL, respectively, while the 72 h IC_50_ values were 159 μg/mL and 18 μg/mL, respectively [49]. Ref. [50] examined the cytotoxic effects of seven different derivatives of plant-derived hydroxybenzoic acid (HBA)—including 2,3-dihydroxybenzoic (2,3-DHB, pyrocatechuic), 2,4-dihydroxybenzoic (2,4-DHB, β-resorcylic), 2,5-dihydroxybenzoic (2,5-DHB, gentisic), 2,6-dihydroxybenzoic (2,6-DHB, γ-resorcylic acid), 3,4-dihydroxybenzoic (3,4-DHB, protocatechuic), 3,5-dihydroxybenzoic (3,5-DHB, α-resorcylic), and 3,4,5-trihydroxybenzoic (3,4,5-THB, gallic) acids—against MCF-7 and MDA-MB-231 human breast cancer cells using the neutral red (NR) method with increasing concentrations of hydroxybenzoic acids (0.02–5 mM) for forty-eight hours. The IC_50_ values of the tested compounds were found to be 0.36 mM to 4.77 mM [50]. Similarly to our results, the MDA-MB-231 cell line turned out to be more sensitive than MCF-7 cells; the IC_50_ value of 2,3-DHBA was found to be 4.10 mM against MDA-MB-231 human cancer cells, and the IC_50_ value was higher than 5 mM against MCF-7 human cancer cells.

The cytotoxic agent needs time to enter the cells and reach a concentration high enough to trigger a response. The rate of uptake can vary depending on the drug and the cell type. Lower concentrations might take longer to show an effect or might only be effective after prolonged exposure. As the exposure time increases, cells are exposed to the cytotoxic agent for longer, allowing for greater accumulation of damage, more complete inhibition of cellular processes, or the progression of cell death pathways. For many drugs, the cytotoxic effect (measured by parameters such as the half-maximal inhibitory concentration, or IC_50_) increases over time. For a given concentration, the percentage of dead or non-viable cells steadily increases from 24 h to 48 h to 72 h. This means that after a longer exposure, a lower concentration of the drug is needed to kill the same percentage of cells. The stability of the drug in the culture medium can affect long-term results. The cytotoxic agent might be unstable in the cell culture medium or rapidly metabolized by the cells. In some long-term treatments, the active concentration of the drug drops, allowing the surviving sub-population of cells to recover and begin to proliferate, leading to an increase in cell viability. This suggests that some cells have adapted and become resistant to the treatment. Changes in cytotoxicity observed at different exposure times reflect the complex interplay of drug concentration, cellular uptake, metabolism, activation of repair and cell death pathways, cell cycle progression, and the inherent heterogeneity of the cell population. Comprehensive time-dependent studies are essential for a thorough understanding of a compound’s cytotoxic profile. The effect of a substance on cell viability can evolve over time due to several complex biological and chemical processes. Because a single time point might miss a delayed effect or overestimate an acute, transient one, observing cytotoxicity at multiple time points (e.g., 24 h, 48 h, 72 h, or even longer) provides a more complete picture than a single-time-point measurement. While a dose–response curve shows the effect at a single time point, a time–response curve (or a series of dose–response curves at different time points) shows how the effect evolves over time for a given dose. For example, a drug that shows minimal toxicity at 24 h might be highly toxic at 72 h, which is crucial information for patient safety and efficacy. By understanding the time-dependent nature of cytotoxicity, one can better predict the effectiveness and safety of drugs and design optimal dosing schedules.

Therefore, the cytotoxic activity of 2,3-DHBA on MCF-7 and MDA-MB-231 cells was studied for 24 h, 48 h, and 72 h. It was observed that the IC_50_ values of 2,3-DHBA decreased with time against both MCF-7 and MDA-MB-231 breast cancer cells. Since lower IC_50_ values indicate higher cytotoxic activity, the in vitro cytotoxic activity of 2,3-DHBA increased with longer incubation times. MDA-MB-231 cells showed lower IC_50_ values compared to the MCF-7 cells at all exposure times, indicating that MDA-MB-231 cells are more sensitive to 2,3-DHBA treatment than MCF-7 cells. This may arise from the fact that the two breast cancer cells have different genetic properties that contribute to varying responses to 2,3-DHBA exposure.

Cancer is characterized by uncontrolled cell growth associated with increased cell migration resulting from enhanced proliferation, decreased apoptosis, and/or enhanced ability to migrate to neighboring tissues and metastasize to distant organs. Cell proliferation requires the replication of all macromolecular components during each cell division and necessitates increased lipid biosynthesis, producing bioactive molecules that function as signaling molecules in cancer metastasis regulation through lipid catabolism [21,26]. Therefore, abnormal lipid metabolism is recognized as a hallmark of cancer cells. Lipid metabolism has emerged from its perceived role as a simple housekeeping function to a central, targetable axis that drives tumorigenesis, metastasis, and therapeutic resistance [51]. Cancer cells do not merely alter lipid metabolism as a consequence of transformation; they actively rewire these pathways to provide the necessary energy, structural components for membrane biogenesis, and a rich repertoire of signaling molecules essential for their malignant phenotype [52]. This dysregulation is particularly pronounced in aggressive cancer subtypes, such as triple-negative breast cancer (TNBC), which leverages metabolic plasticity to fuel rapid proliferation, invasion, and adaptation to the harsh tumor microenvironment [52]. The three classical classes of lipids—fatty acids, phospholipids, and cholesterol—increase significantly in cancer cells and tumors and are actively biosynthesized. Studies have demonstrated that fatty acid synthase expression and activity are extremely low in non-malignant adult tissues but significantly increased in certain solid and aggressive cancers [53]. Many cancers exhibit increased activity of choline kinase, a key enzyme responsible for producing the lipid phosphatidylcholine. This increase is triggered by cancer-promoting elements such as growth factors and the oncogene protein ras. This is significant because phosphatidylcholine, like all lipids, is ultimately built from fatty acid components [54]. Active sterol biosynthesis remains an essential metabolic component of cell proliferation. Transcriptional profiling with microarray analysis has shown that numerous genes in the cholesterol biosynthesis pathway are significantly overexpressed in treatment-resistant cancers [21]. Cholesterol biosynthesis occurs much earlier than DNA synthesis, and inhibiting cholesterol biosynthesis slows cell growth, indicating a link between cholesterol and DNA synthetic pathways [55].

Lipid metabolism is highly complex and regulated by intricate signaling networks within cells. Enhanced understanding of lipid metabolism in cancer treatment and apoptosis could contribute to developing improved cancer therapies. Lipid metabolism in cancer cells remains largely unknown. Cancer lipidomics applications include the detection and classification of tumor cells and tissues, as well as the evaluation of anticancer therapy. Integration of lipidomic strategies into cancer research has enabled an understanding of lipid functions in biological samples and their molecular mechanisms in disease initiation and development. Additionally, altered lipid profiles in biological samples have been examined for biomarker identification in cancer research [56]. Recently, lipidomic studies comparing lipid profiles of normal and cancerous tissues and cells have provided more detailed information on lipid metabolism. These studies could prove useful in identifying clinical biomarkers for early diagnosis and determining cancer treatment efficacy [19,57].

Free fatty acids (FFAs) play key roles in numerous metabolic pathways, acting as substrates in energy metabolism and as intermediate products in signal transmission. Various potential biomarkers have been predicted for certain diseases, including diabetes, Alzheimer’s disease, pancreatic cancer, and autism, based on changes in peripheral blood FFA concentrations. A study using serum samples from 140 breast cancer patients and 202 healthy controls found that serum FFA concentrations in breast cancer patients were significantly decreased compared to controls [58]. Specifically, C16:1, C18:3, C18:2, C20:4, and C22:6 fatty acid types differed significantly between breast cancer patients and healthy individuals and may serve as useful fatty acid biomarkers for breast cancer diagnosis.

Beyond their essential role as the building blocks of cell membranes, phospholipids (PLs) are diverse molecules that actively regulate a wide array of biological functions, such as signal transduction, cell adhesion, and energy storage. Because they are central to cellular health, significant changes in the metabolism or composition of PLs in tissues or bodily fluids such as blood and urine have been linked to cancer and other pathologies. Changes in the composition, distribution, and metabolism of various PLs in cells, tissues, and bodily fluids, including blood and urine, have been associated with cancer and other diseases [56].

Sphingolipids (SLs) are polar lipids with a sphingoid backbone containing different subgroups, including sphingomyelin (SM), sulfatides, ceramides, cerebrosides, and gangliosides. SLs function as cellular membrane components and bioactive compounds with important biological functions. Cancer cells might gain a survival advantage by changing the way they process sphingolipids. Altered sphingolipid metabolism provides a survival advantage to cancer cells by disrupting key membrane signals. The balance of ceramide, sphingomyelin, and cholesterol in lipid rafts controls pathways for apoptosis, survival, and migration. Therefore, the dysregulation of these lipids—a hallmark of cancer’s metabolic changes—can directly influence cell transformation and tumor growth [59].

Glycerophospholipids (GPLs) are generally abundant in cell membranes and constitute an important lipid class in cancer lipidomic research. More et al. (2017) investigated potential phospholipid species that could differentiate breast cancer from benign and healthy controls in serum samples [60]. The study applied liquid chromatography–multiple reaction monitoring–mass spectrometry to serum samples from 28 breast cancer patients and controls, observing changes in phospholipids. Both multivariate and univariate statistical analyses were performed to investigate phospholipid changes associated with 28 breast cancer patients and controls, with differences confirmed by LC-MS/MS. Out of 200 measured phospholipids, 25 were significantly altered (*p* < 0.05) in breast cancer patients versus benign and healthy controls. The comparison between cancer patients and healthy controls identified 12 changed phospholipids (6 increased, 6 decreased), while the comparison between cancer and benign samples revealed increased levels of 6 phospholipids in cancer tissue. The study identified phospholipid types, including PE (14:1/16:0), PC (18:0/18:0), LPE 14:0, and PE (20:0/22:2), as significantly different between breast cancer patients and healthy controls, suggesting that these lipid types may serve as phospholipid biomarkers for differentiating breast cancer from benign and healthy controls and monitoring breast cancer progression. Jiang et al. (2017) conducted a study to identify new biomarkers for early breast cancer detection using UPLC-QTOF/MS analysis of 78 plasma samples (37 breast cancer samples and 41 healthy controls) [61]. They identified a total of 847 lipid types: 1 SL, 6 PKs, 18 PRs, 68 STs, 77 FAs, 91 SPs, 265 GLs, and 321 GPs. PC (20:2/20:5), PC (22:0/24:1), TG (12:0/14:1), and DG (18:1/18:2) lipid species showed higher levels in breast cancer samples compared to healthy controls, while PE (15:0/19:1) and N-palmitoyl proline were lower in breast cancer samples.

Changes in profiles of free fatty acids (FFAs), phospholipids (PLs), and sphingolipids (SLs) have been associated with disease, suggesting that specific lipids may play roles in cancer initiation, progression, and evolution. The changes in lipid species seen in these studies are probably not isolated events. Instead, they likely reflect the widespread metabolic disruption caused by cancer, which impacts everything from gene expression to the enzymatic activities that fuel the disease’s progression. However, it is likely that the altered lipid profiles seen in these studies are a direct consequence of cancer’s underlying metabolic and enzymatic dysregulation, which is also responsible for changes in cell morphology, gene expression, and disease progression. The identification of phospholipid classes and structures represents a valuable tool for investigating lipid profile changes in cancer, providing a foundation for developing new biomarkers and therapeutic strategies. In our study, the effects of 2,3-DHBA on lipid profiles of MCF-7 and MDA-MB-231 breast cancer cells were investigated using an untargeted lipidomic approach. Treatment with 2,3-DHBA induced alterations in lipid classes, including fatty acids, glycerolipids, glycerophospholipids, and sphingolipids, in breast cancer cells. Following 2,3-DHBA application to MCF-7 cancer cells, changes were observed in 46 fatty acid (FA), 30 glycerolipid (GL), 105 glycerophospholipid (GP), and 29 sphingolipid (SP) types (Figure 6). Following 2,3-DHBA application to MDA-MB-231 cancer cells, changes were observed in 21 FA, 7 GL, 79 GP, and 21 SP types (Figure 7). The extensive changes observed in the glycerolipid and glycerophospholipid profiles following 2,3-DHBA treatment suggest a dual-pronged assault on the cancer cell’s infrastructure. These lipids are not passive structural components but are dynamically regulated to control both the physical properties of cellular membranes and the propagation of critical pro-survival signals. The data indicate that 2,3-DHBA simultaneously compromises the physical integrity required for metastasis and dismantles the signaling networks that promote proliferation and survival. The cell membrane is a dynamic entity whose biophysical properties—such as fluidity, curvature, and charge—are dictated by its phospholipid composition. Cancer cells, particularly metastatic ones, actively remodel their membranes to facilitate processes such as migration and invasion [62]. The present study’s finding that 2,3-DHBA induces sweeping changes in numerous glycerophospholipid species in both MCF-7 and MDA-MB-231 cells points to a direct interference with this pro-malignant membrane architecture. Phosphatidylcholine (PC) is the most abundant phospholipid in eukaryotic membranes, and its metabolism is frequently elevated in cancer to support the demands of rapid cell proliferation [63]. Studies have shown that the total content of PC and phosphatidylethanolamine (PE) increases with advancing breast cancer tumor grade, highlighting the importance of phospholipid synthesis in tumor progression [64]. However, the identity of the fatty acyl chains within these phospholipids is of paramount importance. Metastatic TNBC tumors, for instance, are characterized by an enrichment of phospholipids containing long-chain polyunsaturated fatty acids (PUFAs). This increase in unsaturation enhances membrane fluidity, which, in turn, promotes the activation of signaling molecules such as integrin β1 and facilitates metastasis [62]. The lipidomic data from this study reveal significant alterations in a multitude of GP species, such as those detected at *m*/*z* 786.599 and *m*/*z* 832.241 in MCF-7 cells, as well as *m*/*z* 758.5683 and *m*/*z* 804.5512 in MDA-MB-231 cells. These shifts suggest a profound disruption in the balance of phospholipid species. For example, the ratio of PUFA-containing PCs to monounsaturated fatty acid (MUFA)-containing PCs (such as PC(16:0/18:1), a common species with a monoisotopic mass of 760.08 Da) is a critical determinant of cell cycle progression. By altering the available pool of fatty acids or interfering with the activity of acyltransferases responsible for their incorporation into the glycerol backbone, 2,3-DHBA likely forces the synthesis of phospholipids with a suboptimal acyl chain composition. This forced remodeling would directly alter membrane fluidity and rigidity, thereby disrupting the formation of specialized signaling platforms like lipid rafts and crippling the cell’s physical capacity for invasion and migration. This provides a direct mechanistic link between the observed lipidomic changes and the potent anti-cancer activity of 2,3-DHBA, suggesting that its effect extends beyond simple cytotoxicity to include anti-metastatic potential.

The significant changes in the glycerolipid (GL) category reported in the manuscript, exemplified by species at *m*/*z* 977.775 in MCF-7 cells and *m*/*z* 979.7644 in MDA-MB-231 cells (which correspond to high-mass TAGs), point to the disruption of a critical metabolic nexus involving two key lipid classes: the signaling second messenger diacylglycerol (DAG) and the energy-storage molecule triacylglycerol (TAG). DAG is a pivotal lipid second messenger generated at the plasma membrane that orchestrates a multitude of pro-survival and pro-proliferative signals. It functions by recruiting and activating a host of downstream effector proteins, most notably protein kinase C (PKC) isozymes and Ras guanyl-releasing proteins (RasGRPs), which, in turn, activate pathways such as the MAPK/ERK and PI3K/Akt/mTOR cascades [65]. To maintain signaling fidelity, DAG levels are tightly controlled. This termination is primarily accomplished by diacylglycerol kinases (DGKs), which phosphorylate DAG to generate phosphatidic acid (PA) [66]. Several DGK isoforms, particularly DGK α, are overexpressed in various cancers, including breast cancer, where they function to dampen excessive DAG signaling that might otherwise trigger cell death while simultaneously producing pro-mitogenic PA [65]. Consequently, inhibition of DGK α has emerged as a promising therapeutic strategy to disrupt tumor growth [67]. Concurrently, cancer cells must manage their energy resources to survive metabolic stress. They accomplish this by esterifying excess fatty acids into TAGs, which are stored in cytoplasmic lipid droplets (LDs) [68]. These LDs are not merely inert storage depots but dynamic organelles that buffer against lipotoxicity and provide a ready source of fuel for fatty acid oxidation (FAO) when other nutrients are scarce [68]. This capacity for TAG storage is especially critical for therapy-resistant breast cancer cells, which exhibit a metabolic phenotype shifted toward increased lipid storage to sustain survival [69]. The widespread changes in GLs observed after 2,3-DHBA treatment suggest a profound disruption of this interconnected DAG-PA-TAG network. By interfering with the enzymes that regulate this hub, 2,3-DHBA could be executing a devastating dual attack. On one hand, by modulating the levels or activity of enzymes such as phospholipase C (generates DAG) or DGKs (consumes DAG), the compound could starve the cell of essential pro-survival DAG signals, effectively silencing downstream PKC and Akt/mTOR activity. On the other hand, by disrupting the synthesis or breakdown of TAGs, 2,3-DHBA could prevent the cell from building its emergency energy reserves in LDs. This would render the cancer cells acutely vulnerable to nutrient deprivation and oxidative stress, providing a powerful explanation for the observed cytotoxicity.

It was observed that the most profound lipidomic alterations induced by 2,3-DHBA occur within the sphingolipid class. The data strongly suggest that a primary mechanism of action for the compound is the strategic rearrangement of the “sphingolipid balance”—the delicate equilibrium between two key lipids: ceramide, which induces apoptosis, and sphingosine-1-phosphate (SIP), which supports cell survival [70]. By shifting this balance decisively toward cell death, 2,3-DHBA appears to turn the cell’s own metabolic machinery into a death tool. Ceramide sits at the absolute center of sphingolipid metabolism, serving as the precursor for all complex sphingolipids and acting as a potent signaling molecule in its own right [71]. An accumulation of intracellular ceramide is a well-established trigger for apoptosis, cell cycle arrest, and senescence, making it a key tumor suppressor lipid [72]. The therapeutic potential of harnessing this pathway is significant, with strategies such as ceramide nanoliposome (CNL) delivery being explored for treating aggressive cancers such as TNBC [73]. The role of ceramide in breast cancer, however, is controversial. While it is functionally pro-apoptotic, several studies have reported a paradoxical increase in total ceramide levels in malignant breast cancer tissue compared to normal tissue [74]. This apparent contradiction is resolved when considering two critical factors: the specificity of ceramide species based on N-acyl chain length and the metabolic flux through the pathway. Different ceramide species possess distinct biological functions. For instance, C16:0-ceramide has been specifically associated with lymph node positivity and metastatic potential in breast cancer, while very-long-chain ceramides (e.g., C24:0 and C24:1) are also dysregulated [74]. Our lipidomic data, showing significant alterations in sphingolipid species across a wide mass range (e.g., *m*/*z* 606.4846, 634.8158, 750.5376 in MCF-7 cells), strongly support the notion that 2,3-DHBA is altering the profile of ceramide species, not just the total amount.

## 5. Conclusions

The present study demonstrates that 2,3-DHBA, a naturally occurring phenolic acid, exerts significant dose- and time-dependent cytotoxic effects against both the luminal A (MCF-7) and the more aggressive basal-like TNBC (MDA-MB-231) human breast cancer cell lines. Our results showed that 2,3-DHBA has significantly higher selectivity and is more cytotoxic against MDA-MB-231 breast cancer cells. Critically, the untargeted lipidomic analysis reveals that this cytotoxicity is inextricably linked to cell-type-specific perturbations. Following treatment with 2,3-DHBA, significant alterations were observed across all major lipid categories, including fatty acids (FAs), glycerolipids (GLs), glycophospholipids (GPs), and sphingolipids (SPs).

Our findings provide a compelling case study of how this lipid-centric vulnerability can be exploited for therapeutic gain. Comprehensive analysis of the lipidomic data reveals that 2,3-dihydroxybenzoic acid is not only a conventional cytotoxic agent but also a significant modulator of cancer lipid metabolism. The findings suggest that 2,3-DHBA, particularly the aggressive MDA-MB-231 subtype, comprehensively rewires the cellular lipidome. This rewiring appears to result in the following: (1) compromised membrane integrity through the perturbation of glycerophospholipid composition, which likely impairs metastatic potential; (2) attenuated pro-survival signaling via the disruption of the DAG/PKC and SphK1/S1P axes; and (3) a decisive shift in the sphingolipid rheostat toward cell death, driven by the accumulation of cytotoxic ceramides.

Our research will incorporate proteomic and genomic studies based on lipid types that change with anticancer activity observed following 2,3-DHBA application, demonstrating a pathway for developing targeted-lipidomics-based treatment strategies in breast cancer therapy. We can more clearly emphasize that the study serves as a foundational work and a starting point for more focused mechanistic and pathway analyses in the future.

## Figures and Tables

**Table 1:** IC_50_ values of 2,3-DHBA at 24 h, 48 h, and 72 h against MCF-7 and MDA-MB-231 human breast cancer cells and L-929 healthy mouse fibroblast cells.

	(IC_50_, mM) ^a^					
	24 h	SI-24 h	48 h	SI-48 h	72 h	SI-72 h
MCF-7	13.35 ± 0.14	>1.49	8.61 ± 0.21	1.90	4.20 ± 0.12	3.45
MDA-MB-231	10.52 ± 0.20	>1.90	5.84 ± 0.10	2.80	4.09 ± 0.10	3.54
L-929 ^b^	>20		16.35 ± 0.13		14.51 ± 0.10	

The ^a^ IC_50_ value was calculated as the concentration value that caused a 50% decrease in cell viability in the MTT experiment performed at 24 h, 48 h, and 72 h after the compound applications, and the results are given as ±SD (*n* = 6). ^b^ Healthy cell. The selective toxicity of the complexes was quantified by calculating a selectivity index. This index was obtained by dividing the IC_50_ value measured in a healthy cell line by the IC_50_ value from a cancer cell line after 24, 48, and 72 h of exposure.

**Table 2:** Significantly altered lipid species between the control (MCF-7 cells treated with DMSO) and 2,3-DHBA-treated cells (MCF-7 cells treated with the IC_50_ concentration of 2,3-DHBA for 48 h) for PIM (2,3-DHBA treatment versus control).

*m*/*z*	Lipid Class Code ^a^	Lipid Class Name	Formula ^b^
208.18	LMFA08010014	Faty acid	C_8_H_18_NS_2_
211.178	LMFA00000040	Faty acid	C_5_H_12_O_2_S
	LMFA01030244	Faty acid	C_13_H_23_O_2_
	LMFA01030963	Faty acid	C_6_H_5_O_4_Cl_2_
	LMFA01150037	Faty acid	C_12_H_19_O_3_
	LMFA01170107	Faty acid	C_6_H_11_O_8_
	LMFA07010604	Faty acid	C_13_H_23_O_2_
215.151	LMFA01031065	Faty acid	C_14_H_15_O_2_
	LMFA01050168	Faty acid	C_12_H_23_O_3_
239.164	LMFA01060185	Faty acid	C_14_H_23_O_3_
	LMFA01150051	Faty acid	C_12_H_15_O_5_
	LMFA12000368	Faty acid	C_11_H_11_O2S_2_
262.193	LMFA07070041	Faty acid	C_12_H_24_NO_5_
	LMFA07070073	Faty acid	C_12_H_24_NO_5_
	LMFA07070081	Faty acid	C_11_H_20_NO_6_
	LMFA08030039	Faty acid	C_14_H_16_NO_4_
270.191	LMFA01120009	Faty acid	C_14_H_24_NO_4_
273.189	LMFA01030510	Faty acid	C_18_H_25_O_2_
274.181	LMFA07070065	Faty acid	C_12_H_20_NO_6_
289.201	LMFA01031034	Faty acid	C_18_H_25_O_3_
	LMFA12000356	Faty acid	C_15_H_13_O_2_S_2_
786.599	LMGP01010764	Glycerophospholipid	C_44_H_85_NO_8_P
	LMGP01020225	Glycerophospholipid	C_45_H_89_NO_7_P
	LMGP01080001	Glycerophospholipid	C_46_H_77_NO_7_P
	LMGP02010709	Glycerophospholipid	C_45_H_73_NO_8_P
	LMGP03010136	Glycerophospholipid	C_42_H_77_NO_1_0P
	LMGP03030066	Glycerophospholipid	C_43_H_81_NO_9_P
832.241	LMGP01010847	Glycerophospholipid	C_48_H_83_NO_8_P
	LMGP03010454	Glycerophospholipid	C_46_H_75_NO_10_P
	LMGP20010046	Glycerophospholipid	C_44_H_83_NO_11_P
875.266	LMGP01010663	Glycerophospholipid	C_50_H_101_NO_8_P
	LMGP03010525	Glycerophospholipid	C_48_H_93_NO_10_P
	LMSP02040010	Sphingolipid	C_57_H_112_NO_4_
977.775	LMGL03011720	Glycerolipid	C_64_H_113_O_6_
	LMGL03012263	Glycerolipid	C_65_H_101_O_6_
	LMGP06010691	Glycerophospholipid	C_53_H_102_O_13_P_13_P
978.769	LMGP13010007	Glycerophospholipid	C_46_H_82_N_3_O_15_P_2_
1080.8265	LMSP0502AA03	Sphingolipid	C_56_H_106_NO_18_

^a^ Volcano plot analysis; comparison of significantly changing *m*/*z* values (*p* ≤ 0.05) by comparing MCF-7 breast cancer cells with DMSO or 2,3-DHBA for 48 h PIM [M+H]^+^. ^b^ Confirmation of *m*/*z* values of significantly varying lipid species by scanning lipid maps.

**Table 3:** Significantly altered lipid species between the control (MCF-7 cells treated with DMSO) and 2,3-DHBA-treated cells (MCF-7 cells treated with the IC_50_ concentration of 2,3-DHBA for 48 h) for NIM (2,3-DHBA treatment versus control).

*m*/*z*	Lipid Classes Code ^a^	Lipid Class Name	Formula ^b^
205.0902	LMFA01050442	Fatty acid	C_7_H_9_O_7_
	LMFA01070034	Fatty acid	C_11_H_9_O_4_
	LMFA01130001	Fatty acid	C_8_H_13_O_2_S_2_
	LMFA12000343	Fatty acid	C_11_H_9_O_2_S
217.1225	LMGL03012615	Glycerolipid	C_9_H_13_O_6_
229.1412	LMFA12000352	Fatty acid	C_13_H_9_S_2_
255.2317	LMFA01010001	Fatty acid	C_16_H_31_O_2_
	LMFA01060047	Fatty acid	C_15_H_27_O_3_
	LMFA01150060	Fatty acid	C_12_H_15_O_6_
	LMFA05000009	Fatty acid	C_17_H_35_O
	LMFA06000252	Fatty acid	C_16_H_31_O_2_
355.0776	LMFA01030186	Fatty acid	C_24_H_35_O_2_
	LMFA01040040	Fatty acid	C_19_H_31_O_6_
	LMFA01050077	Fatty acid	C_22_H_43_O_3_
	LMFA01170036	Fatty acid	C_21_H_39_O_4_
	LMFA03010069	Fatty acid	C_20_H_35_O_5_
	LMFA05000082	Fatty acid	C_23_H_47_O_2_
	LMFA06000269	Fatty acid	C_26_H_27_O
	LMFA08020219	Fatty acid	C_19_H_35_N_2_O_4_
	LMFA08020264	Fatty acid	C_20_H_39_N_2_O_3_
539.3697	LMFA03120034	Fatty acid	C_27_H_36_O_9_Cl
	LMFA08020206	Fatty acid	C_30_H_39_N_2_O_7_
551.3998	LMFA01030844	Fatty acid	C_38_H_63_O_2_
	LMGL02010360	Glycerolipid	C_34_H_63_O_5_
556.0005	LMFA01030830	Fatty acid	C_38_H_67_O_2_
605.4828	LMGL02030029	Glycerolipid	C_41_H_65_O_3_
	LMGP10010048	Glycerophospholipid	C_32_H_62_O_8_P
	LMGP10020004	Glycerophospholipid	C_33_H_66_O_7_P
	LMGP20070024	Glycerophospholipid	C_30_H_54_O_10_P
	LMGP20070032	Glycerophospholipid	C_29_H_50_O_11_P
606.3652	LMGP20020016	Glycerophospholipid	C_28_H_49_NO_11_P
	LMGP20020023	Glycerophospholipid	C_29_H_53_NO_10_P
	LMGP20020028	Glycerophospholipid	C_30_H_57_NO_9_P
	LMGP20040019	Glycerophospholipid	C_28_H_49_NO_11_P
606.4846	LMGP01050057	Glycerophospholipid	C_32_H_65_NO_7_P
	LMGP02010058	Glycerophospholipid	C_31_H_61_NO_8_P
	LMSP00000016	Sphingolipid	C_40_H_80_NO_2_
	LMSP02010017	Sphingolipid	C_39_H_76_NO_3_
	LMSP02010080	Sphingolipid	C_38_H_72_NO_4_
	LMGL02010007	Glycerolipid	C_37_H_69_O_5_
607.4824	LMGL02010329	Glycerolipid	C_39_H_59_O_5_
	LMGP20060022	Glycerophospholipid	C_29_H_52_O_11_P
608.9148	LMSP02010072	Sphingolipid	C_38_H_74_NO_4_
	LMGP03060024	Glycerophospholipid	C_30_H_59_NO_9_P
	LMGP20020017	Glycerophospholipid	C_28_H_51_NO_11_P
615.5169	LMFA08020175	Fatty acid	C_25_H_47_N_6_O_8_
	LMGL02010063	Glycerolipid	C_39_H_67_O_5_
	LMGP10010051	Glycerophospholipid	C_33_H_60_O_8_P
	LMGP10030006	Glycerophospholipid	C_34_H_64_O_7_P
	LMSP04000001	Sphingolipid	C_34_H_68_N_2_O_5_P
616.5143	LMGL02070005	Glycerolipid	C_40_H_71_O_4_
	LMGP20020040	Glycerophospholipid	C_30_H_51_NO_10_P
	LMSP00000022	Sphingolipid	C_34_H_66_NO_6_S
	LMSP02050002	Sphingolipid	C_34_H_67_NO_6_P
617.5099	LMGP06050026	Glycerophospholipid	C_29_H_46_O_12_P
	LMGP10020007	Glycerophospholipid	C_34_H_66_O_7_P
	LMGP20070002	Glycerophospholipid	C_31_H_54_O_10_P
	LMSP03020066	Sphingolipid	C_32_H_62_N_2_O_7_P
619.3821	LMGL02070007	Glycerolipid	C_41_H_63_O_4_
	LMGP06050006	Glycerophospholipid	C_29_H_48_O_12_P
	LMGP10010890	Glycerophospholipid	C_33_H_64_O_8_P
	LMGP10020006	Glycerophospholipid	C_34_H_68_O_7_P
	LMGP20070001	Glycerophospholipid	C_31_H_56_O_10_P
634.8158	LMGP01010001	Glycerophospholipid	C_33_H_65_NO_8_P
	LMGP02020022	Glycerophospholipid	C_34_H_69_NO_7_P
	LMGP20010012	Glycerophospholipid	C_31_H_57_NO_10_P
	LMGP20020043	Glycerophospholipid	C_30_H_53_NO_11_P
	LMGP20040010	Glycerophospholipid	C_29_H_49_NO_12_P
	LMSP00000015	Sphingolipid	C_42_H_84_NO_2_
	LMSP02010021	Sphingolipid	C_41_H_80_NO_3_
	LMSP02010093	Sphingolipid	C_40_H_76_NO_4_
638.8238	LMSP02020032	Sphingolipid	C_40_H_80_NO_4_
644.8418	LMGP02010365	Glycerophospholipid	C_34_H_63_NO_8_P
	LMGP02030010	Glycerophospholipid	C_35_H_67_NO_7_P
655.4137	LMFA13030004	Fatty acid	C_36_H_63_O_10_
	LMGP06050024	Glycerophospholipid	C_31_H_60_O_12_P
	LMGP10010083	Glycerophospholipid	C_36_H_64_O_8_P
	LMGP10020079	Glycerophospholipid	C_37_H_68_O_7_P
	LMSP03020033	Sphingolipid	C_36_H_68_N_2_O_6_P
656.8771	LMGP02010367	Glycerophospholipid	C_35_H_63_NO_8_P
	LMSP00000025	Sphingolipid	C_37_H_70_NO_6_S
666.0187	LMGP20040007	Glycerophospholipid	C_30_H_53_NO_13_P
	LMGP20040014	Glycerophospholipid	C_31_H_57_NO_12_P
	LMSP0501AA54	Sphingolipid	C_38_H_68_NO_8_
667.021	LMGP10010065	Glycerophospholipid	C_37_H_64_O_8_P
	LMGP20050014	Glycerophospholipid	C_31_H_56_O_13_P
683.0088	LMGP10010151	Glycerophospholipid	C_38_H_68_O_8_P
	LMGP20050015	Glycerophospholipid	C_31_H_56_O_14_P
706.8112	LMGP02010445	Glycerophospholipid	C_39_H_65_NO_8_P
	LMGP03010889	Glycerophospholipid	C_36_H_69_NO_10_P
	LMGP03020005	Glycerophospholipid	C_37_H_73_NO_9_P
	LMSP02020028	Sphingolipid	C_46_H_92_NO_3_
734.0059	LMGP02010533	Glycerophospholipid	C_41_H_69_NO_8_P
	LMGP03010029	Glycerophospholipid	C_38_H_73_NO_10_P
747.5135	LMGL03012646	Glycerolipid	C_47_H_87_O_6_
	LMGP04010002	Glycerophospholipid	C_40_H_76_O_10_P
	LMGP04020011	Glycerophospholipid	C_41_H_80_O_9_P
	LMGP10010039	Glycerophospholipid	C_43_H_72_O_8_P
	LMGP15040002	Glycerophospholipid	C_32_H_60_O_17_P
748.5144	LMGP20040036	Glycerophospholipid	C_36_H_63_NO_13_P
	LMGP01010447	Glycerophospholipid	C_42_H_71_NO_8_P
750.5376	LMSP03030150	Sphingolipid	C_38_H_73_NO_11_P
752.8087	LMGP01010506	Glycerophospholipid	C_42_H_75_NO_8_P
	LMGP01020026	Glycerophospholipid	C_43_H_79_NO_7_P
	LMGP01040092	Glycerophospholipid	C_44_H_83_NO_6_P
	LMGP02020037	Glycerophospholipid	C_43_H_79_NO_7_P
	LMGP03010131	Glycerophospholipid	C_40_H_67_NO_10_P
768.851	LMSP05010052	Sphingolipid	C_44_H_82_NO_9_
	LMSP0501AA31	Sphingolipid	C_45_H_86_NO_8_
	LMGP01011423	Glycerophospholipid	C_43_H_79_NO_8_P
	LMGP01020053	Glycerophospholipid	C_44_H_83_NO_7_P
	LMGP03010091	Glycerophospholipid	C_41_H_71_NO_10_P
	LMGP03020031	Glycerophospholipid	C_42_H_75_NO_9_P
770.8552	LMSP05010070	Sphingolipid	C_44_H_84_NO_9_
	LMGP01011360	Glycerophospholipid	C_43_H_81_NO_8_P
	LMGP01020188	Glycerophospholipid	C_44_H_85_NO_7_P
	LMGP01040088	Glycerophospholipid	C_46_H_77_NO_6_P
	LMGP02080002	Glycerophospholipid	C_45_H_73_NO_7_P
	LMGP03010153	Glycerophospholipid	C_41_H_73_NO_10_P
	LMGP03020029	Glycerophospholipid	C_42_H_77_NO_9_P
	LMGL00000134	Glycerolipid	C_46_H_76_NO_8_
773.5216	LMGP04110002	Glycerophospholipid	C_44_H_70_O_9_P
	LMGP06010024	Glycerophospholipid	C_39_H_66_O_13_P
	LMGP15040007	Glycerophospholipid	C_34_H_62_O_17_P
	LMSP05010076	Sphingolipid	C_44_H_86_NO_9_
	LMGP10010835	Glycerophospholipid	C_45_H_74_O_8_P
	LMGP04100003	Glycerophospholipid	C_42_H_78_O_10_P
	LMGL05010001	Glycerolipid	C_45_H_73_O_10_
774.7834	LMGP01010401	Glycerophospholipid	C_43_H_85_NO_8_P
	LMGP01020023	Glycerophospholipid	C_44_H_89_NO_7_P
	LMSP02040003	Sphingolipid	C_50_H_96_NO_4_
775.4956	LMGP04110003	Glycerophospholipid	C_44_H_72_O_9_P
	LMGP06010022	Glycerophospholipid	C_39_H_68_O_13_P
	LMGP15040006	Glycerophospholipid	C_34_H_64_O_17_P
775.5	LMGP04010037	Glycerophospholipid	C_42_H_80_O_10_P
	LMGP04020033	Glycerophospholipid	C_43_H_84_O_9_P
	LMGP10010585	Glycerophospholipid	C_45_H_76_O_8_P
776.5165	LMGP01010512	Glycerophospholipid	C_44_H_75_NO_8_P
	LMGP02020104	Glycerophospholipid	C_45_H_79_NO_7_P
	LMGP03010088	Glycerophospholipid	C_41_H_79_NO_10_P
	LMGP03020070	Glycerophospholipid	C_42_H_83_NO_9_P
786.8336	LMGP01010750	Glycerophospholipid	C_44_H_85_NO_8_P
	LMGP01020208	Glycerophospholipid	C_45_H_89_NO_7_P
	LMGP01080003	Glycerophospholipid	C_46_H_77_NO_7_P
	LMGP02010767	Glycerophospholipid	C_45_H_73_NO_8_P
	LMSP05010044	Sphingolipid	C_44_H_84_NO_10_
792.8503	LMGP01011460	Glycerophospholipid	C_45_H_79_NO_8_P
	LMGP01020066	Glycerophospholipid	C_46_H_83_NO_7_P
	LMGP03010160	Glycerophospholipid	C_43_H_71_NO_10_P
	LMGP03020088	Glycerophospholipid	C_44_H_75_NO_9_P
806.5769	LMGP01010645	Glycerophospholipid	C_46_H_81_NO_8_P
	LMGP02030091	Glycerophospholipid	C_47_H_85_NO_7_P
	LMGP03010043	Glycerophospholipid	C_44_H_73_NO_10_P
	LMGP20020004	Glycerophospholipid	C_45_H_77_NO_9_P
	LMSP03030154	Sphingolipid	C_42_H_81_NO_11_P
	LMSP06020001	Sphingolipid	C_42_H_80_NO_11_S
807.981	LMGL03012734	Glycerolipid	C_52_H_87_O_6_
	LMGP04010306	Glycerophospholipid	C_45_H_76_O_10_P
	LMGP04020083	Glycerophospholipid	C_46_H_80_O_9_P
	LMGP06010027	Glycerophospholipid	C_41_H_76_O_13_P
	LMGP06020009	Glycerophospholipid	C_42_H_80_O_12_P
	LMGP10010723	Glycerophospholipid	C_47_H_84_O_8_P
	LMGP20050035	Glycerophospholipid	C_39_H_68_O_15_P
	LMSP03030007	Sphingolipid	C_42_H_83_NO_11_P
885.541	LMGP06010010	Glycerophospholipid	C_47_H_82_O_13_P
886.5455	LMGP03010786	Glycerophospholipid	C_50_H_81_NO_10_P
964.7116	LMSP03030023	Sphingolipid	C_52_H_103_NO_12_P
1003.977	LMGL03012162	Glycerolipid	C_66_H_115_O_6_
	LMGL03012500	Glycerolipid	C_67_H_103_O_6_
1033.682	LMGL03012468	Glycerolipid	C_68_H_121_O_6_
	LMGL03012580	Glycerolipid	C_69_H_109_O_6_
1047.906	LMGL03012466	Glycerolipid	C_69_H_123_O_6_

^a^ Volcano plot analysis; comparison of significantly changing *m*/*z* values (*p* ≤ 0.05) by comparing MCF-7 breast cancer cells with DMSO or 2,3-DHBA for 48 h NIM [M+H]^−^. ^b^ Confirmation of *m*/*z* values of significantly varying lipid species by scanning lipid maps.

**Table 4:** Significantly altered lipid species between the control (MDA-MB-231 cells treated with DMSO) and 2,3-DHBA-treated cells (MDA-MB-231 cells treated with the IC_50_ concentration of 2,3-DHBA for 48 h) for PIM (2,3-DHBA treatment versus control).

*m*/*z*	Lipid Classes Code ^a^	Lipid Class Name	Formula ^b^
235.1762	LMFA01090008	Fatty acid	C_12_H_24_O_2_C_l_
	LMFA01090080	Fatty acid	C_9_H_16_O_2_
	LMFA01140064	Fatty acid	C_15_H_23_O_2_
	LMFA06000156	Fatty acid	C_14_H_19_O_3_
	LMFA12000354	Fatty acid	C_12_H_11_OS_2_
245.1809	LMFA00000009	Fatty acid	C_12_H_25_N_2_O_3_
	LMFA01030703	Fatty acid	C_16_H_21_O_2_
	LMFA01050445	Fatty acid	C_10_H_17_N_2_O_5_
	LMFA01150045	Fatty acid	C_14_H_13_O_4_
	LMFA01170010	Fatty acid	C_13_H_25_O_4_
	LMFA05000019	Fatty acid	C_17_H_25_O
285.2044	LMFA01050459	Fatty acid	C_18_H_21_O_3_
	LMFA01150068	Fatty acid	C_14_H_21_O_6_
	LMFA12000363	Fatty acid	C_12_H_13_O_4_S_2_
321.231	LMFA01030678	Fatty acid	C_22_H_25_O_2_
	LMFA01090112	Fatty acid	C_16_H_18_O_2_
511.3819	LMGP20070018	Glycerophospholipid	C_23_H_44_O_10_P
512.3858	LMGP03010021	Glycerophospholipid	C_22_H_43_NO_10_P
	LMGP03050030	Glycerophospholipid	C_23_H_47_NO_9_P
612.3732	LMGP03060022	Glycerophospholipid	C_30_H_63_NO_9_P
	LMGP20040016	Glycerophospholipid	C_27_H_51_NO_12_P
	LMSP02020031	Sphingolipid	C_38_H_78_NO_4_
614.181	LMSP02030033	Sphingolipid	C_37_H_76_NO_5_
619.443	LMFA00000026	Fatty acid	C_24_H_44_O_5_SC_l5_
	LMFA00000049	Fatty acid	C_32_H_59_O_11_
	LMFA08020171	Fatty acid	C_27_H_51_N_6_O_10_
	LMFA13010023	Fatty acid	C_34_H_67_O_9_
	LMGP06050026	Glycerophospholipid	C_29_H_48_O_12_P
	LMGP10010072	Glycerophospholipid	C_33_H_64_O_8_P
	LMGP10020007	Glycerophospholipid	C_34_H_68_O_7_P
	LMGP20070002	Glycerophospholipid	C_31_H_56_O_10_P
	LMSP03020066	Sphingolipid	C_32_H_64_N_2_O_7_P
758.5683	LMGP01010585	Glycerophospholipid	C_42_H_81_NO_8_P
	LMGP01020201	Glycerophospholipid	C_43_H_85_NO_7_P
	LMGP02040014	Glycerophospholipid	C_45_H_77_NO_6_P
	LMGP03030038	Glycerophospholipid	C_41_H_77_NO_9_P
	LMSP02050010	Sphingolipid	C_44_H_89_NO_6_P
	LMSP05010059	Sphingolipid	C_43_H_84_NO_9_
	LMSP0501AA20	Sphingolipid	C_44_H_88_NO_8_
804.5512	LMGP00000048	Glycerophospholipid	C_43_H_83_NO_10_P
	LMGP01010696	Glycerophospholipid	C_46_H_79_NO_8_P
	LMGP01010975	Glycerophospholipid	C_45_H_91_NO_8_P
	LMGP01020060	Glycerophospholipid	C_46_H_95_NO_7_P
	LMGP02030092	Glycerophospholipid	C_47_H_83_NO_7_P
	LMGP03020015	Glycerophospholipid	C_44_H_87_NO_9_P
	LMSP02040004	Sphingolipid	C_52_H_102_NO_4_
	LMSP05010083	Sphingolipid	C_45_H_90_NO_10_
873.2717	LMGL03016884	Glycerolipid	C_55_H_69_O_9_
978.7616	LMGP13010007	Glycerophospholipid	C_46_H_82_N_3_O_15_P_2_
	LMGP13010007	Glycerophospholipid	C_46_H_82_N_3_O_15_P2
979.7644	LMGL03011721	Glycerolipid	C_64_H_115_O_6_
	LMGL03012217	Glycerolipid	C_65_H_103_O_6_
	LMGL03015976	Glycerolipid	C_64_H_115_O_6_
	LMGL03016504	Glycerolipid	C_65_H_103_O_6_
	LMGP06010915	Glycerophospholipid	C_53_H_104_O_13_P
1019.787	LMGL03012213	Glycerolipid	C_67_H_119_O_6_
	LMSP0601AA02	Sphingolipid	C_53_H_99_N_2_O_16_
1020.79	LMSP05010036	Sphingolipid	C_58_H_102_NO_13_
1080.829	LMSP0502AA03	Sphingolipid	C_56_H_106_NO_18_

^a^ Volcano plot analysis; comparison of significantly changing *m*/*z* values (*p* ≤ 0.05) by comparing MDA-MB-231 breast cancer cells with DMSO or 2,3-DHBA for 48 h PIM [M+H]^+^. ^b^ Confirmation of *m*/*z* values of significantly varying lipid species by scanning lipid maps.

**Table 5:** Significantly altered lipid species between the control (MDA-MB-231 cells treated with DMSO) and 2,3-DHBA-treated cells (MDA-MB-231 cells treated with the IC_50_ concentration of 2,3-DHBA for 48 h) for NIM (2,3-DHBA treatment versus control).

*m*/*z*	Lipid Classes Code ^a^	Lipid Class Name	Formula ^b^
556.0003	LMFA01030830	Fatty acid	C_38_H_67_O_2_
647.2407	LMGP06050013	Glycerophospholipid	C_31_H_52_O_12_P
	LMGP10010012	Glycerophospholipid	C_35_H_68_O_8_P
	LMGP20060025	Glycerophospholipid	C_31_H_52_O_12_P
	LMSP02010009	Sphingolipid	C_42_H_80_NO_3_
	LMSP03020059	Sphingolipid	C_34_H_68_N_2_O_7_P
683.008	LMGP10010151	Glycerophospholipid	C_38_H_68_O_8_P
	LMGP20050015	Glycerophospholipid	C_31_H_56_O_14_P
695.1657	LMGP10010025	Glycerophospholipid	C_39_H_68_O_8_P
	LMGP20050022	Glycerophospholipid	C_32_H_56_O_14_P
	LMSP02020018	Sphingolipid	C_44_H_88_NO_4_
734.0054	LMGP02010533	Glycerophospholipid	C_41_H_69_NO_8_P
	LMGP03010029	Glycerophospholipid	C_38_H_73_NO_10_P
747.5523	LMGL03012646	Glycerolipid	C_47_H_87_O_6_
	LMGP04010002	Glycerophospholipid	C_40_H_76_O_10_P
	LMGP04020011	Glycerophospholipid	C_41_H_80_O_9_P
	LMGP10010039	Glycerophospholipid	C_43_H_72_O_8_P
	LMGP15040002	Glycerophospholipid	C_32_H_60_O_17_P
748.5564	LMGP20040036	Glycerophospholipid	C_36_H_63_NO_13_P
	LMGP01010447	Glycerophospholipid	C_42_H_71_NO_8_P
750.532	LMSP03030150	Sphingolipid	C_38_H_73_NO_11_P
788.5367	LMGP01010006	Glycerophospholipid	C_44_H_87_NO_8_P
	LMGP01011461	Glycerophospholipid	C_45_H_75_NO_8_P
	LMGP01020080	Glycerophospholipid	C_45_H_91_NO_7_P
	LMGP01030015	Glycerophospholipid	C_46_H_79_NO_7_P
	LMGP03010025	Glycerophospholipid	C_42_H_79_NO_10_P
	LMGP03020033	Glycerophospholipid	C_43_H_83_NO_9_P
	LMSP05010079	Sphingolipid	C_44_H_86_NO_10_
806.2032	LMGP01010645	Glycerophospholipid	C_46_H_81_NO_8_P
	LMGP02030091	Glycerophospholipid	C_47_H_85_NO_7_P
	LMGP03010043	Glycerophospholipid	C_44_H_73_NO_10_P
	LMGP20020004	Glycerophospholipid	C_45_H_77_NO_9_P
	LMSP03030154	Sphingolipid	C_42_H_81_NO_11_P
	LMSP06020001	Sphingolipid	C_42_H_80_NO_11_S
828.5609	LMGP01010947	Glycerophospholipid	C_48_H_79_NO_8_P
	LMGP01011518	Glycerophospholipid	C_47_H_91_NO_8_P
	LMGP01020213	Glycerophospholipid	C_48_H_95_NO_7_P
	LMGP02010287	Glycerophospholipid	C_47_H_91_NO_8_P
	LMGP03010246	Glycerophospholipid	C_45_H_83_NO_10_P
	LMGP03010396	Glycerophospholipid	C_46_H_71_NO_10_P
	LMGP03020039	Glycerophospholipid	C_46_H_87_NO_9_P
	LMGP20010043	Glycerophospholipid	C_44_H_79_NO_11_P
832.5941	LMGP01010821	Glycerophospholipid	C_48_H_83_NO_8_P
	LMGP03010045	Glycerophospholipid	C_46_H_75_NO_10_P
	LMGP03010244	Glycerophospholipid	C_45_H_87_NO_10_P
	LMGP03010395	Glycerophospholipid	C_46_H_75_NO_10_P
	LMGP03020059	Glycerophospholipid	C_46_H_91_NO_9_P
	LMSP0501AB12	Sphingolipid	C_44_H_82_NO_13_
848.7064	LMGP01011785	Glycerophospholipid	C_49_H_87_NO_8_P
	LMGP01030103	Glycerophospholipid	C_50_H_91_NO_7_P
	LMGP03010479	Glycerophospholipid	C_47_H_79_NO_10_P
	LMGP03020093	Glycerophospholipid	C_48_H_83_NO_9_P
852.5666	LMGP01011058	Glycerophospholipid	C_50_H_79_NO_8_P
	LMGP01011784	Glycerophospholipid	C_49_H_91_NO_8_P
	LMGP01020252	Glycerophospholipid	C_50_H_95_NO_7_P
	LMGP03010478	Glycerophospholipid	C_47_H_83_NO_10_P
	LMGP03010840	Glycerophospholipid	C_48_H_71_NO_10_P
	LMGP03020068	Glycerophospholipid	C_48_H_87_NO_9_P
	LMGP04010556	Glycerophospholipid	C_48_H_84_O_10_P
	LMSP03030014	Sphingolipid	C_44_H_87_NO_12_P
856.699	LMGP01011752	Glycerophospholipid	C_49_H_95_NO_8_P
	LMGP01011865	Glycerophospholipid	C_50_H_83_NO_8_P
	LMGP01020241	Glycerophospholipid	C_50_H_99_NO_7_P
	LMGP03010477	Glycerophospholipid	C_47_H_87_NO_10_P
	LMGP03010619	Glycerophospholipid	C_48_H_75_NO_10_P
	LMGP03020067	Glycerophospholipid	C_48_H_91_NO_9_P
	LMSP05010046	Sphingolipid	C_49_H_94_NO_10_
885.5403	LMGP06010010	Glycerophospholipid	C_47_H_82_O_13_P
886.5437	LMGP03010786	Glycerophospholipid	C_50_H_81_NO_10_P

^a^ Volcano plot analysis; comparison of significantly changing *m*/*z* values (*p* ≤ 0.05) by comparing MDA-MB-231 breast cancer cells with DMSO or 2,3-DHBA for 48 h NIM [M+H]^−^. ^b^ Confirmation of *m*/*z* values of significantly varying lipid species by scanning lipid maps.

## Data Availability

The original contributions presented in this study are included in the article/Supplementary Material. Further inquiries can be directed to the corresponding author.

## Supplementary Materials

The following supporting information can be downloaded at: https://www.mdpi.com/article/10.3390/biom15091341/s1, Figure S1. Positive ion ESI–MS spectra of MCF-7 cells treated with DMSO (control) for 200–1200 *m*/*z*. Figure S2. Positive ion ESI–MS spectra of MCF-7 cells treated with IC_50_ concentration of 2,3-DHBA (8.61 mM) for 200–1200 *m*/*z*. Figure S3. Positive ion ESI–MS spectra of MCF-7 cells treated with DMSO (control) for 600–900 *m*/*z*. Figure S4. Positive ion ESI–MS spectra of MCF-7 cells treated with IC_50_ concentration of 2,3-DHBA (8.61 mM) for 300–600 *m*/*z*. Figure S5. Negative ion ESI–MS spectra of MCF-7 cells treated with DMSO (control) for 200–1200 *m*/*z*. Figure S6. Negative ion ESI–MS spectra of MCF-7 cells treated with IC_50_ concentration of 2,3-DHBA (8.61 mM) for 200–1200 *m*/*z*. Figure S7. Negative ion ESI–MS spectra of MCF-7 cells treated with DMSO (control) for 600–900 *m*/*z*. Figure S8. Negative ion ESI–MS spectra of MCF-7 cells treated with IC_50_ concentration of 2,3-DHBA (8.61 mM) for 300–600 *m*/*z*. Figure S9. Positive ion ESI–MS spectra of MDA-MB-231 cells treated with DMSO (control) for 200–1200 *m*/*z*. Figure S10. Positive ion ESI–MS spectra of MDA-MB-231 cells treated with IC_50_ concentration of 2,3-DHBA (8.61 mM) for 200–1200 *m*/*z*. Figure S11. Positive ion ESI–MS spectra of MDA-MB-231 cells treated with DMSO (control) for 600–900 *m*/*z*. Figure S12. Positive ion ESI–MS spectra of MDA-MB-231 cells treated with IC_50_ concentration of 2,3-DHBA (8.61 mM) for 300–600 *m*/*z*. Figure S13. Negative ion ESI–MS spectra of MDA-MB-231 cells treated with DMSO (control) for 200–1200 *m*/*z*. Figure S14. Negative ion ESI–MS spectra of MDA-MB-231 cells treated with IC_50_ concentration of 2,3-DHBA (8.61 mM) for 200–1200 *m*/*z*. Figure S15. Negative ion ESI–MS spectra of MDA-MB-231 cells treated with DMSO (control) for 600–900 *m*/*z*. Figure S16. Negative ion ESI–MS spectra of MDA-MB-231 cells treated with IC_50_ concentration of 2,3-DHBA (8.61 mM) for 300–600 *m*/*z*. Figure S17. *t*-test of MCF-7 cells treated with IC_50_ concentration of 2,3-DHBA (8.61 mM) for positive ion mode. Figure S18. Fold change of MCF-7 cells treated with IC_50_ concentration of 2,3-DHBA (8.61 mM) for positive ion mode. Figure S19. *t*-test of MCF-7 cells treated with IC_50_ concentration of 2,3-DHBA (8.61 mM) for negative ion mode. Figure S20. Fold change of MCF-7 cells treated with IC_50_ concentration of 2,3-DHBA (8.61 mM) for negative ion mode. Figure S21. *t*-test of MDA-MB-231 cells treated with IC_50_ concentration of 2,3-DHBA (5.48 mM) for positive ion mode. Figure S22. Fold change of MDA-MB-231 cells treated with IC_50_ concentration of 2,3-DHBA (5.48 mM) for positive ion mode. Figure S23. *t*-test of MDA-MB-231 cells treated with IC_50_ concentration of 2,3-DHBA (5.48 mM) for negative ion mode. Figure S24. Fold change of MDA-MB-231 cells treated with IC_50_ concentration of 2,3-DHBA (5.48 mM) for negative ion mode.

## References

[1] Juurlink B.H.J., Azouz H.J., Aldalati A.M.Z., AlTinawi B.M.H., Ganguly P. (2014). Hydroxybenzoic acid isomers and the cardiovascular system. Nutr. J..

[2] Mahdi J.G., Mahdi A.J., Mahdi A.J., Bowen I.D. (2006). The historical analysis of aspirin discovery, its relation to the willow tree and antiproliferative and anticancer potential. Cell Prolif..

[3] Robbins R.J. (2003). Phenolic acids in foods: An overview of analytical methodology. J. Agric. Food Chem..

[4] Joyeux M., Lobstein A., Anton R., Mortier F. (1995). Comparative antilipoperoxidant, antinecrotic and scavanging properties of terpenes and biflavones from Ginkgo and some flavonoids. Planta Medica.

[5] Rice-Evans C.A., Miller N.J., Paganga G. (1996). Structure-antioxidant activity relationships of flavonoids and phenolic acids. Free. Radic. Biol. Med..

[6] Dinelli G., Carretero A.S., Di Silvestro R., Marotti I., Fu S., Benedettelli S., Ghiselli L., Gutiérrez A.F. (2009). Determination of phenolic compounds in modern and old varieties of durum wheat using liquid chromatography coupled with time-of-flight mass spectrometry. J. Chromatogr. A.

[7] Bacon J.R., Rhodes M.J.C. (2000). Binding affinity of hydrolyzable tannins to parotid saliva and to proline-rich proteins derived from it. J. Agric. Food Chem..

[8] Sabally K. (2011). Lipase-Catalyzed Synthesis of Selected Phenolic Lipids in Organic Solvent Media. Ph.D. Thesis.

[9] Stamatis H., Sereti V., Kolisis F.N. (2001). Enzymatic synthesis of hydrophilic and hydrophobic derivatives of natural phenolic acids in organic media. J. Mol. Catal.—B Enzym..

[10] Türk H. (2009). Bazı Sofralık Üzüm Çeşitlerinde Farklı Dönemlerde Alınan Yapraklardaki Fenolik ve Mineral Madde Değişimlerinin Belirlenmesi. Ph.D. Thesis.

[11] Grootveld M., Halliwell B. (1988). 2,3-Dihydroxybenzoic acid is a product of human aspirin metabolism. Biochem. Pharmacol..

[12] Liu D., Su Z., Wang C., Gu M. (2009). Separation of five isomers of dihydroxybenzoic acid by high-speed counter-current chromatography with dual-rotation elution method. J. Chromatogr. Sci..

[13] Torres A.M., Mau-Lastovicka T., Rezaaiyan R. (1987). Total phenolics and high-performance liquid chromatography of phenolic acids of avocado. J. Agric. Food Chem..

[14] Zhang K., Zuo Y. (2004). GC-MS Determination of Flavonoids and Phenolic and Benzoic Acids in Human Plasma after Consumption of Cranberry Juice. J. Agric. Food Chem..

[15] Zuo Y., Wang C., Zhan J. (2002). Separation, characterization, and quantitation of benzoic and phenolic antioxidants in American cranberry fruit by GC-MS. J. Agric. Food Chem..

[16] Blatt J., Taylor S.R., Kontoghiorghes G.J. (1989). Comparison of Activity of Deferoxamine with That of Oral Iron Chelators against Human Neuroblastoma Cell Lines. Cancer Res..

[17] Graziano J.H., Miller D.R., Grady R.W., Cerami A. (1976). Inhibition of Membrane Peroxidation in Thalassaemic Erythrocytes by 2,3-Dihydroxybenzoic Acid. Br. J. Haematol..

[18] Neilands J.B. (1995). Siderophores: Structure and function of microbial iron transport compounds. J. Biol. Chem..

[19] Wenk M.R. (2005). The emerging field of lipidomics. Nat. Rev. Drug Discov..

[20] Ejsing C.S., Sampaio J.L., Surendranath V., Duchoslav E., Ekroos K., Klemm R.W., Simons K., Shevchenko A. (2009). Global analysis of the yeast lipidome by quantitative shotgun mass spectrometry. Proc. Natl. Acad. Sci. USA.

[21] Navas-Iglesias N., Carrasco-Pancorbo A., Cuadros-Rodríguez L. (2009). From lipids analysis towards lipidomics, a new challenge for the analytical chemistry of the 21st century. Part II: Analytical lipidomics. TrAC Trends Anal. Chem..

[22] Vaz F.M., Pras-Raves M., Bootsma A.H., van Kampen A.H. (2015). Principles and practice of lipidomics. J. Inherit. Metab. Dis..

[23] Zhao Y.Y., Cheng X.L., Lin R.C. (2014). Lipidomics applications for discovering biomarkers of diseases in clinical chemistry. Int. Rev. Cell Mol. Biol..

[24] Lam S.M., Shui G. (2013). Lipidomics as a principal tool for advancing biomedical research. J. Genet. Genom..

[25] Min H.K., Lim S., Chung B.C., Moon M.H. (2011). Shotgun lipidomics for candidate biomarkers of urinary phospholipids in prostate cancer. Anal. Bioanal. Chem..

[26] Santos C.R., Schulze A. (2012). Lipid metabolism in cancer. FEBS J..

[27] Taïb B., Aboussalah A.M., Moniruzzaman M., Chen S., Haughey N.J., Kim S.F., Ahima R.S. (2019). Lipid accumulation and oxidation in glioblastoma multiforme. Sci. Rep..

[28] Zhou X., Mao J., He Z., Henegar J. (2010). Lipidomics in identifying lipid biomarkers of prostate cancer. FASEB J..

[29] Zhou X., Mao J., Ai J., Deng Y., Roth M.R., Pound C., Henegar J., Welti R., Bigler S.A., Addison C.L. (2012). Identification of plasma lipid biomarkers for prostate cancer by lipidomics and bioinformatics. PLoS ONE.

[30] Kvasnička A., Najdekr L., Dobešová D., Piskláková B., Ivanovová E., Friedecký D. (2023). Clinical lipidomics in the era of the big data. Clin. Chem. Lab. Med..

[31] Yang L., Li M., Shan Y., Shen S., Bai Y., Liu H. (2016). Recent advances in lipidomics for disease research. J. Sep. Sci..

[32] Kim J., Harper A., McCormack V., Sung H., Houssami N., Morgan E., Mutebi M., Garvey G., Soerjomataram I., Fidler-Benaoudia M.M. (2025). Global patterns and trends in breast cancer incidence and mortality across 185 countries. Nat. Med..

[33] Valuate Reports (2024). Alternative Cancer Treatment—Global Market Share and Ranking, Overall Sales and Demand Forecast 2024–2030. https://reports.valuates.com/market-reports/QYRE-Auto-25P12376/global-alternative-cancer-treatment.

[34] Boon H., Brown J.B., Gavin A., Kennard A.A., Stewart M. (1999). Breast cancer survivors’ perceptions of complementary/alternative medicine (CAM): Making the decision to use or not to use. Qual. Health Res..

[35] Rana A., Samtiya M., Dhewa T., Mishra V., Aluko R.E. (2022). Health benefits of polyphenols: A concise review. J. Food Biochem..

[36] Skehan P., Storeng R., Scudiero D., Monks A., McMahon J., Vistica D., Warren J.T., Bokesch H., Kenney S., Boyd M.R. (1990). New colorimetric cytotoxicity assay for anticancer-drug screening. J. Natl. Cancer Inst..

[37] Bligh E.G., Dyer W.J. (1959). A rapid method of total lipid extraction and purification. Can. J. Biochem. Physiol..

[38] Anderson R.L., Davis S. (1982). An organic phosphorus assay which avoids the use of hazardous perchloric acid. Clin. Chim. Acta.

[39] Peterson B., Stovall K., Monian P., Franklin J.L., Cummings B.S. (2008). Alterations in phospholipid and fatty acid lipid profiles in primary neocortical cells during oxidant-induced cell injury. Chem.-Biol. Interact..

[40] Taguchi R., Hayakawa J., Takeuchi Y., Ishida M. (2000). Two-dimensional analysis of phospholipids by capillary liquid chromatography/electrospray ionization mass spectrometry. J. Mass Spectrom..

[41] Fahy E., Sud M., Cotter D., Subramaniam S. (2007). LIPID MAPS online tools for lipid research. Nucleic Acids Res..

[42] Chong J., Soufan O., Li C., Caraus I., Li S., Bourque G., Wishart D.S., Xia J. (2018). MetaboAnalyst 4.0: Towards more transparent and integrative metabolomics analysis. Nucleic Acids Res..

[43] Kinsey G.R., Blum J.L., Covington M.D., Cummings B.S., McHowat J., Schnellmann R.G. (2008). Decreased iPLA2γ expression induces lipid peroxidation and cell death and sensitizes cells to oxidant-induced apoptosis. J. Lipid Res..

[44] Zhang L., Peterson B.L., Cummings B.S. (2005). The effect of inhibition of Ca^2+^-independent phospholipase A2 on chemotherapeutic-induced death and phospholipid profiles in renal cells. Biochem. Pharmacol..

[45] Dehelean C.A., Marcovici I., Soica C., Mioc M., Coricovac D., Iurciuc S., Cretu O.M., Pinzaru I. (2021). Plant-derived anticancer compounds as new perspectives in drug discovery and alternative therapy. Molecules.

[46] Liu R.H. (2003). Health benefits of fruit and vegetables are from additive and synergistic combinations of phytochemicals. Am. J. Clin. Nutr..

[47] Dachineni R., Kumar D.R., Callegari E., Kesharwani S.S., Sankaranarayanan R., Seefeldt T., Tummala H., Bhat G.J. (2017). Salicylic acid metabolites and derivatives inhibit CDK activity: Novel insights into aspirin’s chemopreventive effects against colorectal cancer. Int. J. Oncol..

[48] Sankaranarayanan R., Valiveti C.K., Dachineni R., Kumar D.R., Lick T., Bhat G.J. (2020). Aspirin metabolites 2, 3-DHBA and 2, 5-DHBA inhibit cancer cell growth: Implications in colorectal cancer prevention. Mol. Med. Rep..

[49] Rezaei-Seresht H., Cheshomi H., Falanji F., Movahedi-Motlagh F., Hashemian M., Mireskandari E. (2019). Cytotoxic activity of caffeic acid and gallic acid against MCF-7 human breast cancer cells: An in silico and in vitro study. Avicenna J. Phytomed..

[50] Kalinowska M., Gołębiewska E., Świderski G., Męczyńska-Wielgosz S., Lewandowska H., Pietryczuk A., Cudowski A., Astel A., Świsłocka R., Samsonowicz M. (2021). Plant-derived and dietary hydroxybenzoic acids—A comprehensive study of structural, anti-/pro-oxidant, lipophilic, antimicrobial, and cytotoxic activity in MDA-MB-231 and MCF-7 cell lines. Nutrients.

[51] Huang X., Liu B., Shen S. (2025). Lipid Metabolism in Breast Cancer: From Basic Research to Clinical Application. Cancers.

[52] Cai X.-X., Zhang Z.-Z., Yang X.-X., Shen W.-R., Yuan L.-W., Ding X., Yu Y., Cai W.-Y. (2025). Unveiling the impact of lipid metabolism on triple-negative breast cancer growth and treatment options. Front. Oncol..

[53] Lupu R., Menendez J.A. (2006). Pharmacological inhibitors of fatty acid synthase (FASN)-catalyzed endogenous fatty acid biogenesis: A new family of anti-cancer agents?. Curr. Pharm. Biotechnol..

[54] Janardhan S., Srivani P., Sastry G.N. (2006). Choline kinase: An important target for cancer. Curr. Med. Chem..

[55] Danilo C., Frank P.G. (2012). Cholesterol and breast cancer development. Curr. Opin. Pharmacol..

[56] Bandu R., Mok H.J., Kim K.P. (2018). Phospholipids as cancer biomarkers: Mass spectrometry-based analysis. Mass Spectrom. Rev..

[57] Postle A.D. (2012). Lipidomics. Curr. Opin. Clin. Nutr. Metab. Care.

[58] Zhang Y., Song L., Liu N., He C., Li Z. (2014). Decreased serum levels of free fatty acids are associated with breast cancer. Clin. Chim. Acta.

[59] Dória M.L., Cotrim C.Z., Simões C., Macedo B., Domingues P., Domingues M.R., Helguero L.A. (2013). Lipidomic analysis of phospholipids from human mammary epithelial and breast cancer cell lines. J. Cell. Physiol..

[60] More T.H., Bagadi M., RoyChoudhury S., Dutta M., Uppal A., Mane A., Santra M.K., Chaudhury K., Rapole S. (2017). Comprehensive quantitative lipidomic approach to investigate serum phospholipid alterations in breast cancer. Metabolomics.

[61] Jiang N., Zhang G., Pan L., Yan C., Zhang L., Weng Y., Wang W., Chen X., Yang G. (2017). Potential plasma lipid biomarkers in early-stage breast cancer. Biotechnol. Lett..

[62] Qiu Y., Wang X., Sun Y., Jin T., Tang R., Zhou X., Xu M., Gan Y., Wang R., Luo H. (2024). ACSL4-mediated membrane phospholipid remodeling induces integrin β1 activation to facilitate triple-negative breast cancer metastasis. Cancer Res..

[63] Stoica C., Ferreira A.K., Hannan K., Bakovic M. (2022). Bilayer forming phospholipids as targets for cancer therapy. Int. J. Mol. Sci..

[64] Cheng M., Bhujwalla Z.M., Glunde K. (2016). Targeting phospholipid metabolism in cancer. Front. Oncol..

[65] Fazio A., Obeng E.O., Rusciano I., Marvi M.V., Zoli M., Mongiorgi S., Ramazzotti G., Follo M.Y., McCubrey J.A., Cocco L. (2020). Subcellular localization relevance and cancer-associated mechanisms of diacylglycerol kinases. Int. J. Mol. Sci..

[66] Mérida I., Torres-Ayuso P., Ávila-Flores A., Arranz-Nicolás J., Andrada E., Tello-Lafoz M., Liébana R., Arcos R. (2017). Diacylglycerol kinases in cancer. Adv. Biol. Regul..

[67] Sakane F., Hoshino F., Ebina M., Sakai H., Takahashi D. (2021). The roles of diacylglycerol kinase α in cancer cell proliferation and apoptosis. Cancers.

[68] Ramu D., Kim E. (2025). Exosomal lipids in cancer progression and metastasis. Cancer Med..

[69] Ward A.V., Riley D., Cosper K.E., Finlay-Schultz J., Brechbuhl H.M., Libby A.E., Hill K.B., Varshney R.R., Kabos P., Rudolph M.C. (2025). Lipid metabolic reprogramming drives triglyceride storage and variable sensitivity to FASN inhibition in endocrine-resistant breast cancer cells. Breast Cancer Res..

[70] Li R.-Z., Wang X.-R., Wang J., Xie C., Wang X.-X., Pan H.-D., Meng W.-Y., Liang T.-L., Li J.-X., Yan P.-Y. (2022). The key role of sphingolipid metabolism in cancer: New therapeutic targets, diagnostic and prognostic values, and anti-tumor immunotherapy resistance. Front. Oncol..

[71] Janneh A.H., Ogretmen B. (2022). Targeting sphingolipid metabolism as a therapeutic strategy in cancer treatment. Cancers.

[72] Pal P., Atilla-Gokcumen G.E., Frasor J. (2022). Emerging roles of ceramides in breast cancer biology and therapy. Int. J. Mol. Sci..

[73] Ohya Y., Ogiso Y., Matsuda M., Sakae H., Nishida K., Miki Y., Fox T.E., Kester M., Sakamoto W., Nabe T. (2024). Pronecroptotic therapy using ceramide nanoliposomes is effective for triple-negative breast cancer cells. Cells.

[74] Schiffmann S., Sandner J., Birod K., Wobst I., Angioni C., Ruckhäberle E., Kaufmann M., Ackermann H., Lötsch J., Schmidt H. (2009). Ceramide synthases and ceramide levels are increased in breast cancer tissue. Carcinogenesis.

