# Supplementary material for: Investigation of the Effect of 2,3-Dihydrobenzoic Acid Acid (2,3-DHBA) on the Lipid Profiles of MCF-7 and MDA-MB-231 Human Breast Cancer Cells via an Untargeted Lipidomic Approach
Source: Biomolecules. 2025 Sep 19;15(9):1341. doi: 10.3390/biom15091341 (PMC12467594; doi:10.3390/biom15091341)

Supplementary Materials

For

Investigation of Effect of 2,3 Dihydrobenzoic Acid (2,3-DHBA) on Lipid Profiles of MCF-7 and MDA-MB-231 Human Breast Cancer Cells via Untargeted Lipidomic Appproach

Büşra Daş[a], Serap Şahin[a, b, *]

[a]Sivas Cumhuriyet University, Faculty of Pharmacy, Department of Biochemistry, 58140, Sivas, Türkiye

[b]Afyonkarahisar Health Sciences University, Faculty of Pharmacy, Department of Biochemistry, 03030, Afyonkarahisar, Türkiye

[*] **Corresponding author:**

Afyonkarahisar Health Sciences University, Faculty of Pharmacy, Department of Biochemistry, 03030, Afyonkarahisar, Türkiye

E-mail: serap.sahin@afsu.edu.tr

Phone: +90 533 629 48 93

Content

Figure S1. Positive ion ESI–MS spectra of MCF-7 cells treated with DMSO (control) for 200-1200 m/z. 5

Figure S2. Positive ion ESI–MS spectra of MCF-7 cells treated with IC_{50} concentration of 2,3-DHBA (8.61 mM) for 200-1200 m/z. 7

Figure S3. Positive ion ESI–MS spectra of MCF-7 cells treated with DMSO (control) for 600-900 m/z 9

Figure S4. Positive ion ESI–MS spectra of MCF-7 cells treated with IC_{50} concentration of 2,3-DHBA (8.61 mM) for 300-600 m/z. 11

Figure S5. Negative ion ESI–MS spectra of MCF-7 cells treated with DMSO (control) for 200-1200 m/z. 13

Figure S6. Negative ion ESI–MS spectra of MCF-7 cells treated with IC_{50} concentration of 2,3-DHBA (8.61 mM) for 200-1200 m/z. 15

Figure S7. Negative ion ESI–MS spectra of MCF-7 cells treated with DMSO (control) for 600-900 m/z 17

Figure S8. Negative ion ESI–MS spectra of MCF-7 cells treated with IC_{50} concentration of 2,3-DHBA (8.61 mM) for 300-600 m/z. 19

Figure S9. Positive ion ESI–MS spectra of MDA-MB-231 cells treated with DMSO (control) for 200-1200 m/z. 21

Figure S10. Positive ion ESI–MS spectra of MDA-MB-231 cells treated with IC_{50} concentration of 2,3-DHBA (8.61 mM) for 200-1200 m/z. 23

Figure S11. Positive ion ESI–MS spectra of MDA-MB-231 cells treated with DMSO (control) for 600-900 m/z 25

Figure S12. Positive ion ESI–MS spectra of MDA-MB-231 cells treated with IC_{50} concentration of 2,3-DHBA (8.61 mM) for 300-600 m/z. 27

Figure S13. Negative ion ESI–MS spectra of MDA-MB-231 cells treated with DMSO (control) for 200-1200 m/z. 29

Figure S14. Negative ion ESI–MS spectra of MDA-MB-231 cells treated with IC_{50} concentration of 2,3-DHBA (8.61 mM) for 200-1200 m/z. 31

Figure S15. Negative ion ESI–MS spectra of MDA-MB-231 cells treated with DMSO (control) for 600-900 m/z 33

Figure S16. Negative ion ESI–MS spectra of MDA-MB-231 cells treated with IC_{50} concentration of 2,3-DHBA (8.61 mM) for 300-600 m/z 35

Figure S17. t-test of MCF-7 cells treated with IC_{50} concentration of 2,3-DHBA (8.61 mM) for positive ion mode 37

Figure S18. Fold change of MCF-7 cells treated with IC_{50} concentration of 2,3-DHBA (8.61 mM) for positive ion mode 37

Figure S19. t-test of MCF-7 cells treated with IC_{50} concentration of 2,3-DHBA (8.61 mM) for negative ion mode 38

Figure S20. Fold change of MCF-7 cells treated with IC_{50} concentration of 2,3-DHBA (8.61 mM) for negative ion mode 38

Figure S21. t-test of MDA-MB-231 cells treated with IC_{50} concentration of 2,3-DHBA (5.48 mM) for positive ion mode 39

Figure S22. Fold change of MDA-MB-231 cells treated with IC_{50} concentration of 2,3-DHBA (5.48 mM) for positive ion mode 39

Figure S23. t-test of MDA-MB-231 cells treated with IC_{50} concentration of 2,3-DHBA (5.48 mM) for negative ion mode 40

Figure S24. Fold change of MDA-MB-231 cells treated with IC_{50} concentration of 2,3-DHBA (5.48 mM) for negative ion mode 40

Figure S1. Positive ion ESI–MS spectra of MCF-7 cells treated with DMSO (control) for 200-1200 m/z.

MCF-7 / Sample 1

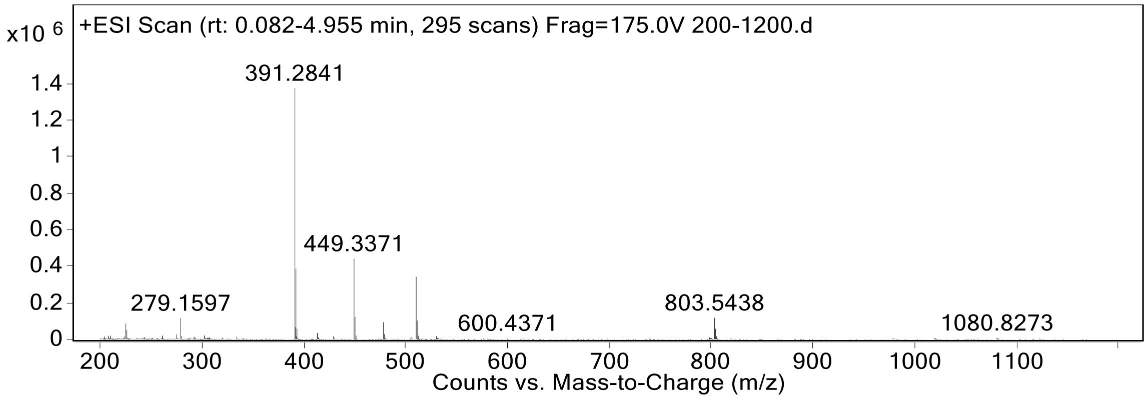

MCF-7 / Sample 2

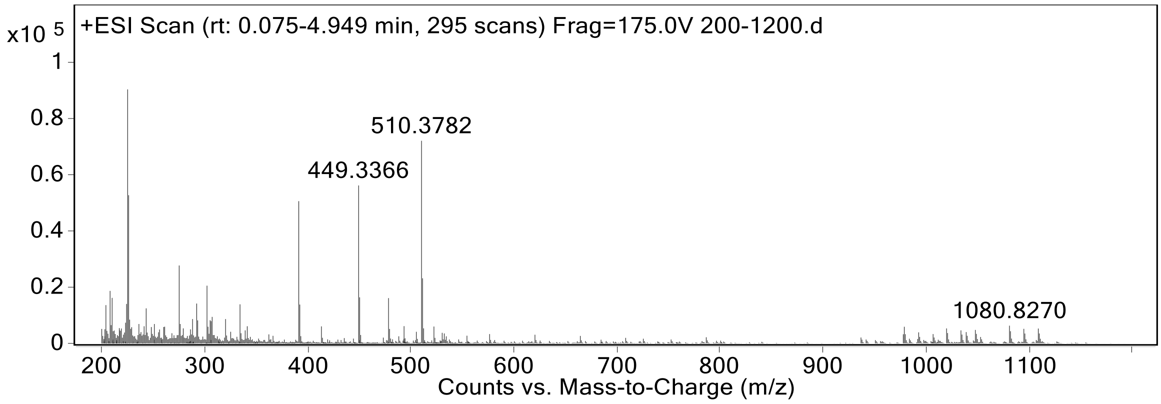

MCF-7 / Sample 3

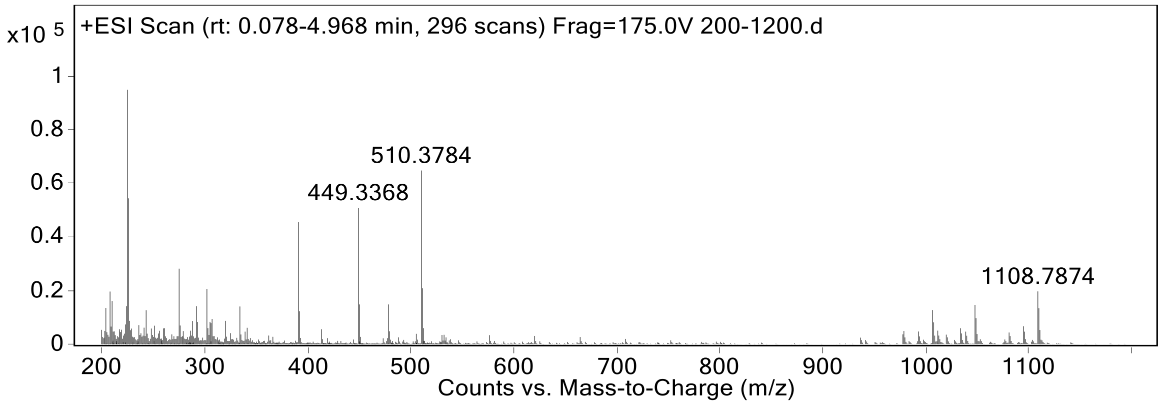

MCF-7 / Sample 4

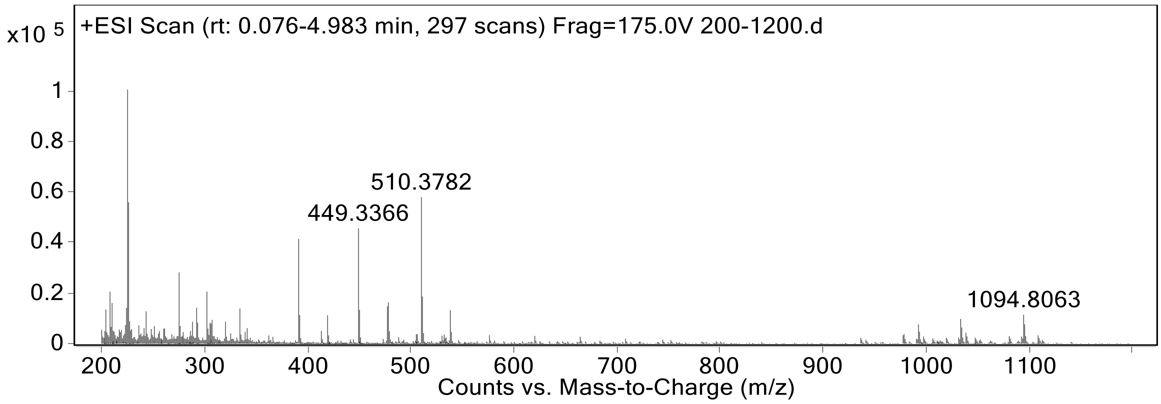

MCF-7 / Sample 5

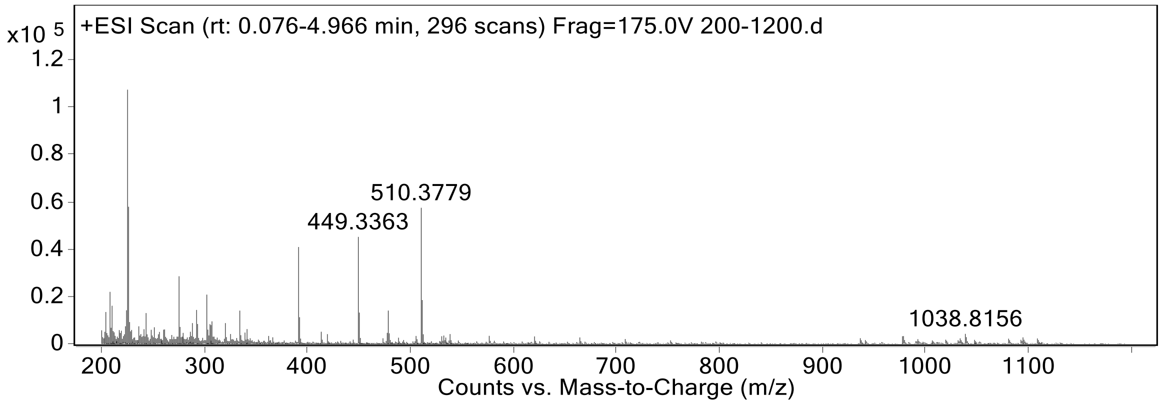

MCF-7 / Sample 6

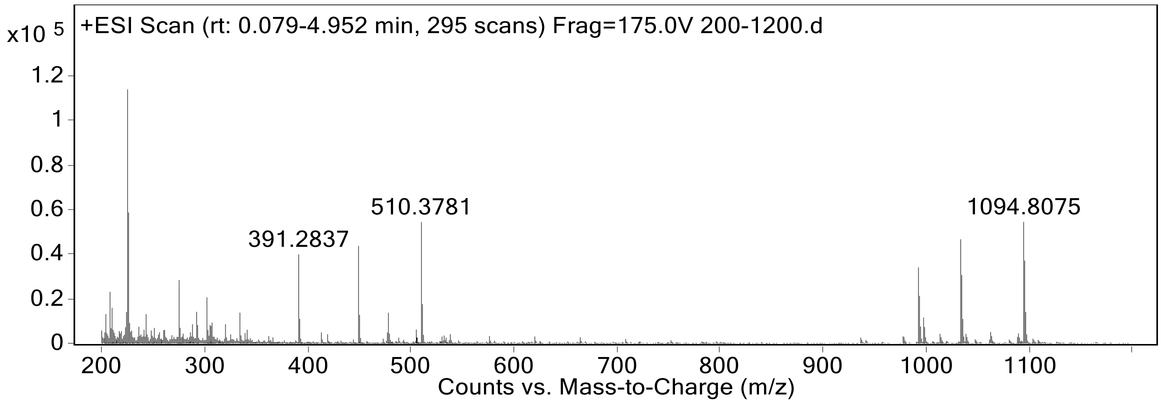

Figure S2. Positive ion ESI–MS spectra of MCF-7 cells treated with IC_{50} concentration of 2,3-DHBA (8.61 mM) for 200-1200 m/z.

MCF-7 + 2,3-DHBA / Sample 1

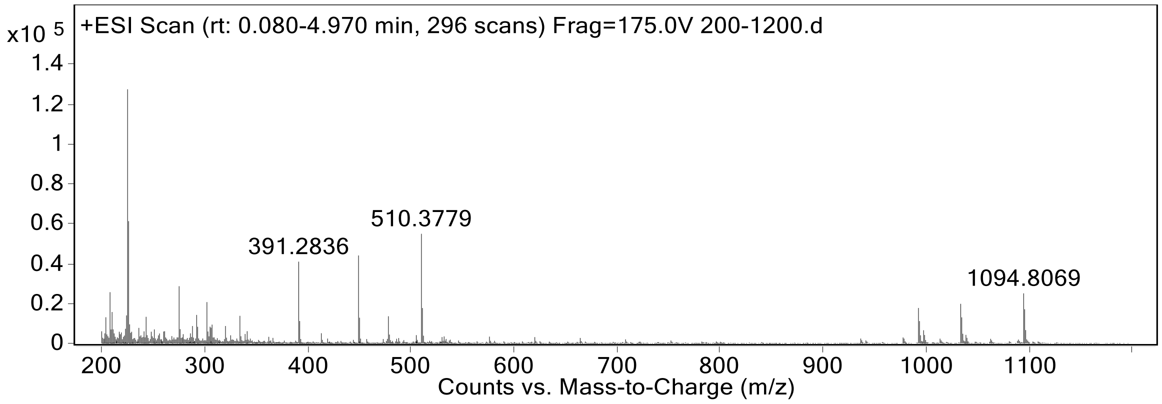

MCF-7 + 2,3-DHBA / Sample 2

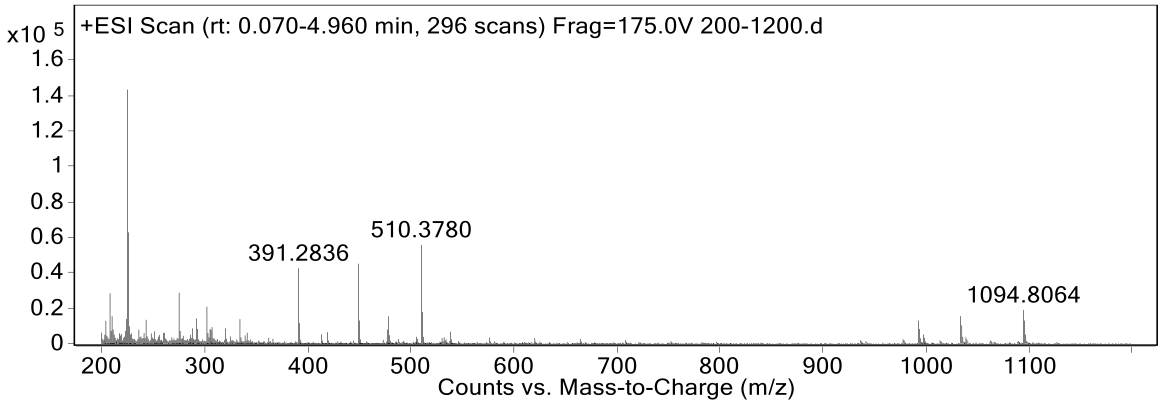

MCF-7 + 2,3-DHBA / Sample 3

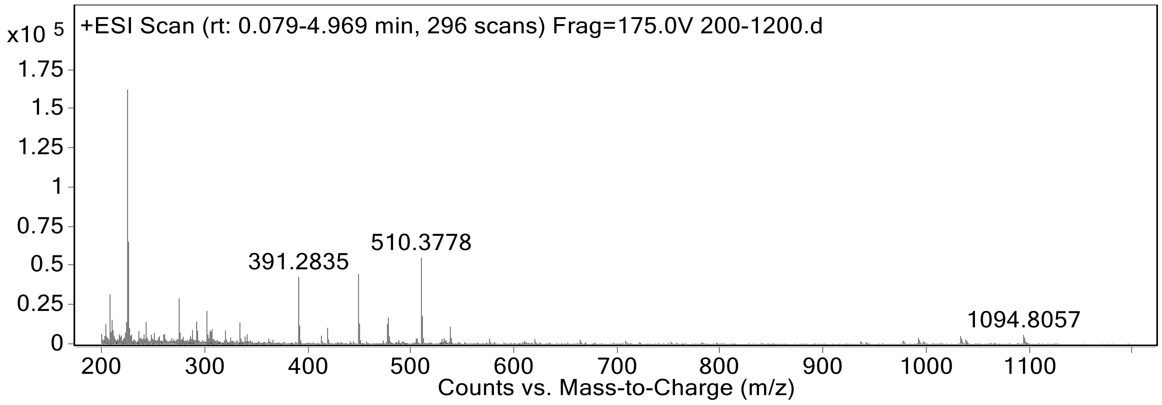

MCF-7 + 2,3-DHBA / Sample 4

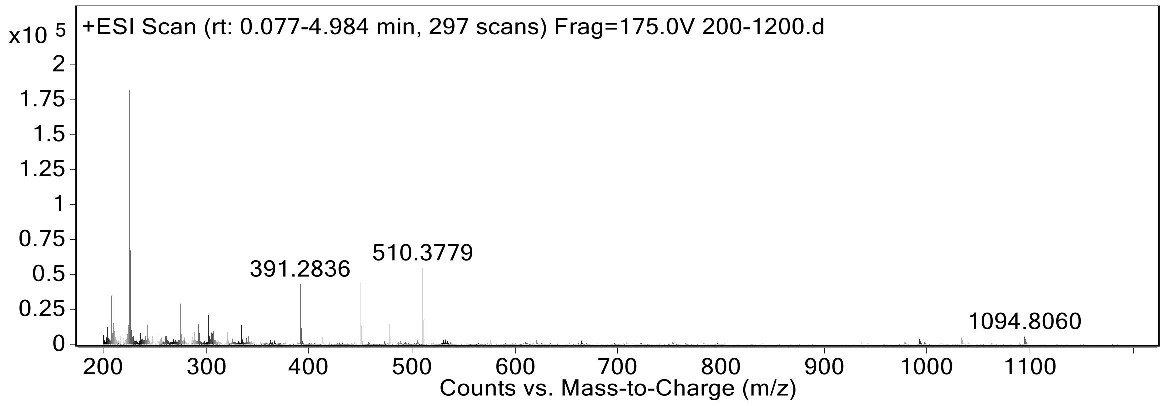

MCF-7 + 2,3-DHBA / Sample 5

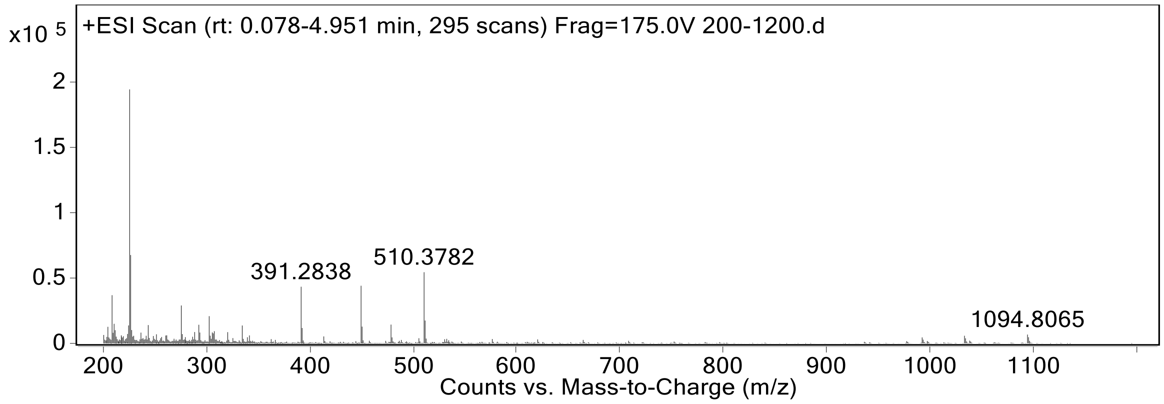

MCF-7 + 2,3-DHBA / Sample 6

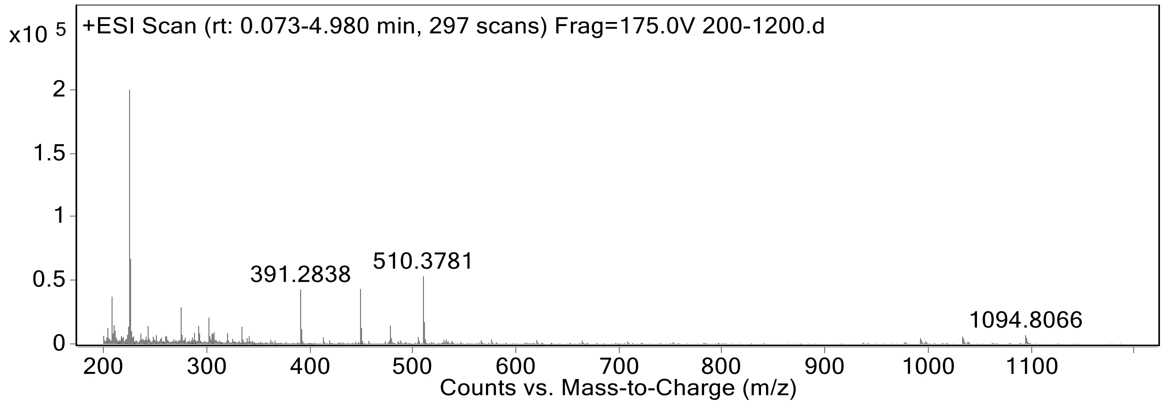

Figure S3. Positive ion ESI–MS spectra of MCF-7 cells treated with DMSO (control) for 600-900 m/z

MCF-7 /Sample 1

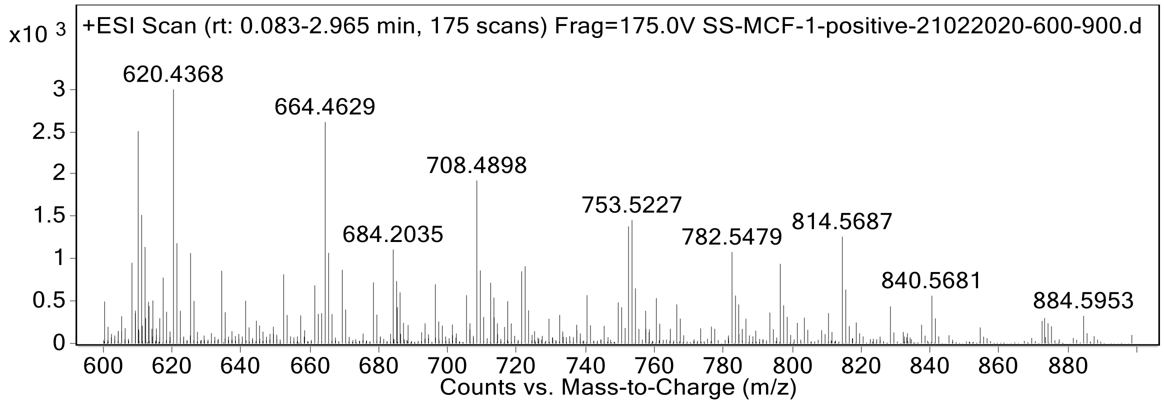

MCF-7 / Sample 2

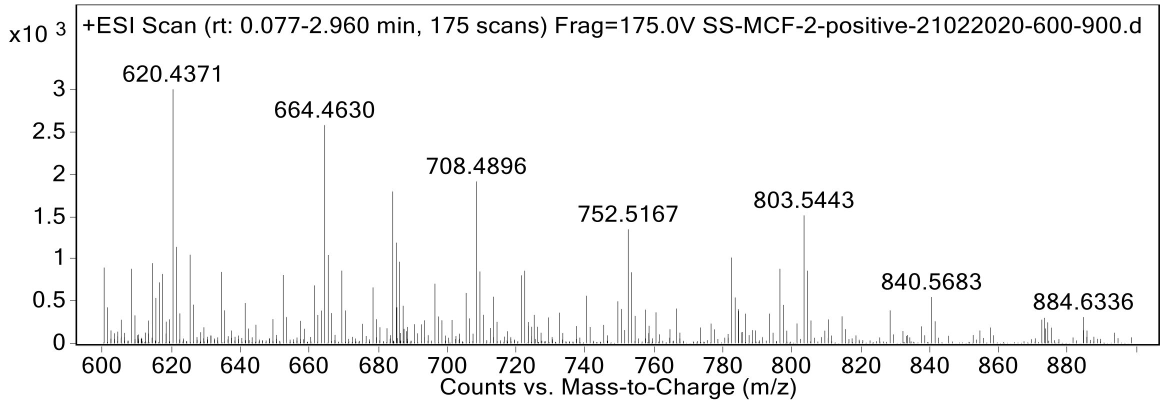

MCF-7 / Sample 3

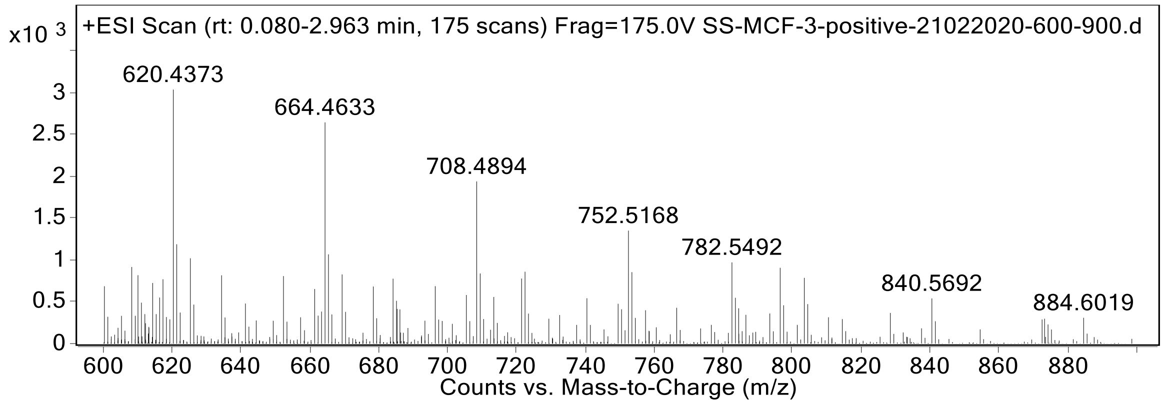

MCF-7 / Sample 4

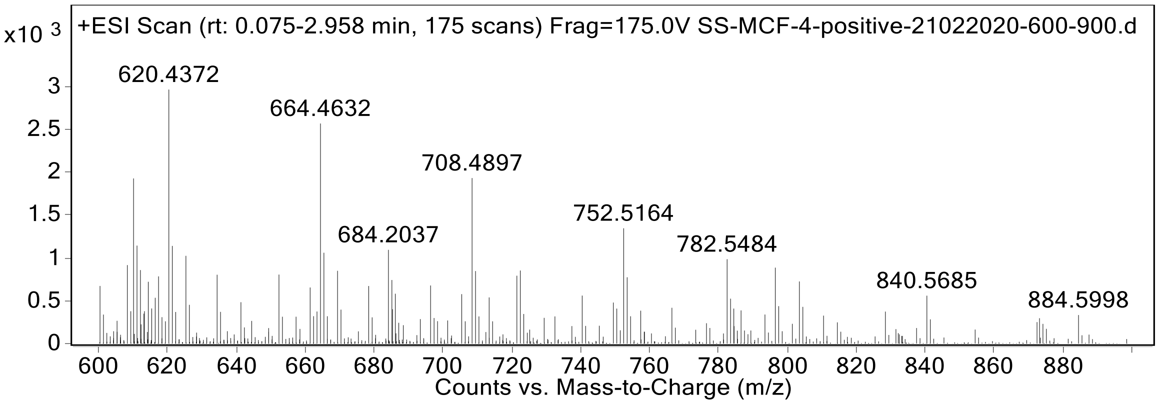

MCF-7 / Sample 5

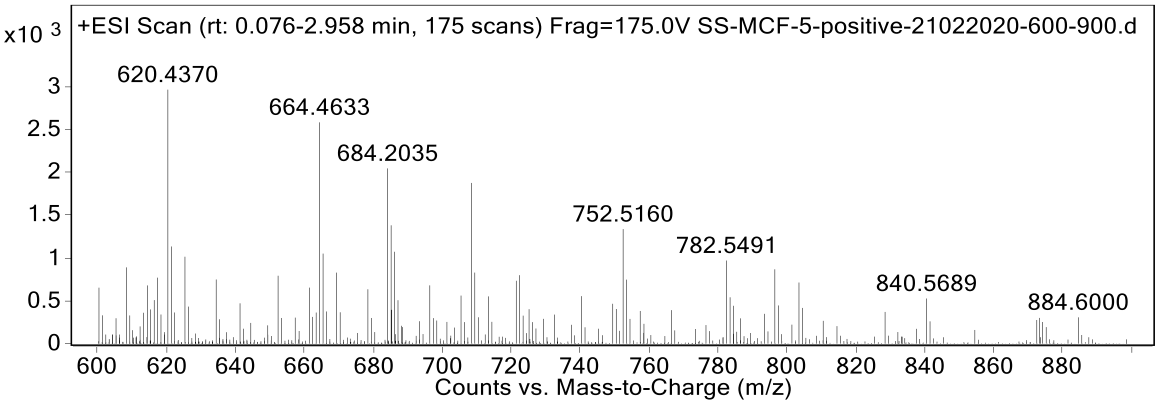

MCF-7 / Sample 6

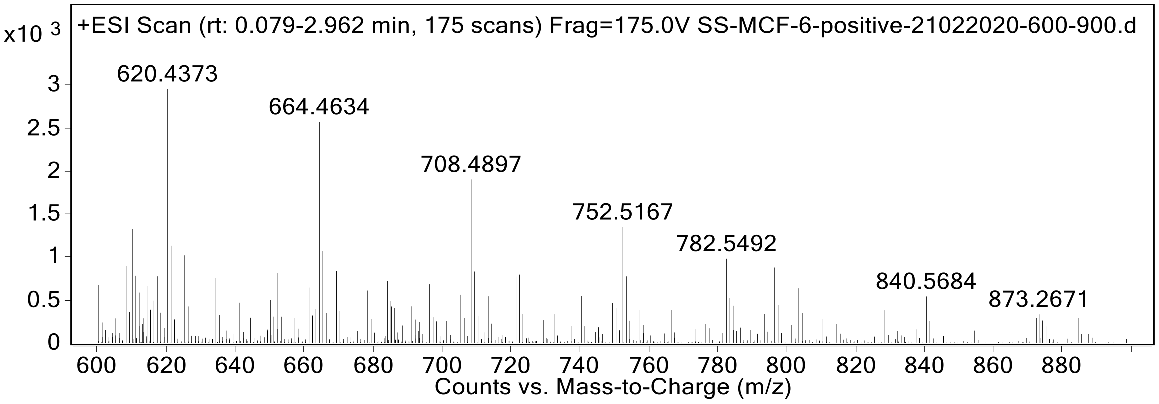

Figure S4. Positive ion ESI–MS spectra of MCF-7 cells treated with IC_{50} concentration of 2,3-DHBA (8.61 mM) for 300-600 m/z.

MCF-7 + 2,3-DHBA / Sample 1

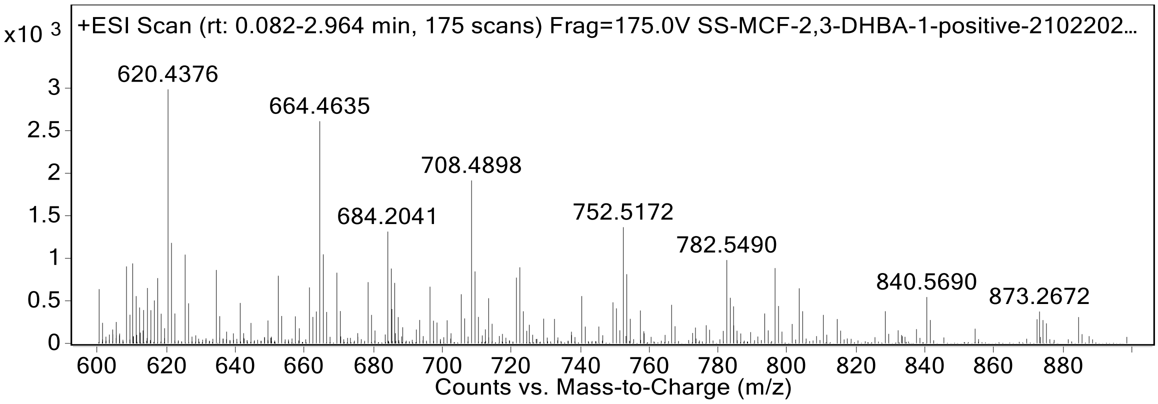

MCF-7 + 2,3-DHBA / Sample 2

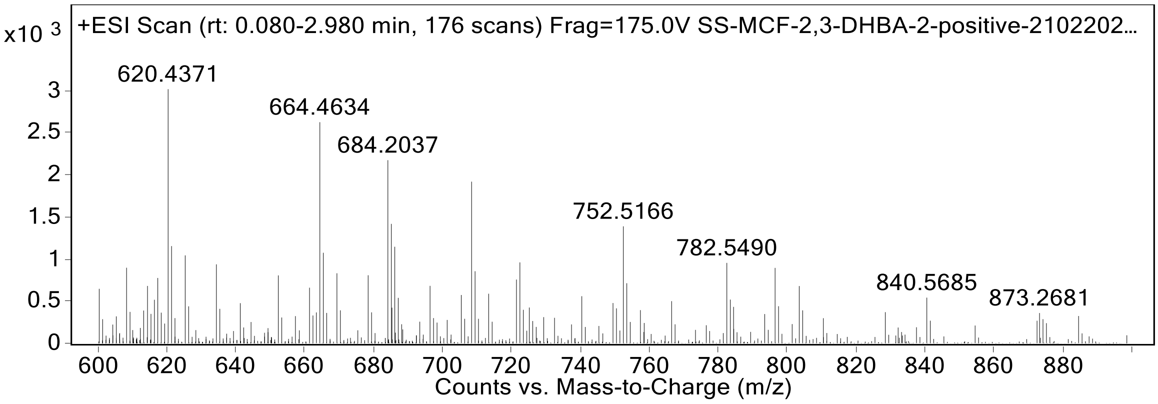

MCF-7 + 2,3-DHBA / Sample 3

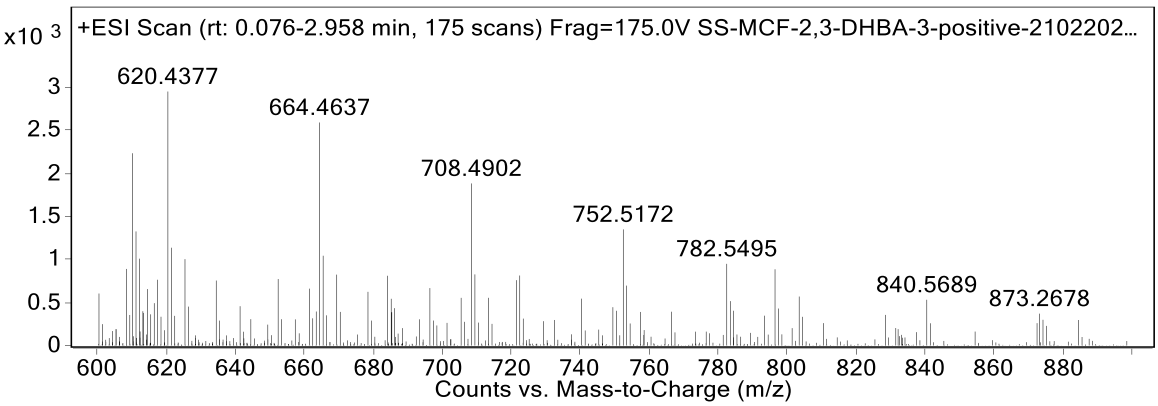

MCF-7 + 2,3-DHBA / Sample 4

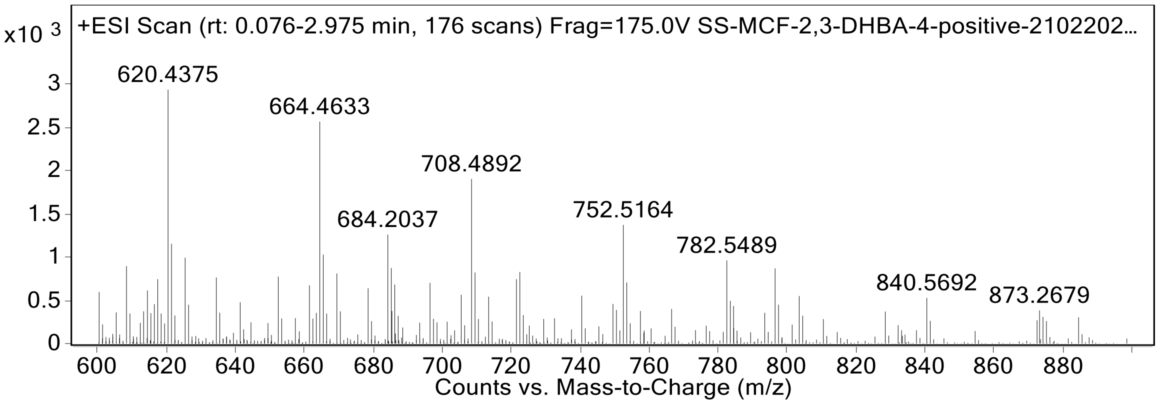

MCF-7 + 2,3-DHBA / Sample 5

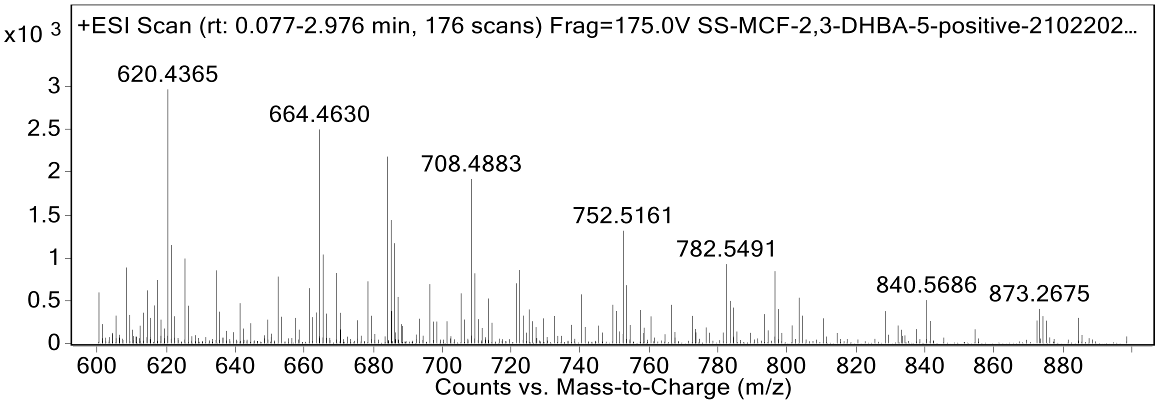

MCF-7 + 2,3-DHBA / Sample 6

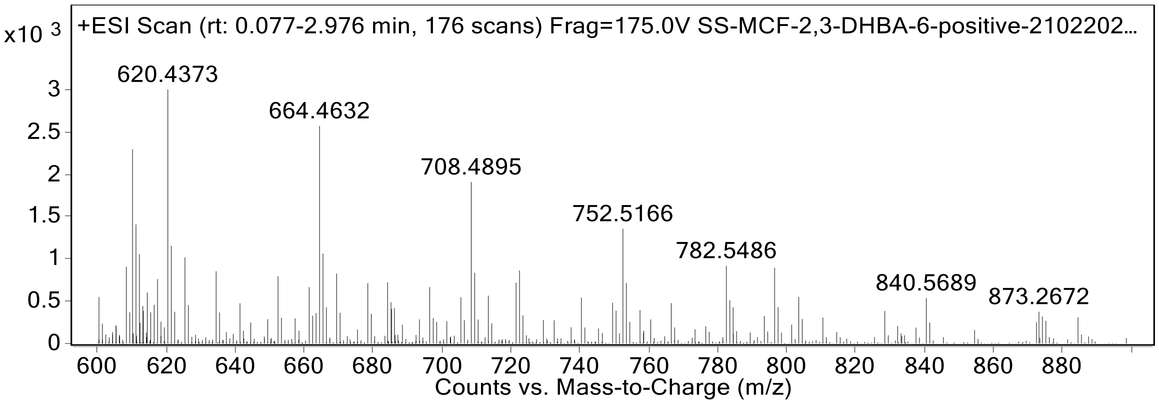

Figure S5. Negative ion ESI–MS spectra of MCF-7 cells treated with DMSO (control) for 200-1200 m/z.

MCF-7 / Sample 1

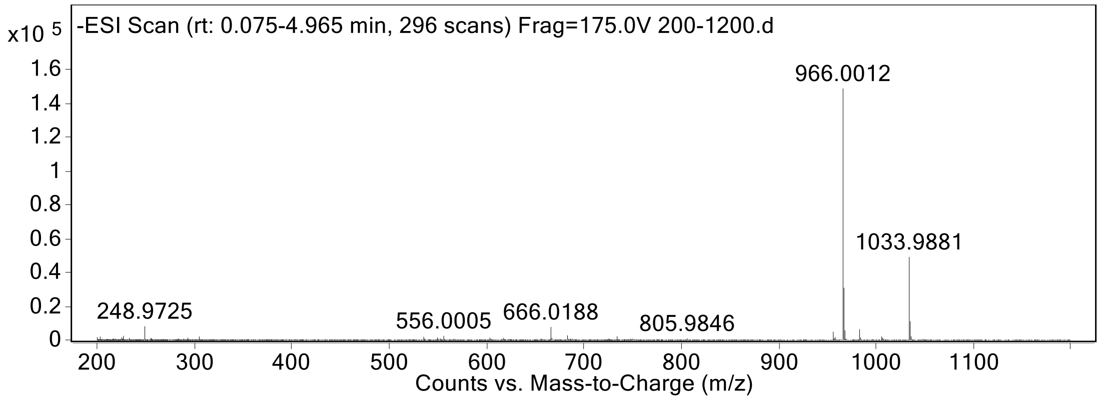

MCF-7 / Sample 2

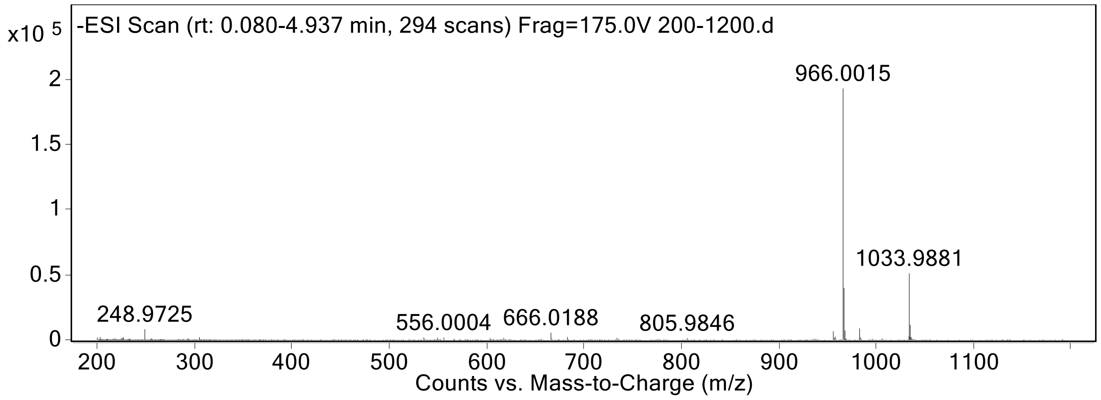

MCF-7 / Sample 3

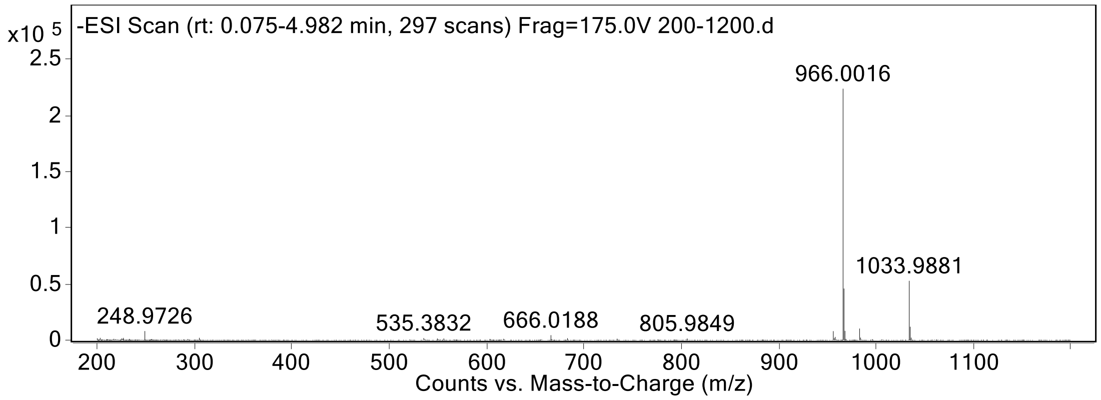

MCF-7 / Sample 4

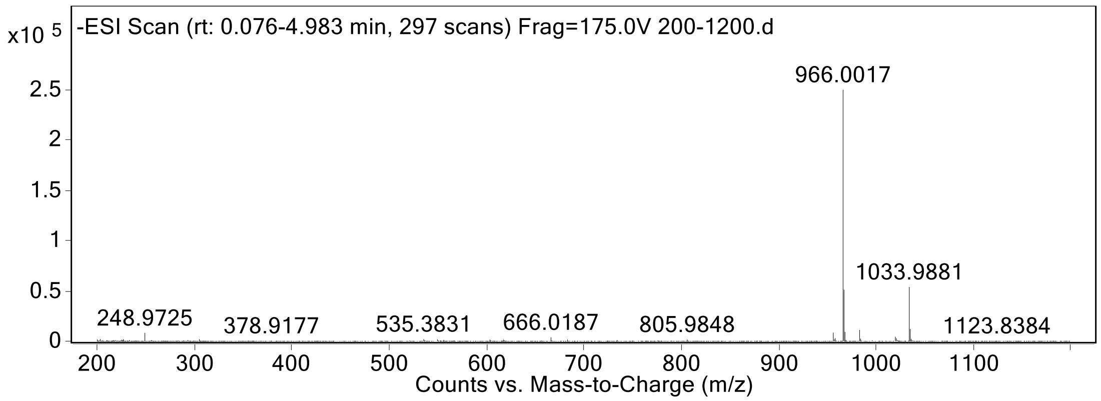

MCF-7 / Sample 5

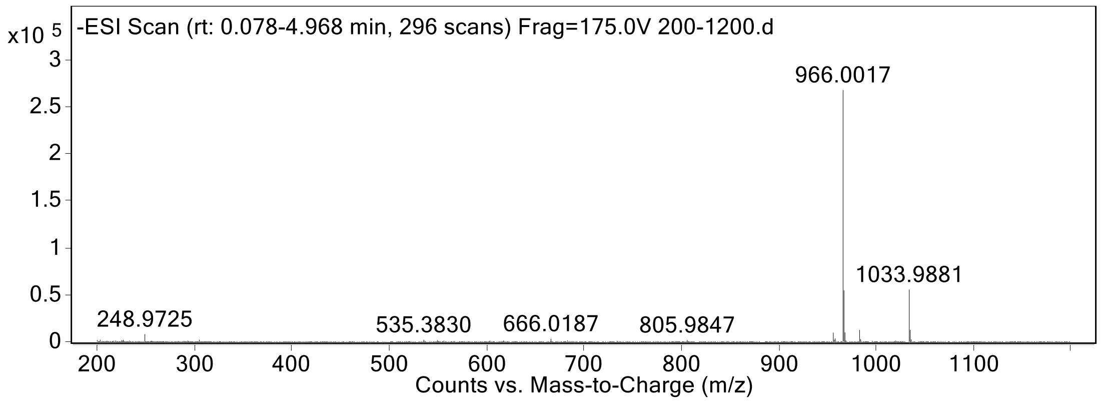

MCF-7 / Sample 6

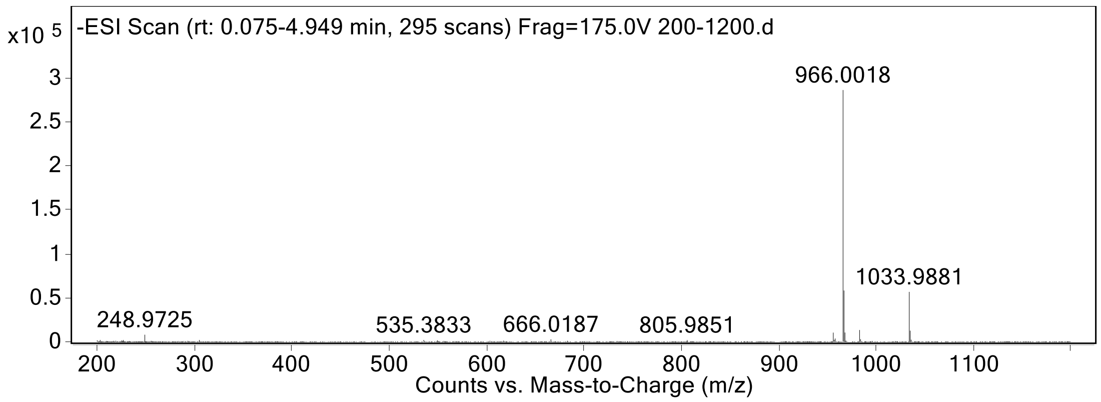

Figure S6. Negative ion ESI–MS spectra of MCF-7 cells treated with IC_{50} concentration of 2,3-DHBA (8.61 mM) for 200-1200 m/z.

MCF-7 + 2,3-DHBA / Sample 1

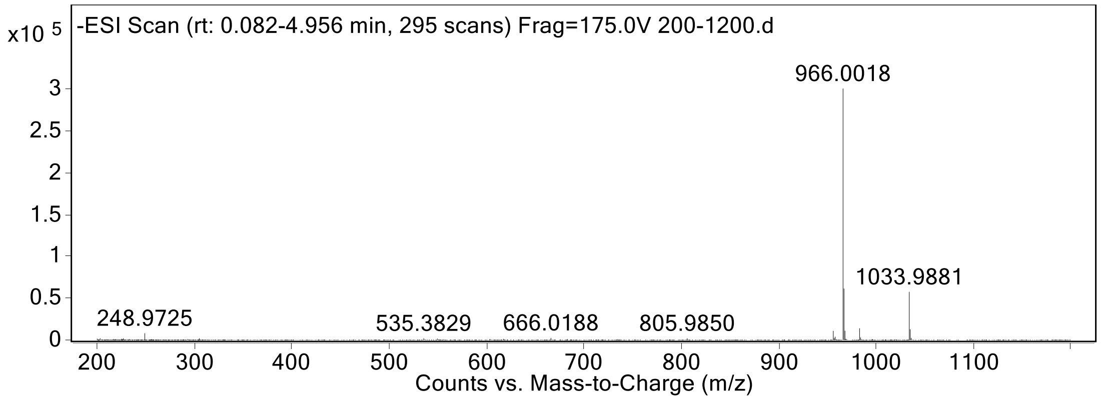

MCF-7 + 2,3-DHBA / Sample 2

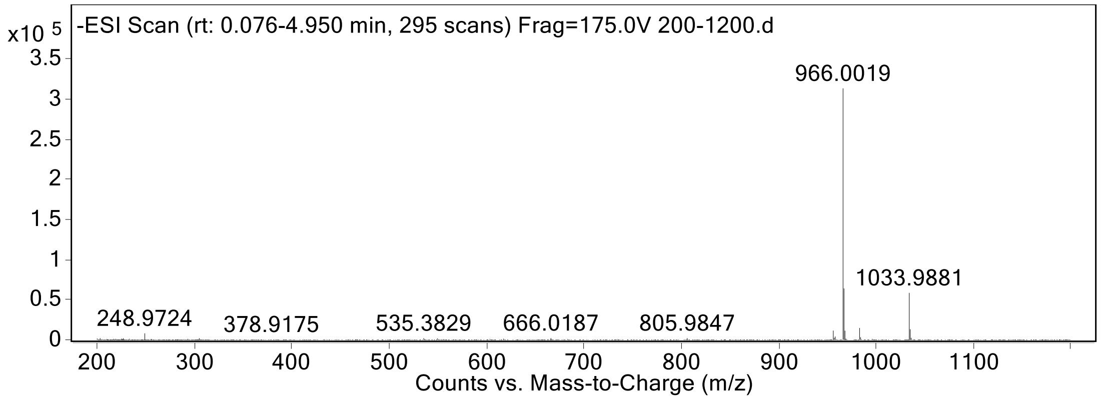

MCF-7 + 2,3-DHBA / Sample 3

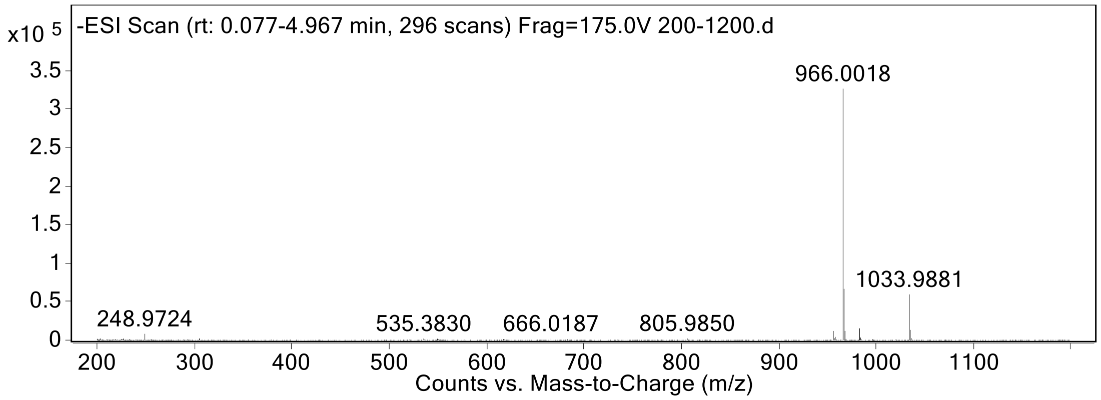

MCF-7 + 2,3-DHBA / Sample 4

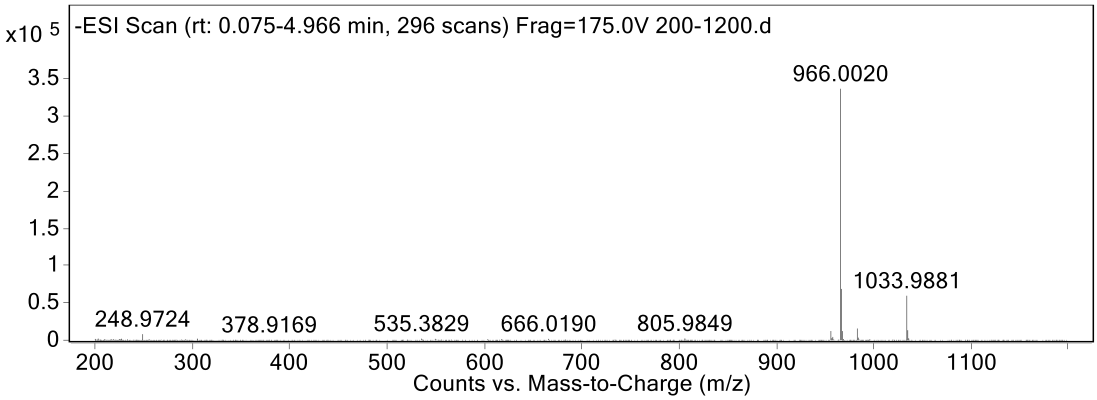

MCF-7 + 2,3-DHBA / Sample 5

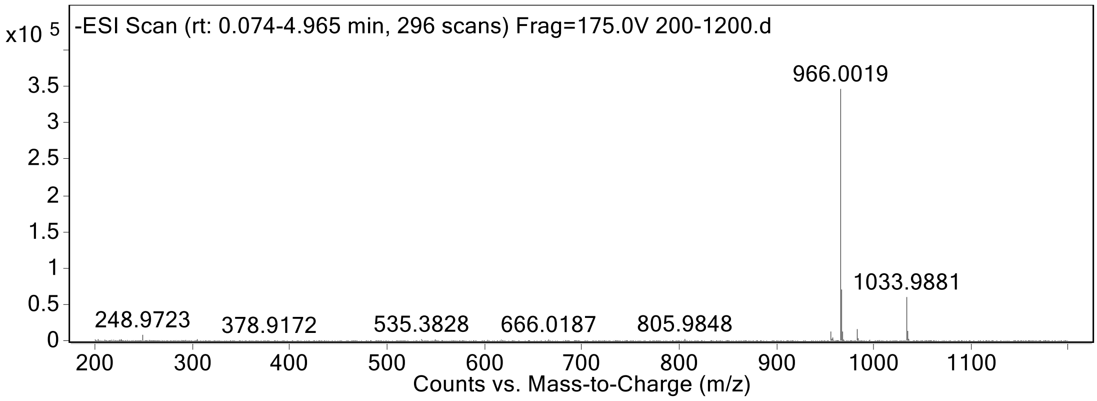

MCF-7 + 2,3-DHBA / Sample 6

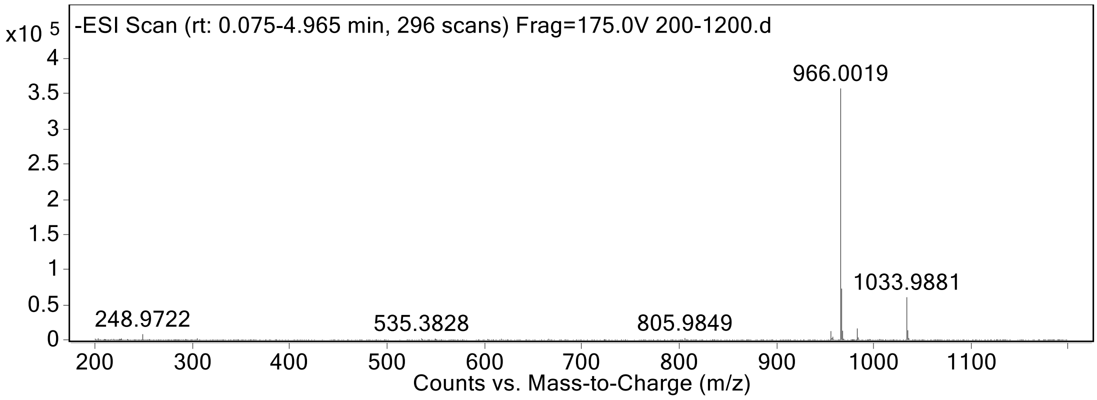

Figure S7. Negative ion ESI–MS spectra of MCF-7 cells treated with DMSO (control) for 600-900 m/z

MCF-7 /Sample 1

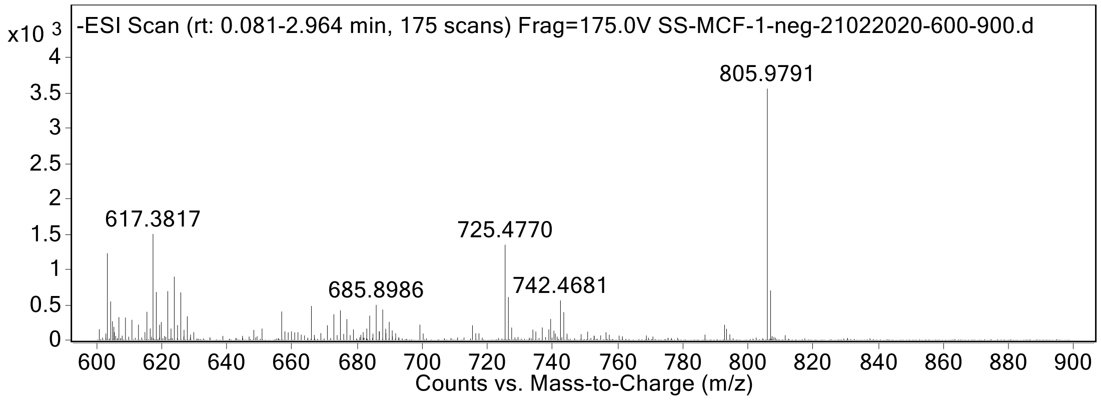

MCF-7 / Sample 2

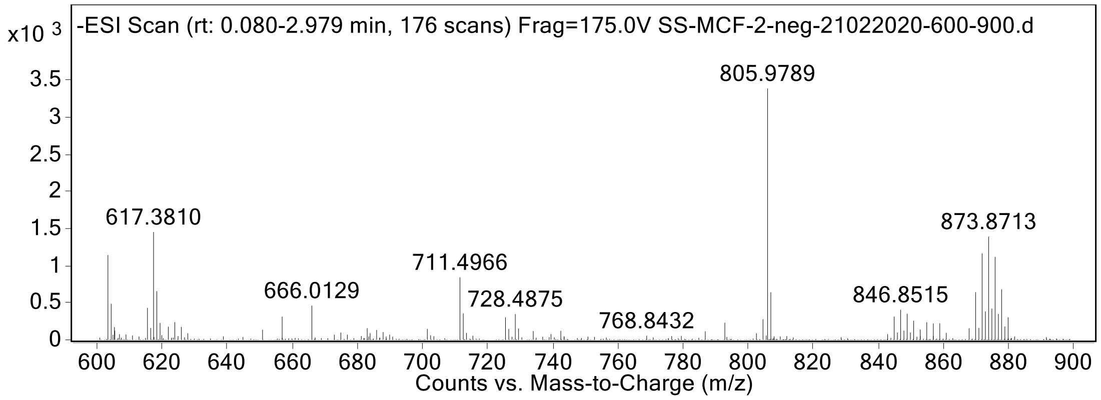

MCF-7 / Sample 3

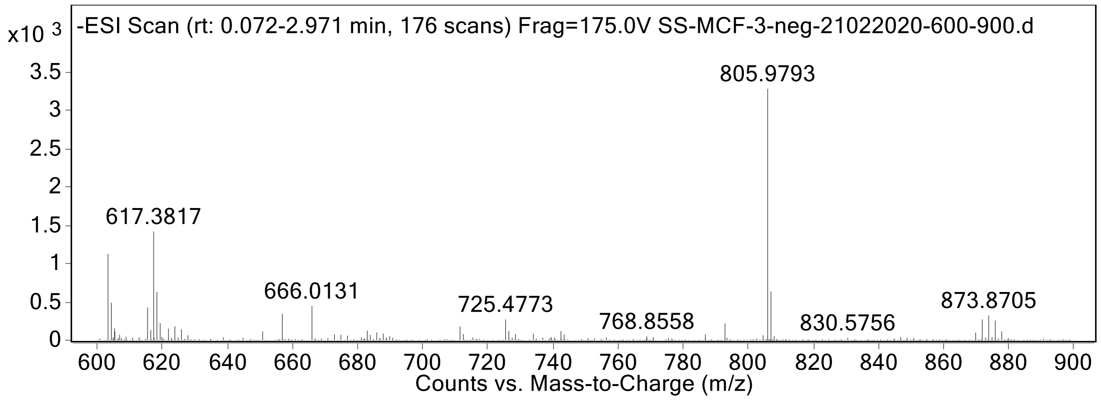

MCF-7 / Sample 4

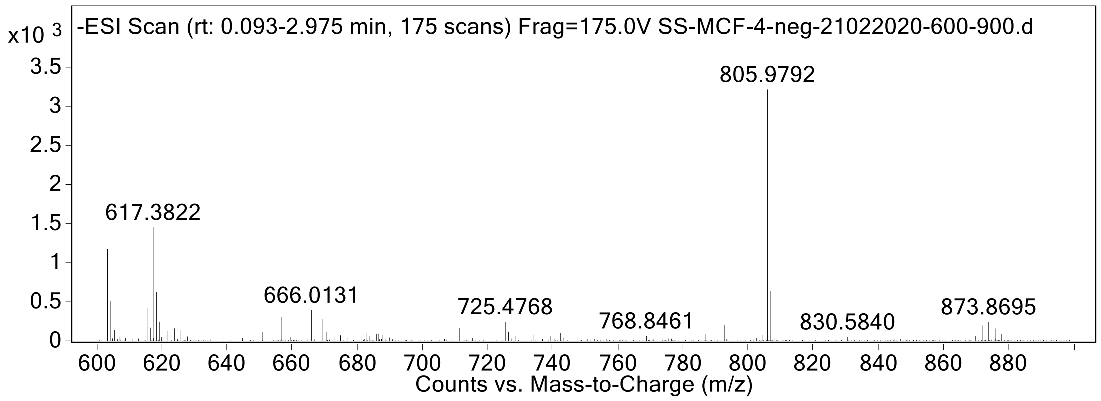

MCF-7 / Sample 5

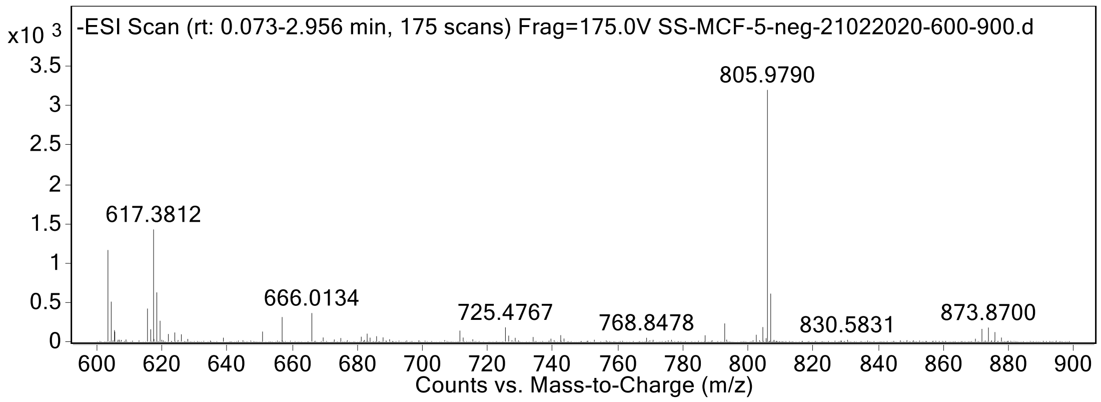

MCF-7 / Sample 6

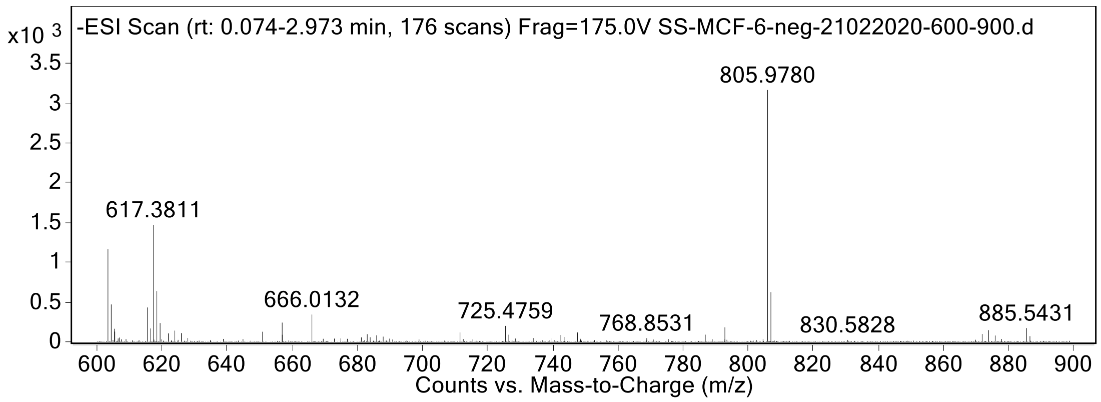

Figure S8. Negative ion ESI–MS spectra of MCF-7 cells treated with IC_{50} concentration of 2,3-DHBA (8.61 mM) for 300-600 m/z.

MCF-7 + 2,3-DHBA / Sample 1

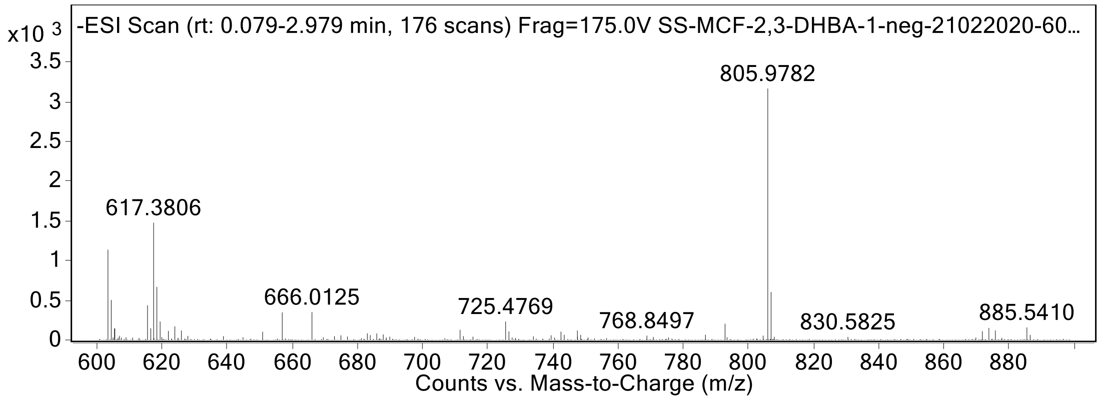

MCF-7 + 2,3-DHBA / Sample 2

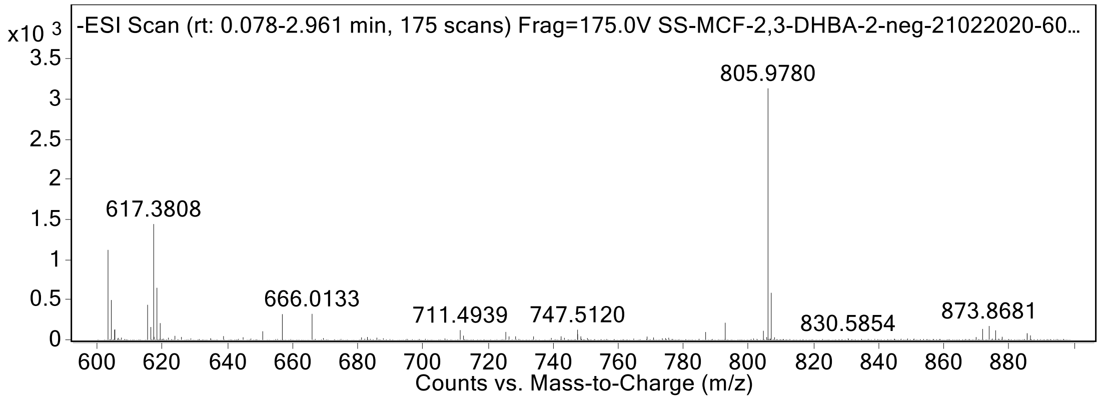

MCF-7 + 2,3-DHBA / Sample 3

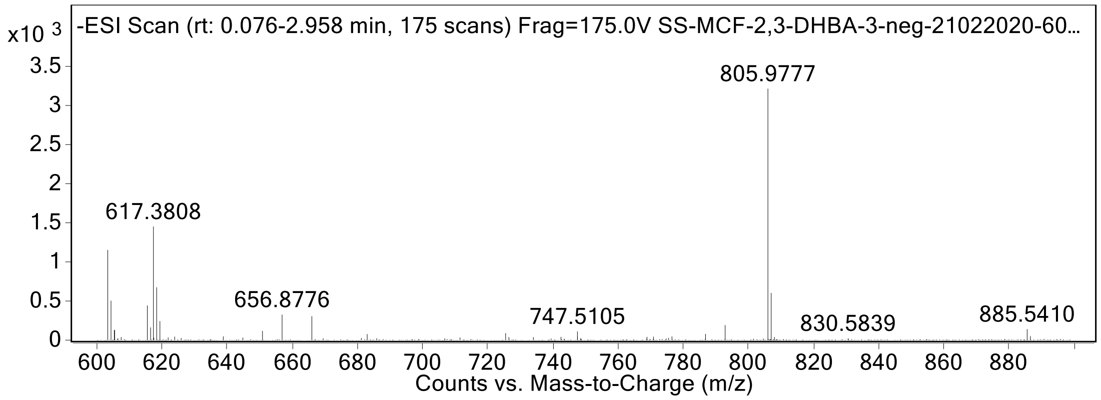

MCF-7 + 2,3-DHBA / Sample 4

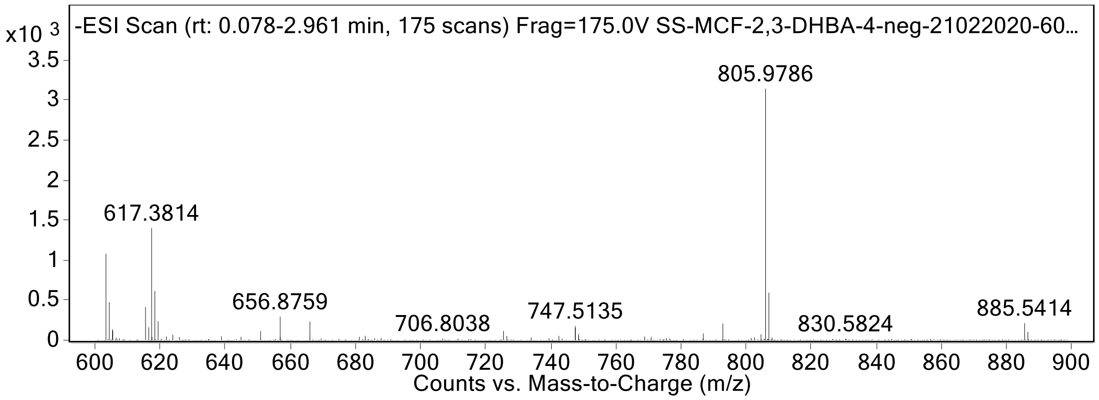

MCF-7 + 2,3-DHBA / Sample 5

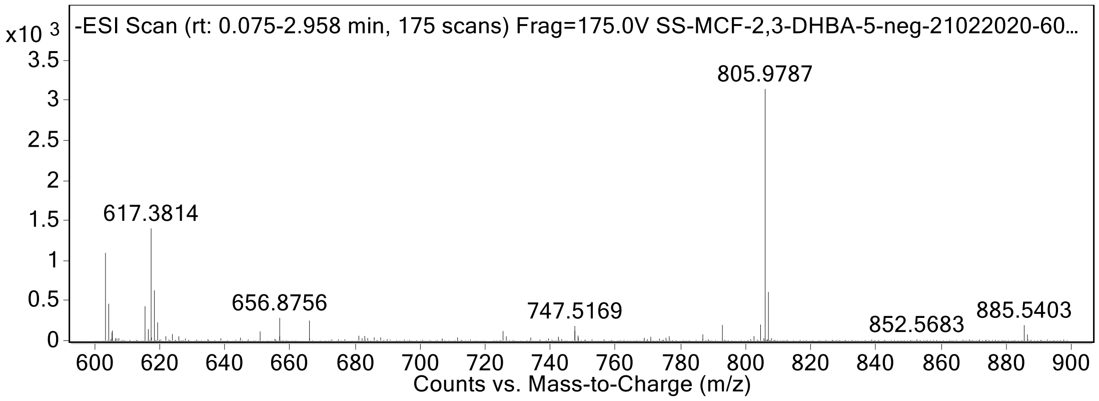

MCF-7 + 2,3-DHBA / Sample 6

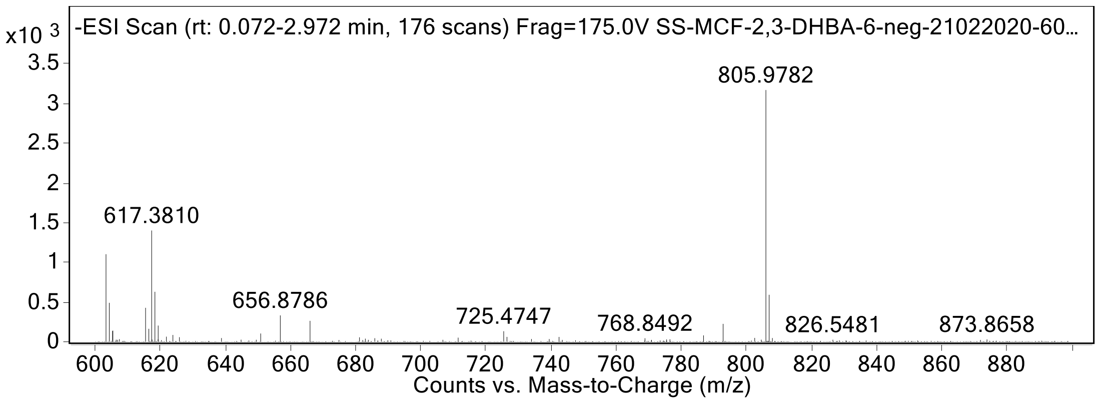

Figure S9. Positive ion ESI–MS spectra of MDA-MB-231 cells treated with DMSO (control) for 200-1200 m/z.

MDA-MB-231 / Sample 1

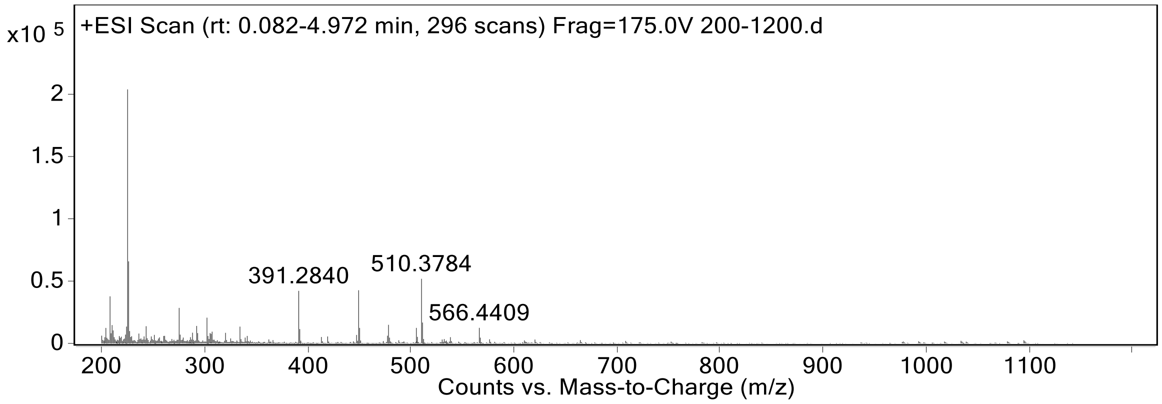

MDA-MB-231 / Sample 2

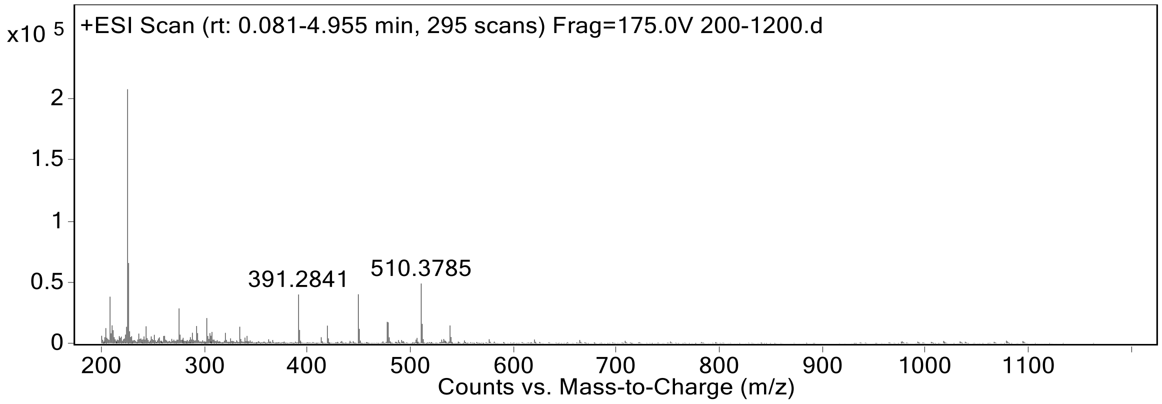

MDA-MB-231 / Sample 3

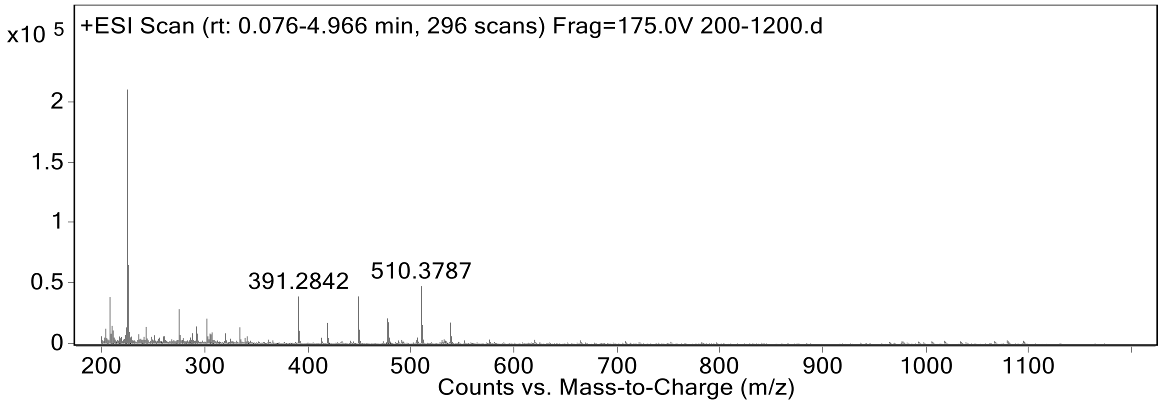

MDA-MB-231 / Sample 4

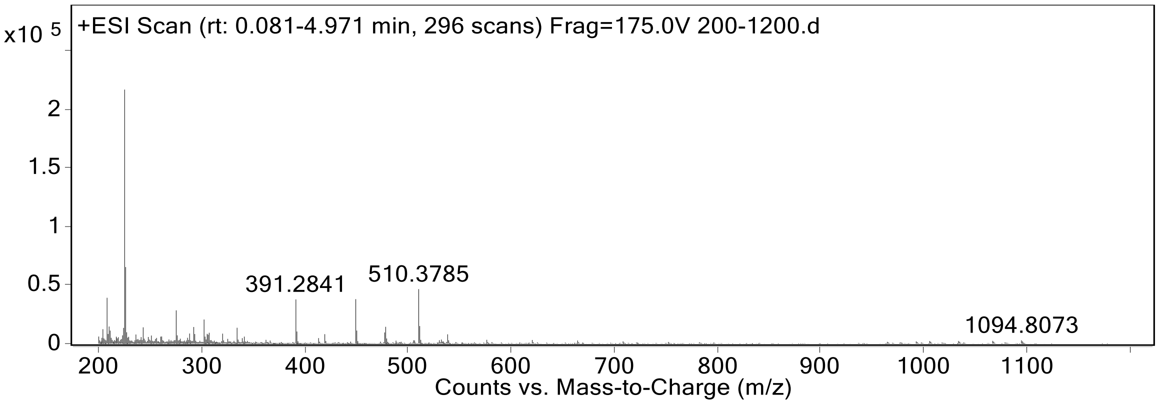

MDA-MB-231 / Sample 5

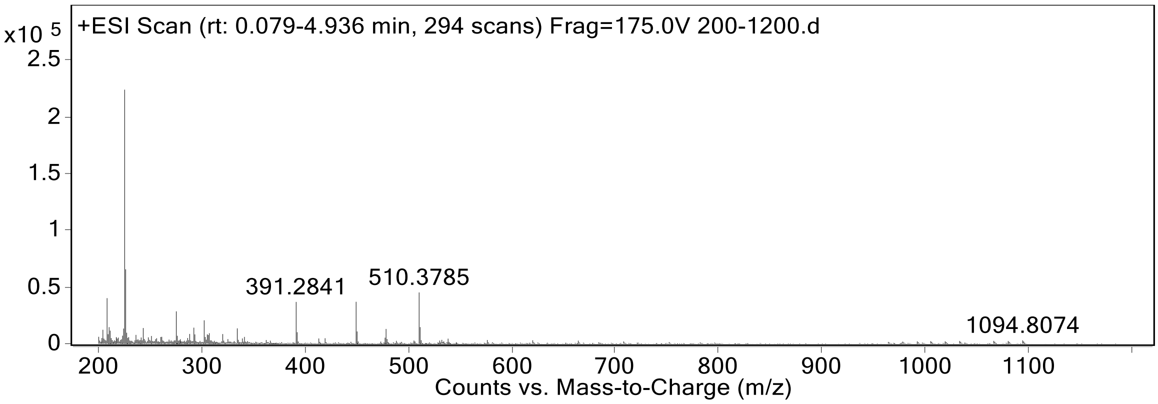

MDA-MB-231 / Sample 6

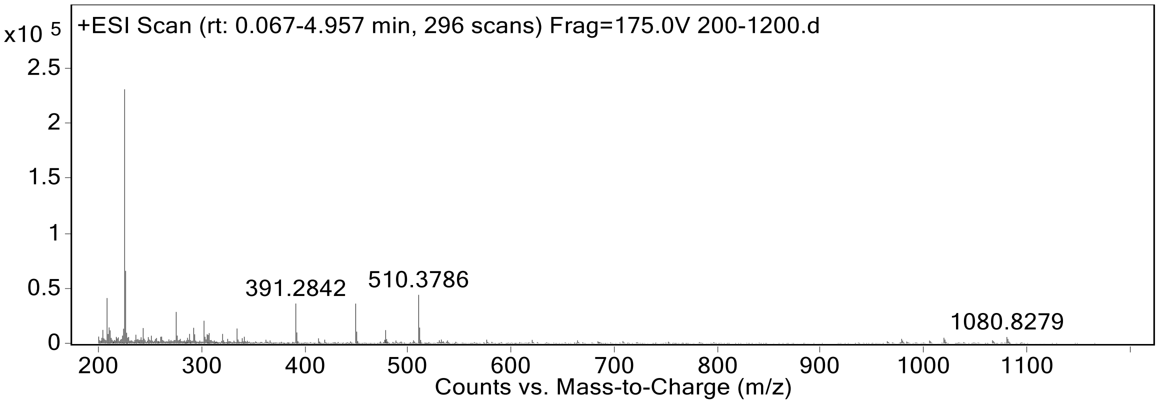

Figure S10. Positive ion ESI–MS spectra of MDA-MB-231 cells treated with IC_{50} concentration of 2,3-DHBA (8.61 mM) for 200-1200 m/z.

MDA-MB-231 + 2,3-DHBA / Sample 1

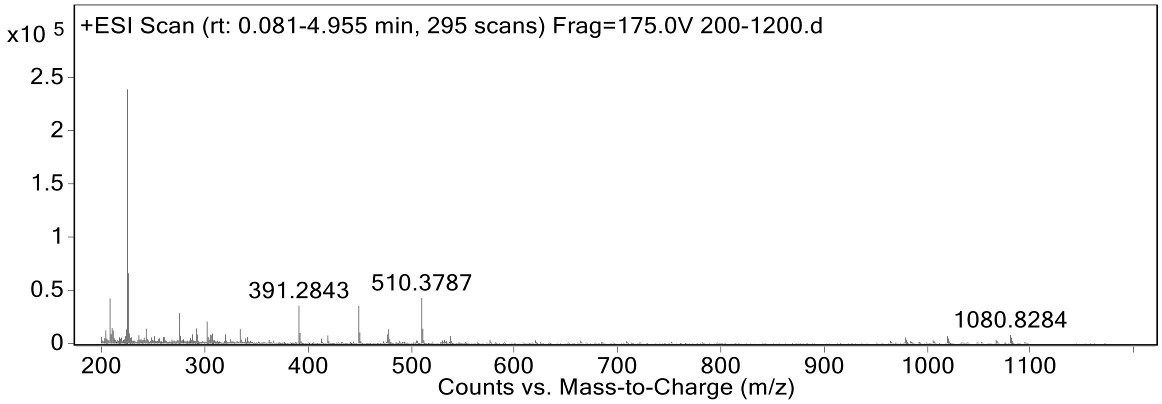

MDA-MB-231 + 2,3-DHBA / Sample 2

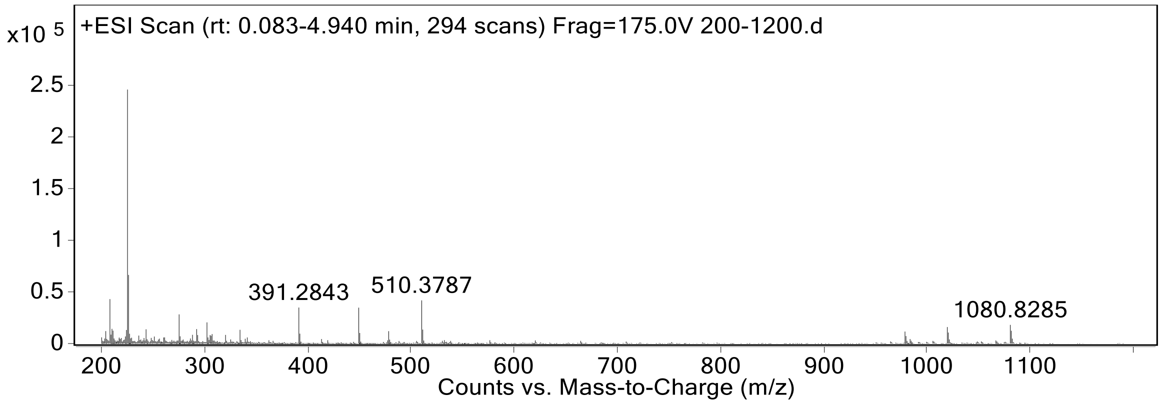

MDA-MB-231 + 2,3-DHBA / Sample 3

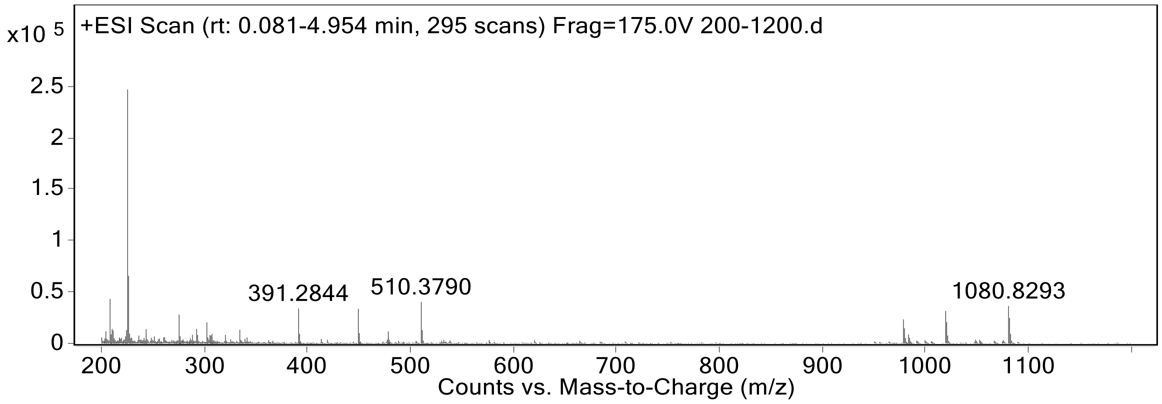

MDA-MB-231 + 2,3-DHBA / Sample 4

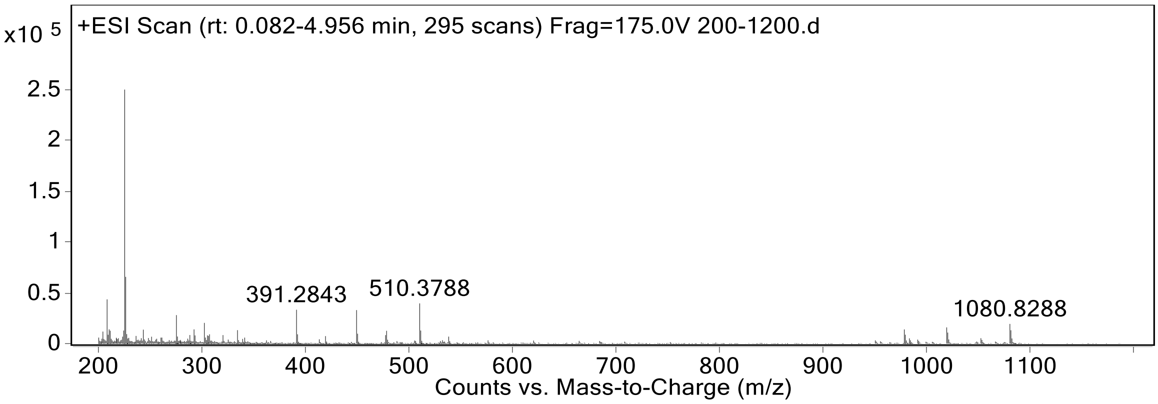

MDA-MB-231 + 2,3-DHBA / Sample 5

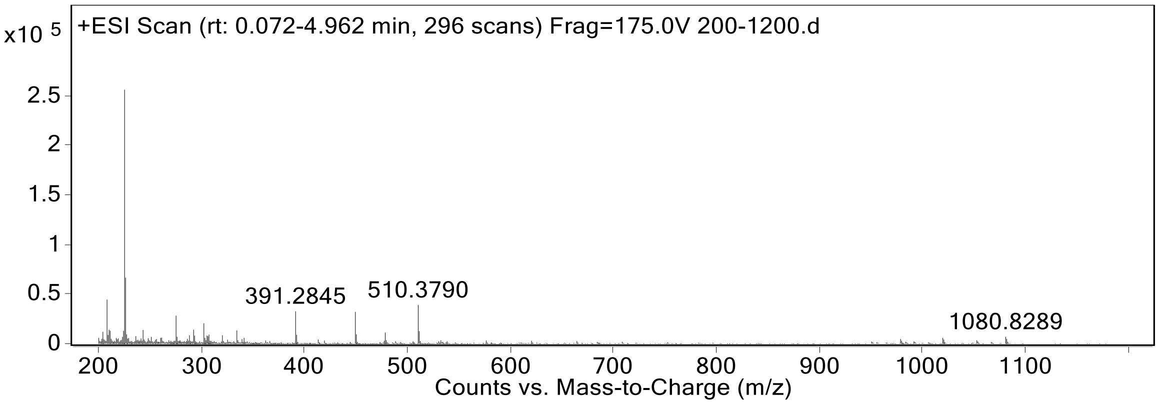

MDA-MB-231 + 2,3-DHBA / Sample 6

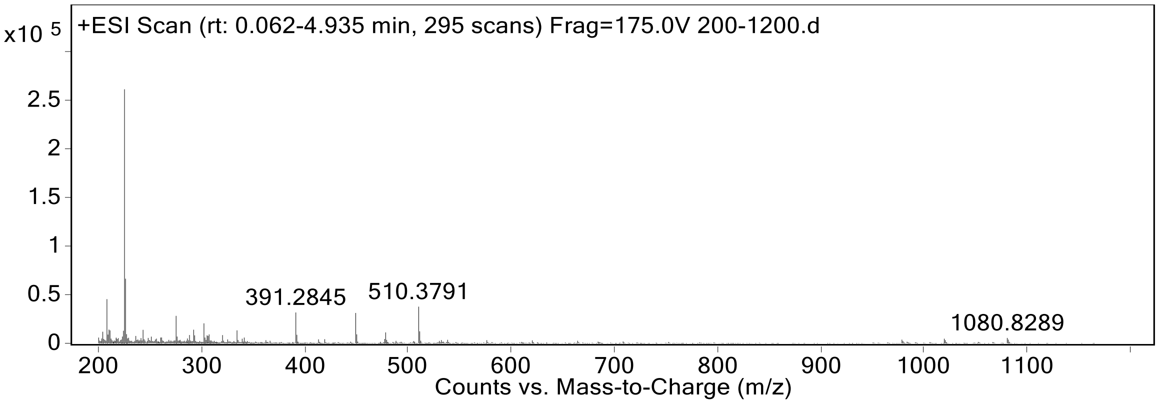

Figure S11. Positive ion ESI–MS spectra of MDA-MB-231 cells treated with DMSO (control) for 600-900 m/z

MDA-MB-231 / Sample 1

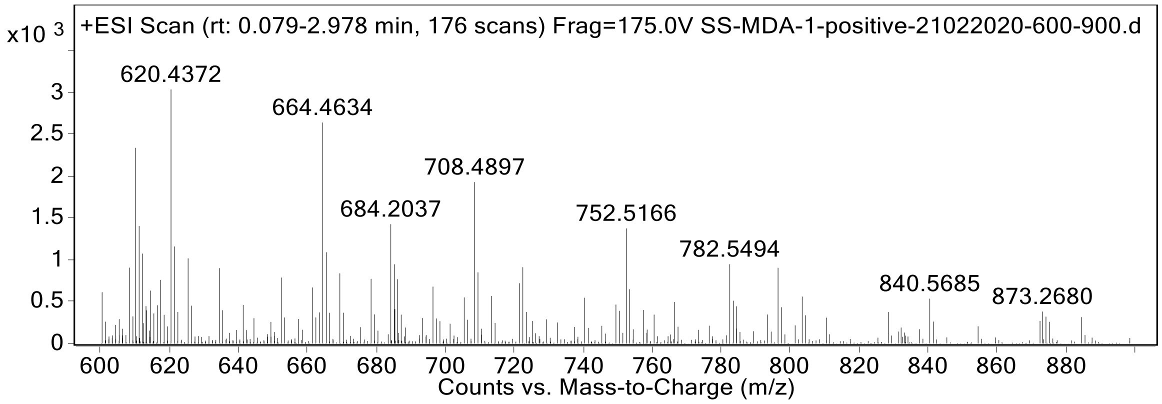

MDA-MB-231 / Sample 2

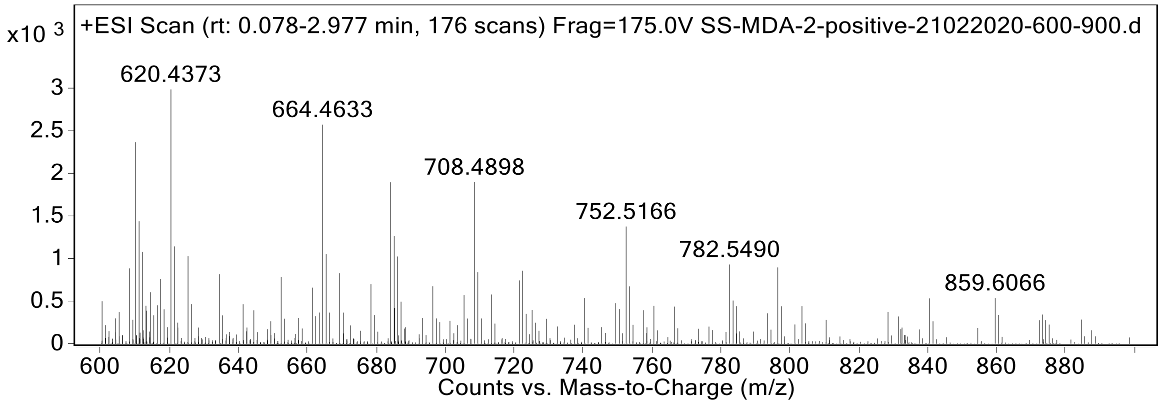

MDA-MB-231 / Sample 3

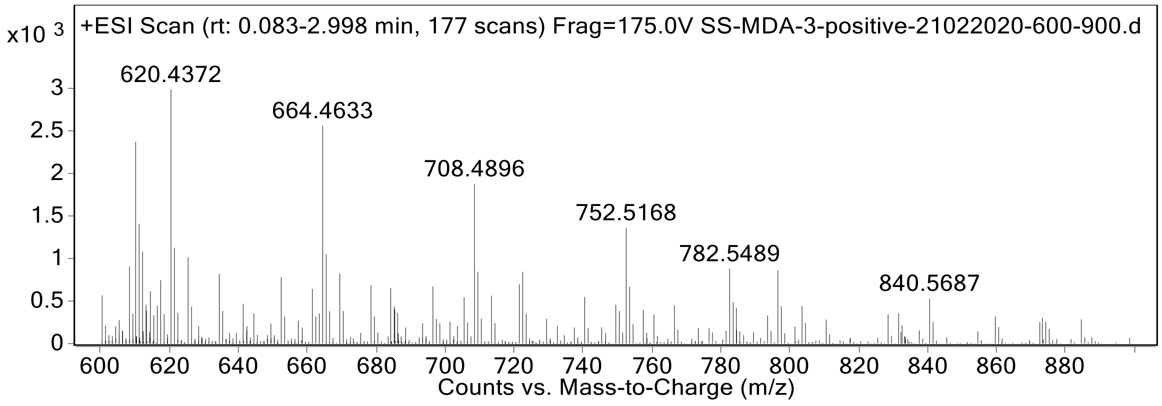

MDA-MB-231 / Sample 4

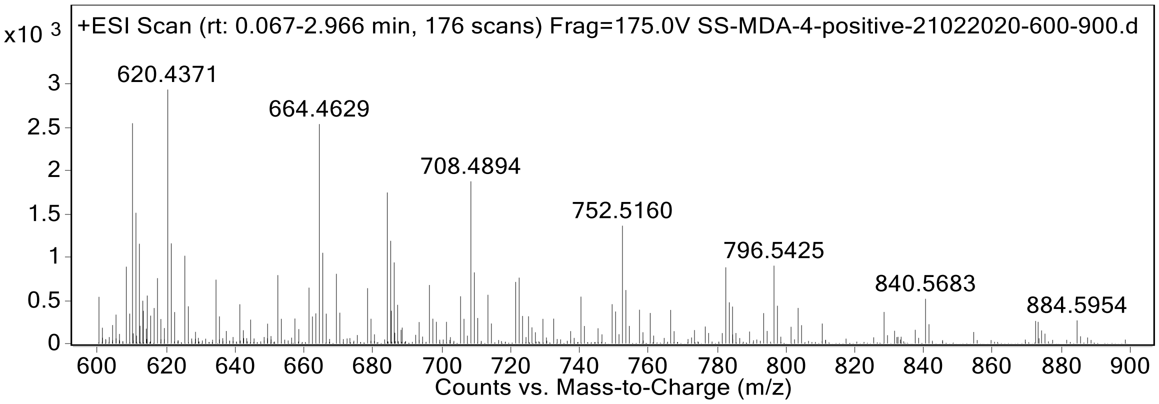

MDA-MB-231 / Sample 5

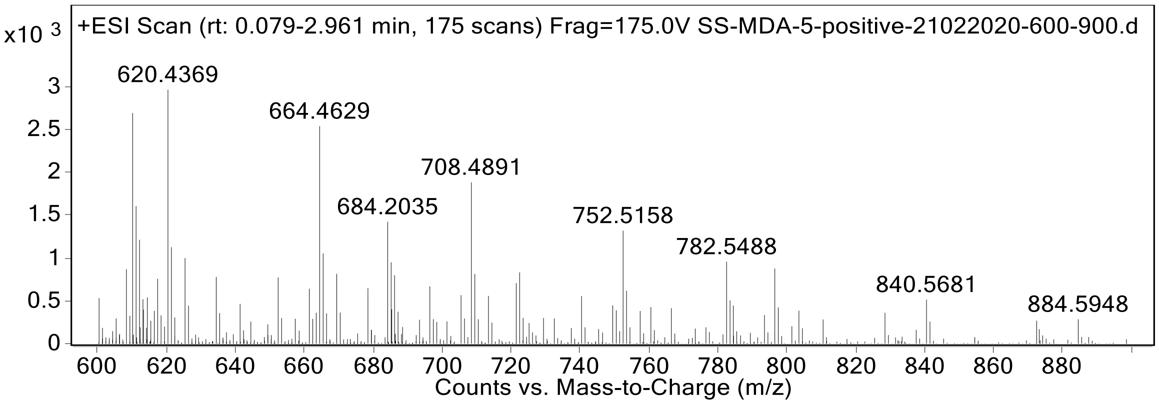

MDA-MB-231 / Sample 6

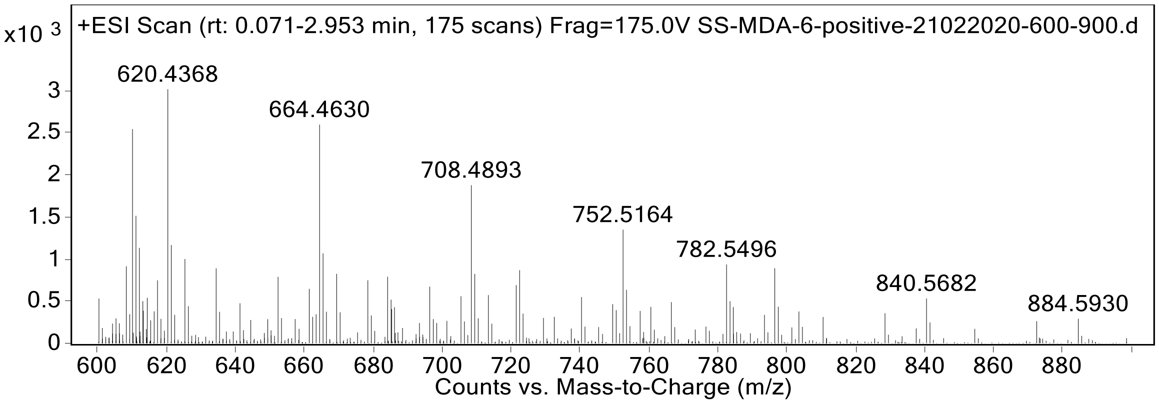

Figure S12. Positive ion ESI–MS spectra of MDA-MB-231 cells treated with IC_{50} concentration of 2,3-DHBA (8.61 mM) for 300-600 m/z.

MDA-MB-231 + 2,3-DHBA / Sample 1

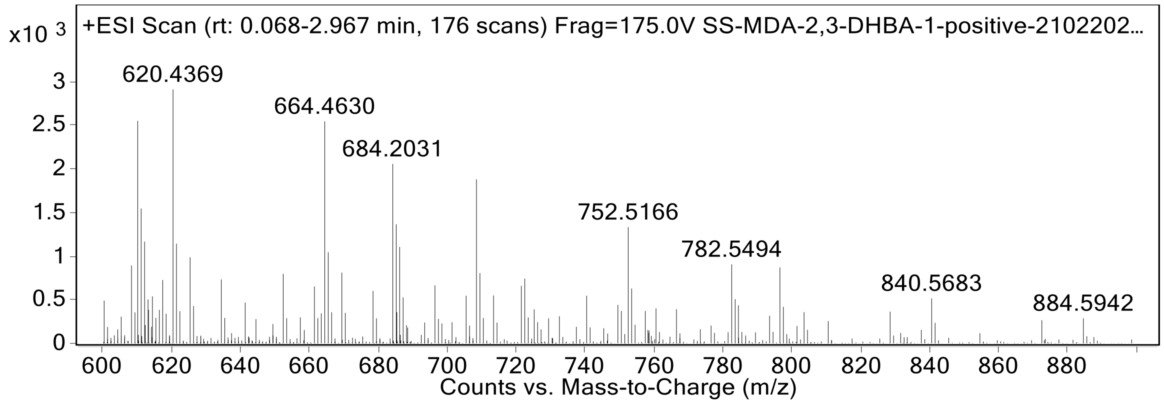

MDA-MB-231 + 2,3-DHBA / Sample 2

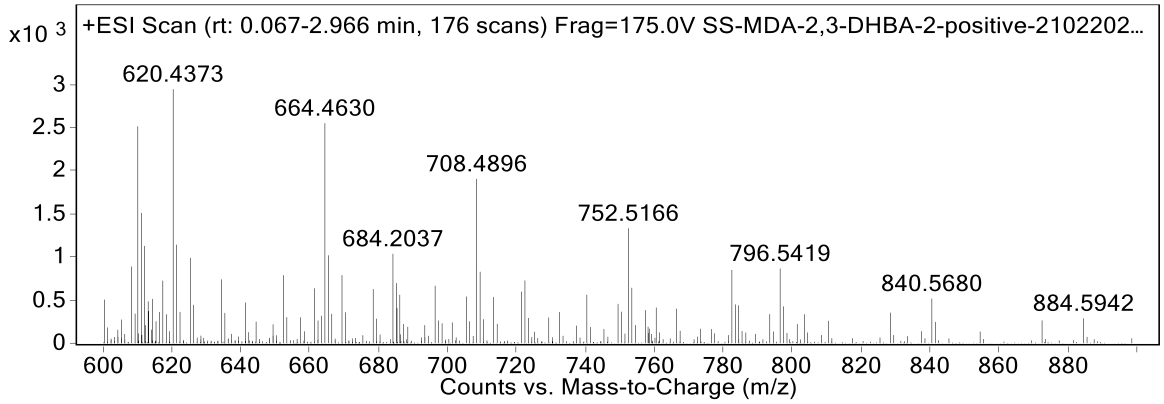

MDA-MB-231 + 2,3-DHBA / Sample 3

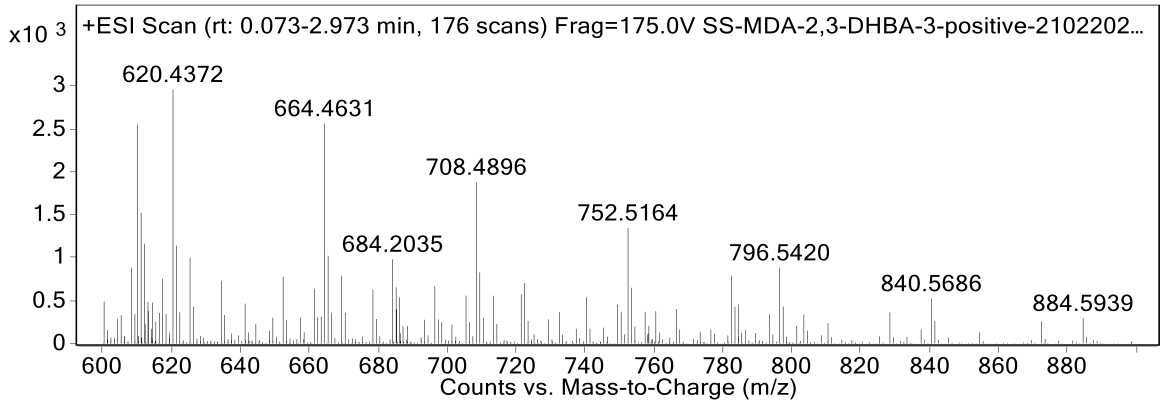

MDA-MB-231 + 2,3-DHBA / Sample 4

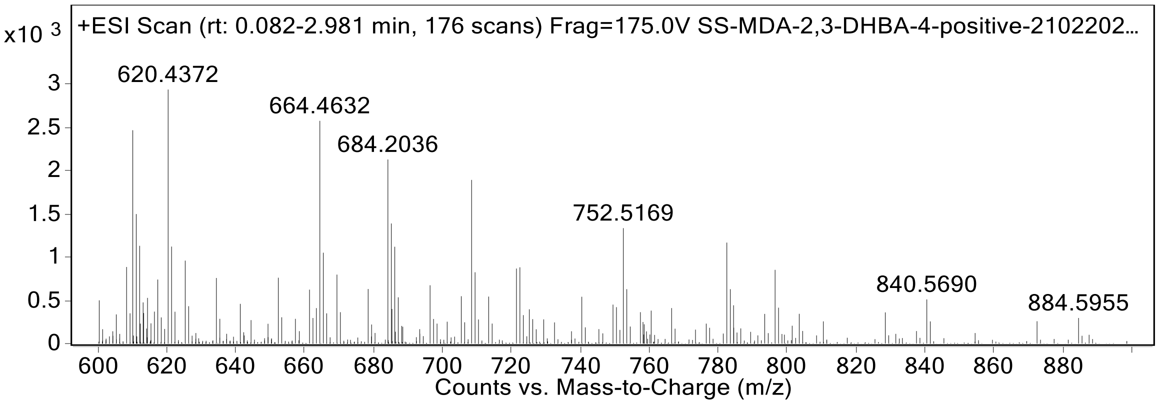

MDA-MB-231 + 2,3-DHBA / Sample 5

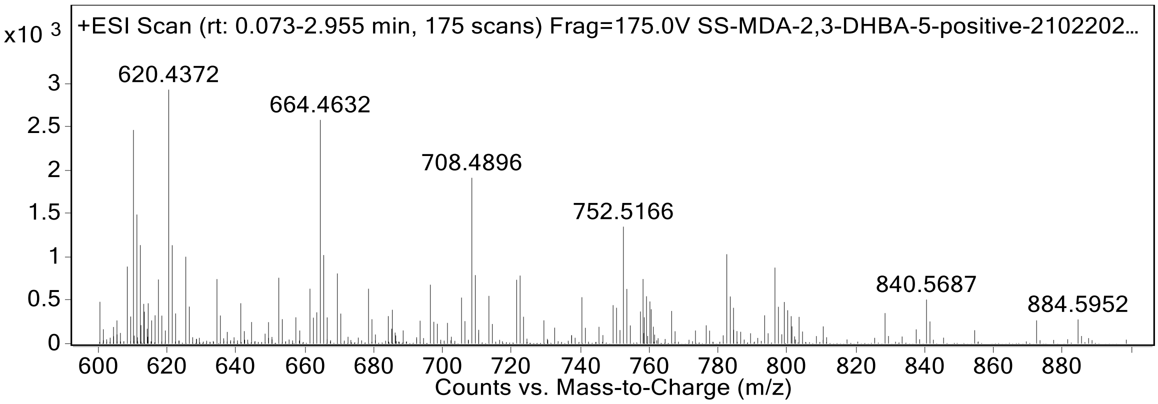

MDA-MB-231 + 2,3-DHBA / Sample 6

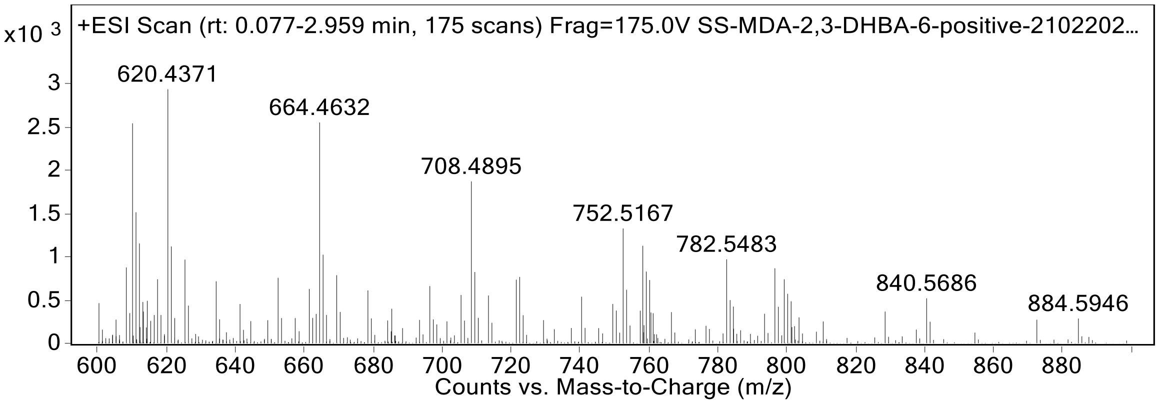

Figure S13. Negative ion ESI–MS spectra of MDA-MB-231 cells treated with DMSO (control) for 200-1200 m/z.

MDA-MB-231 / Sample 1

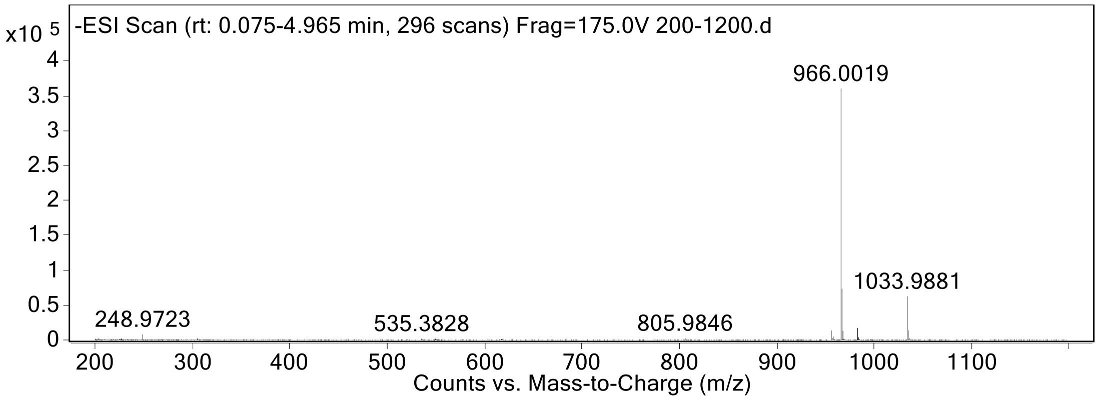

MDA-MB-231 / Sample 2

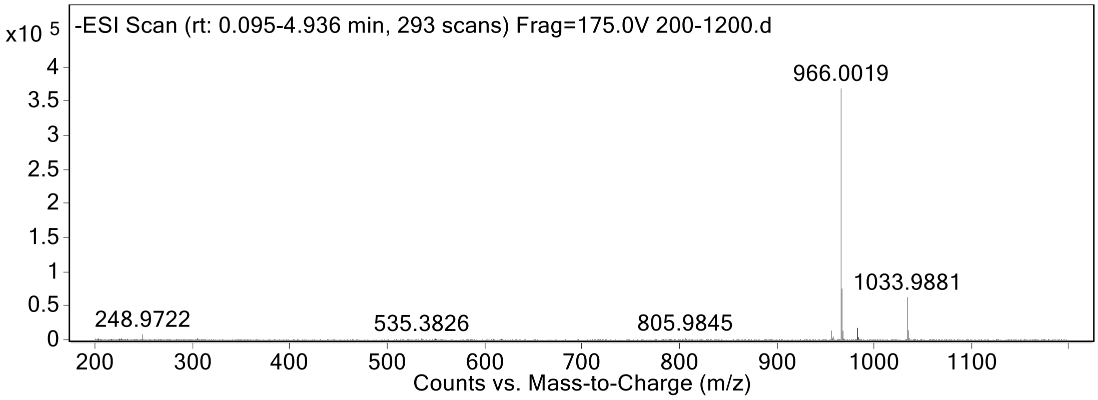

DA-MB-231 / Sample 3

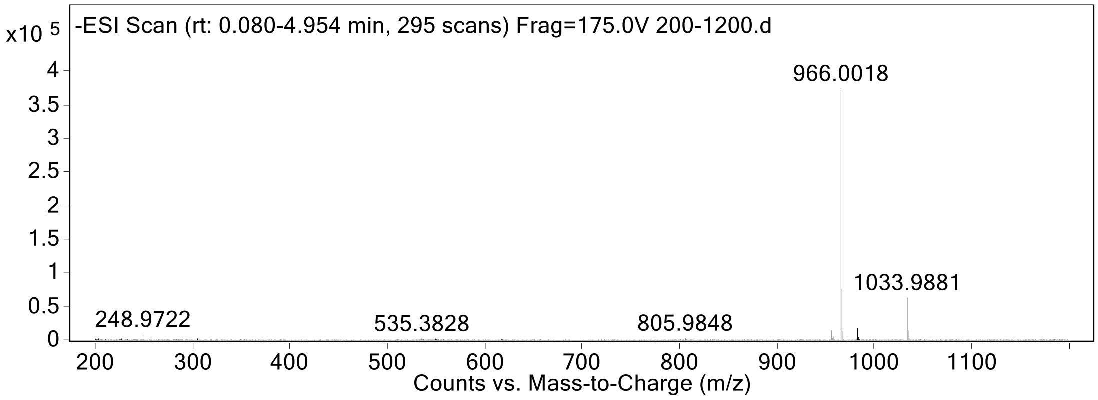

MDA-MB-231 / Sample 4

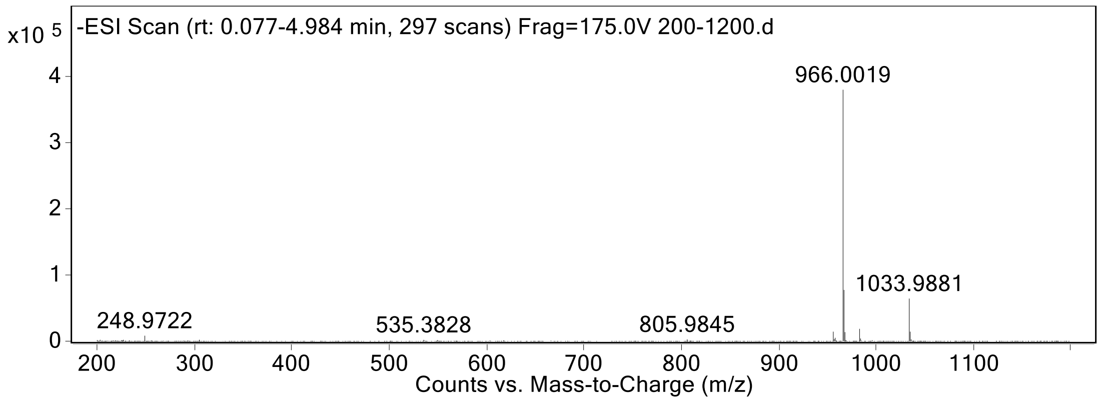

MDA-MB-231 / Sample 5

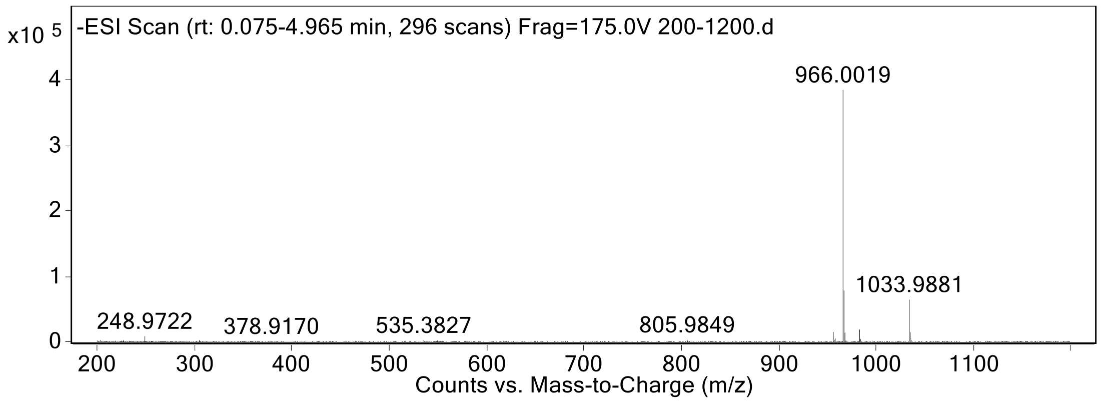

MDA-MB-231 / Sample 6

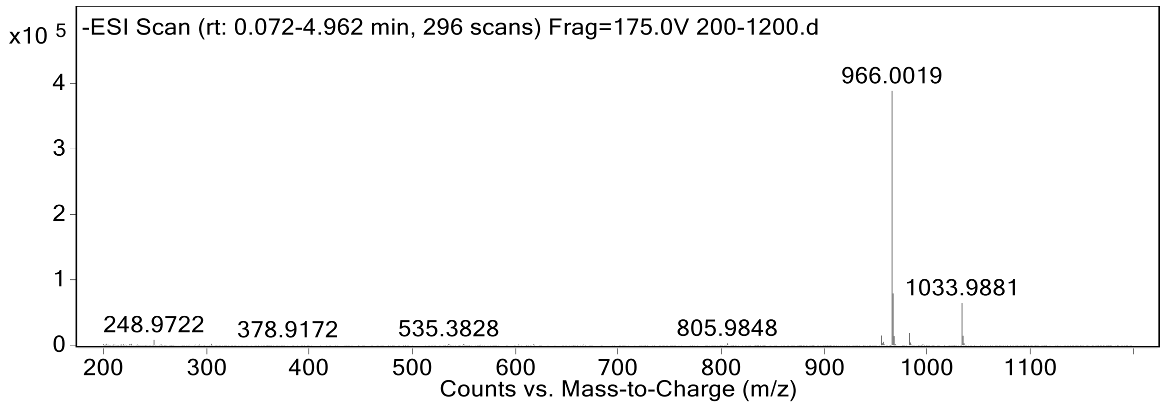

Figure S14. Negative ion ESI–MS spectra of MDA-MB-231 cells treated with IC_{50} concentration of 2,3-DHBA (8.61 mM) for 200-1200 m/z.

MDA-MB-231 + 2,3-DHBA / Sample 1

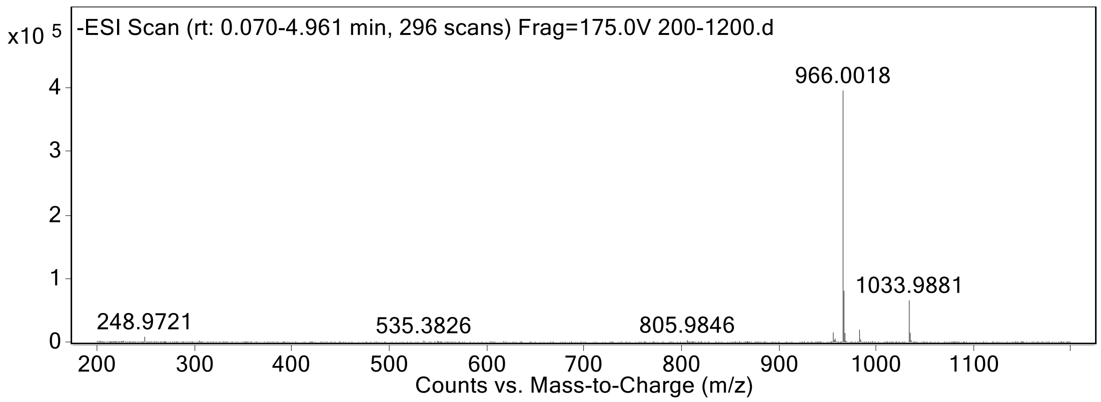

MDA-MB-231 + 2,3-DHBA / Sample 2

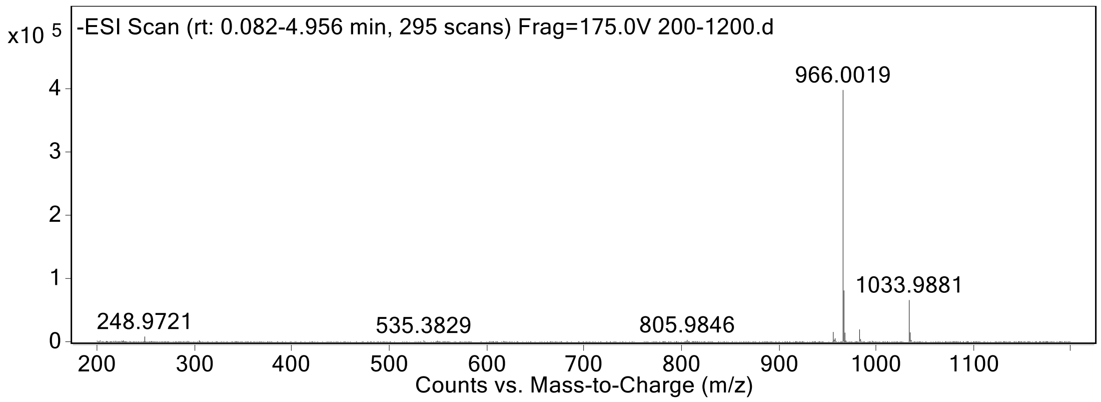

MDA-MB-231 + 2,3-DHBA / Sample 3

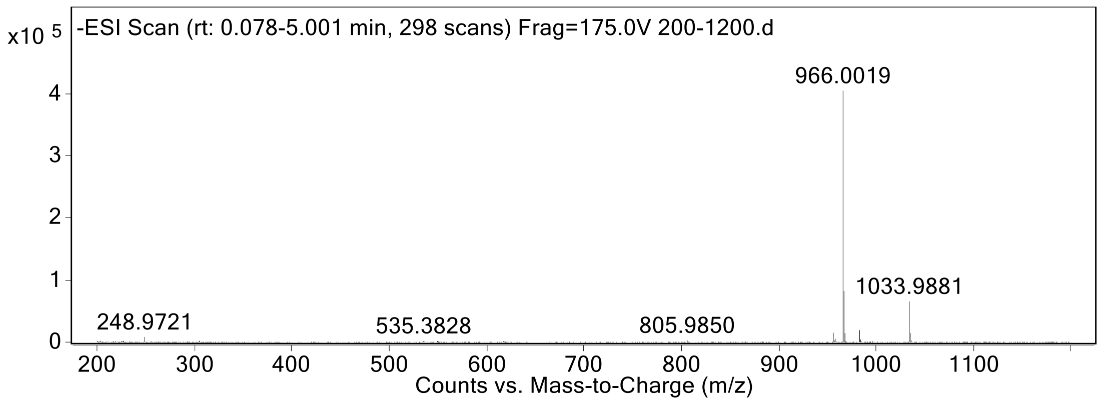

MDA-MB-231 + 2,3-DHBA / Sample 4

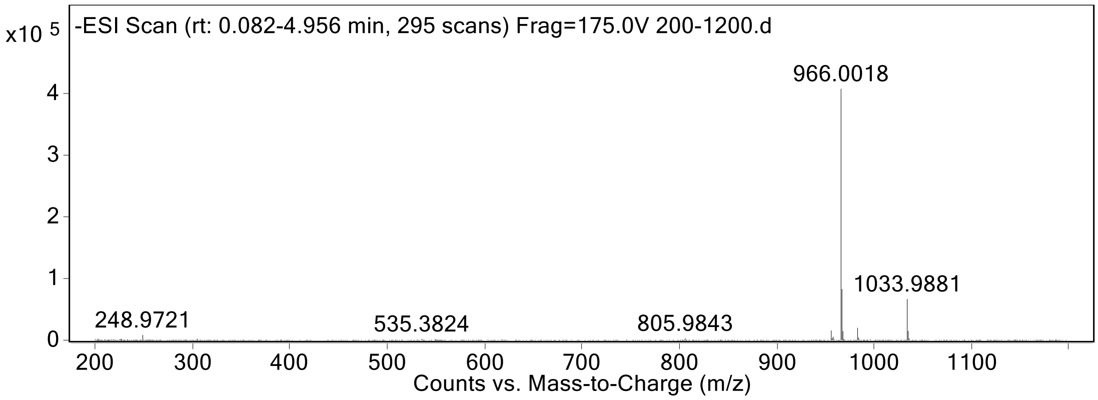

MDA-MB-231 + 2,3-DHBA / Sample 5

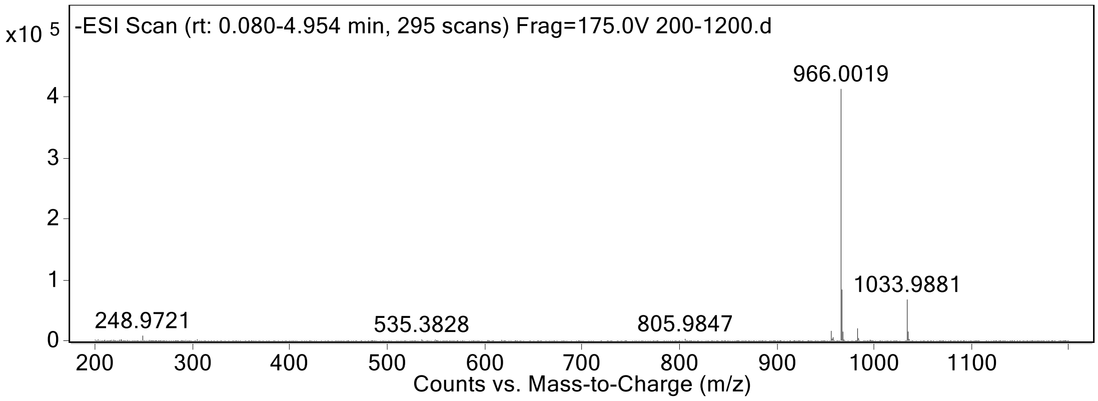

MDA-MB-231 + 2,3-DHBA / Sample 6

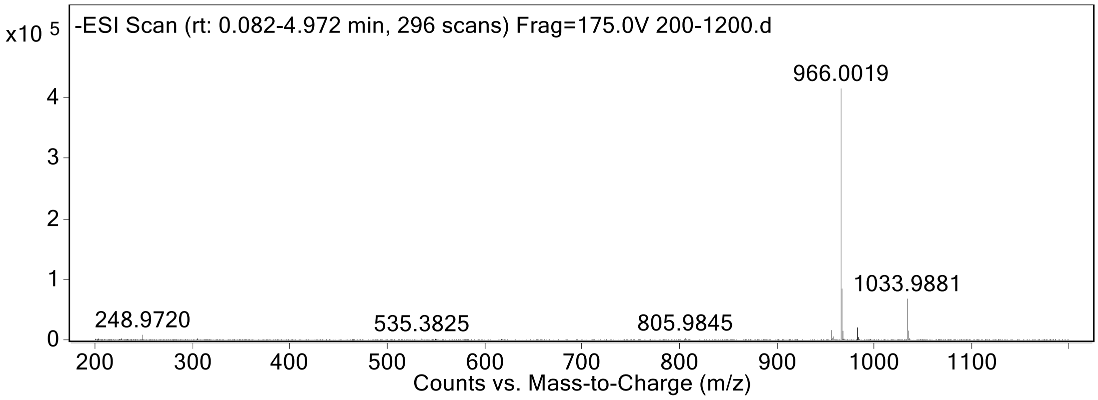

Figure 15. Negative ion ESI–MS spectra of MDA-MB-231 cells treated with DMSO (control) for 600-900 m/z

MDA-MB-231 / Sample 1

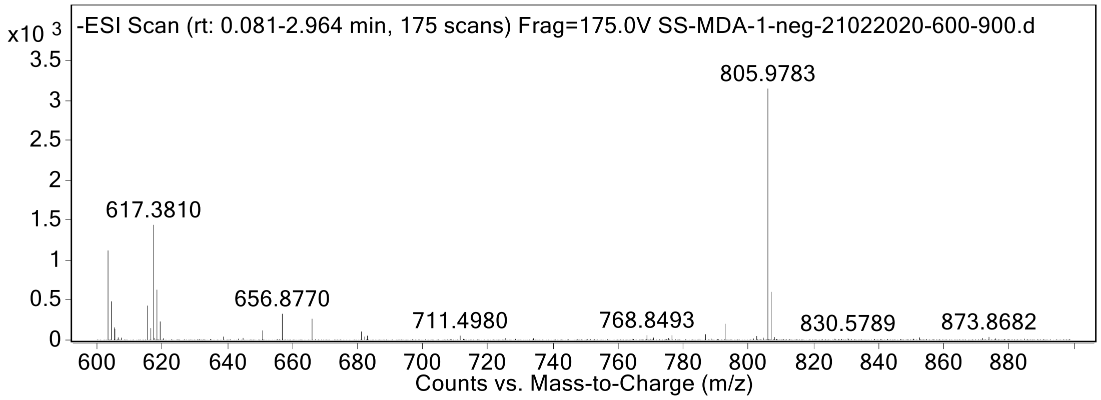

MDA-MB-231 / Sample 2

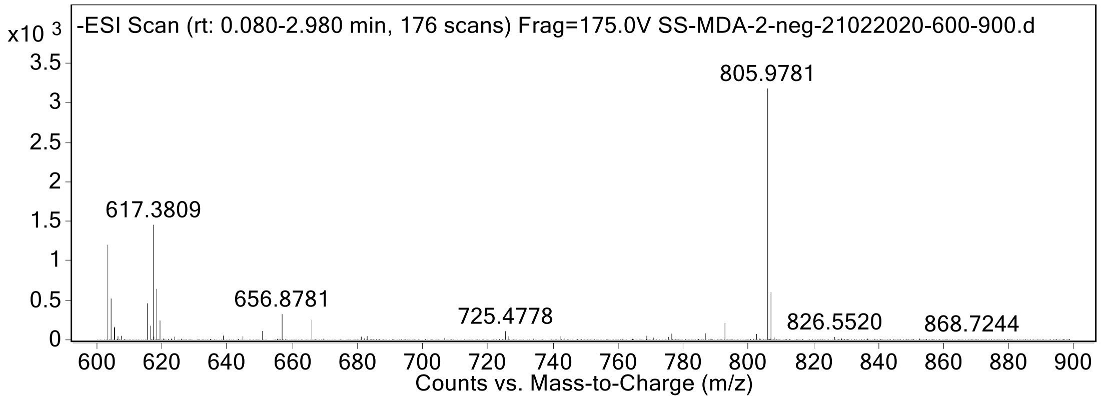

MDA-MB-231 / Sample 3

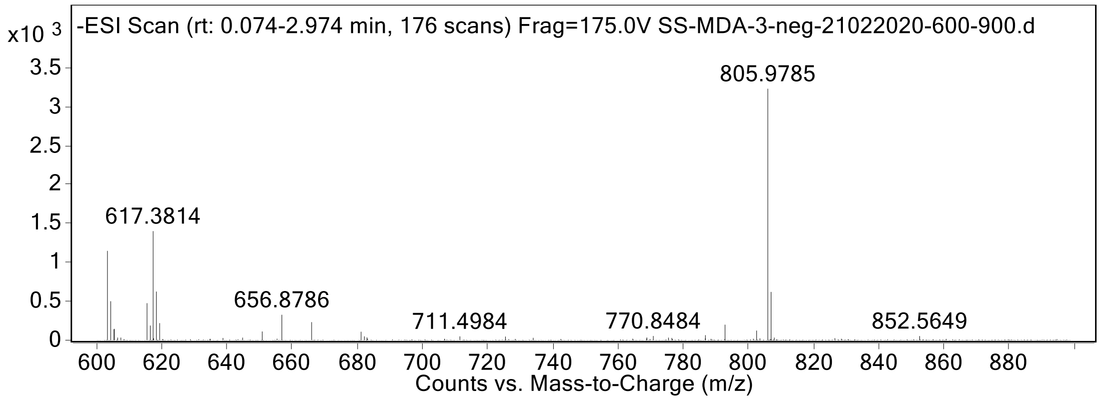

MDA-MB-231 / Sample 4

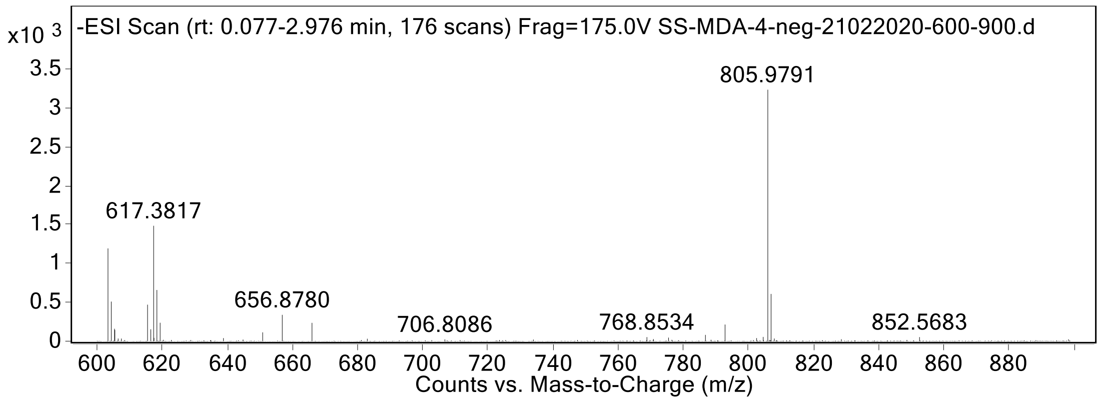

MDA-MB-231 / Sample 5

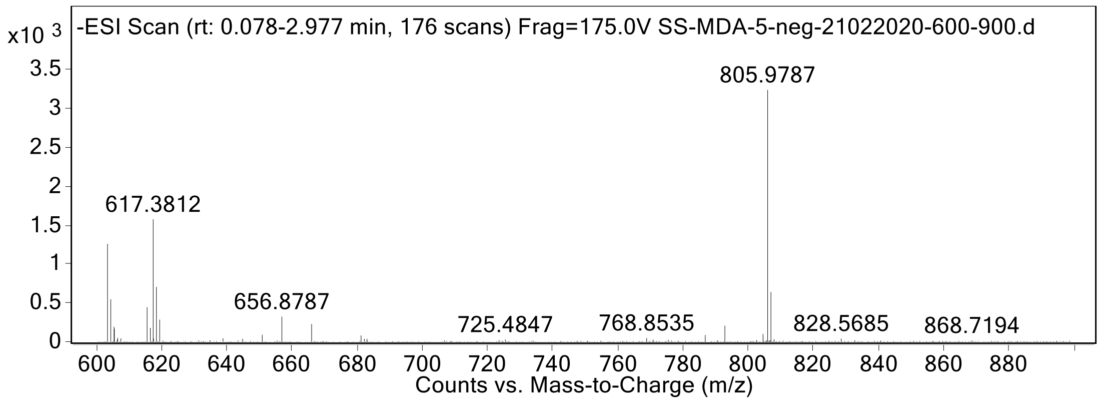

MDA-MB-231 / Sample 6

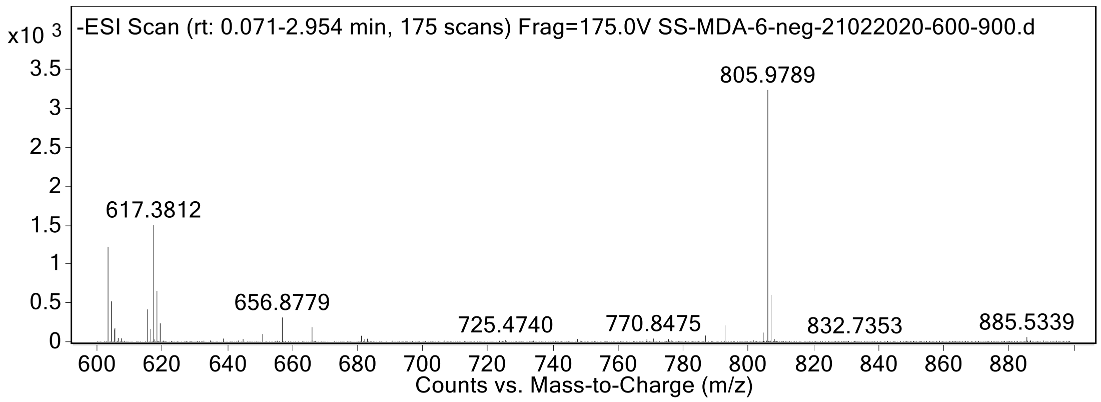

Figure S16. Negative ion ESI–MS spectra of MDA-MB-231 cells treated with IC_{50} conce ntration of 2,3-DHBA (8.61 mM) for 300-600 m/z

MDA-MB-231 + 2,3-DHBA / Sample 1

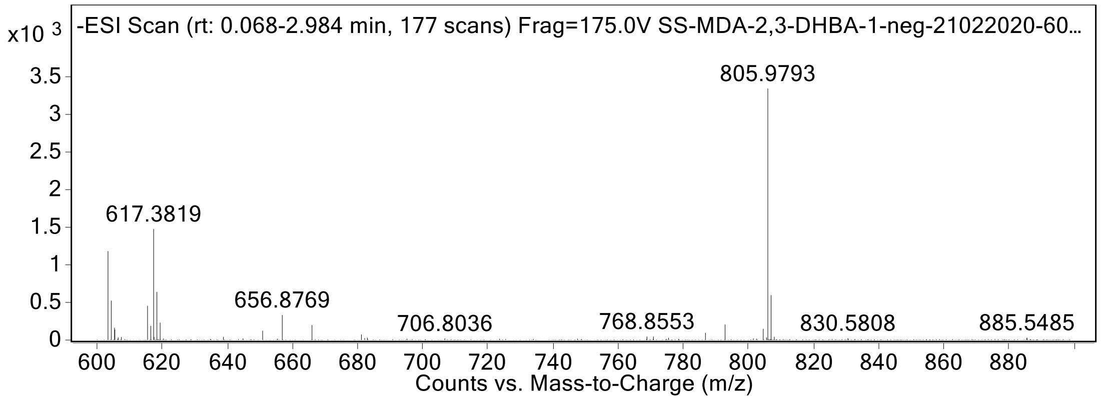

MDA-MB-231 + 2,3-DHBA / Sample 2

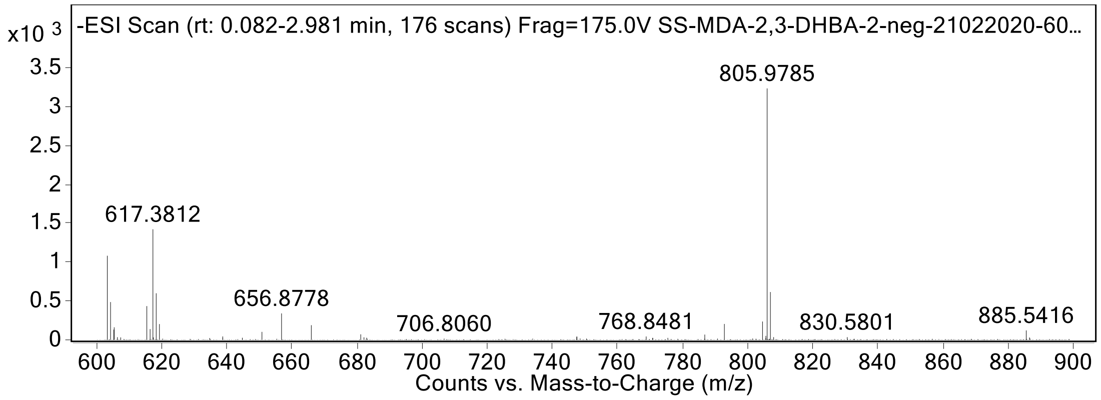

MDA-MB-231 + 2,3-DHBA / Sample 3

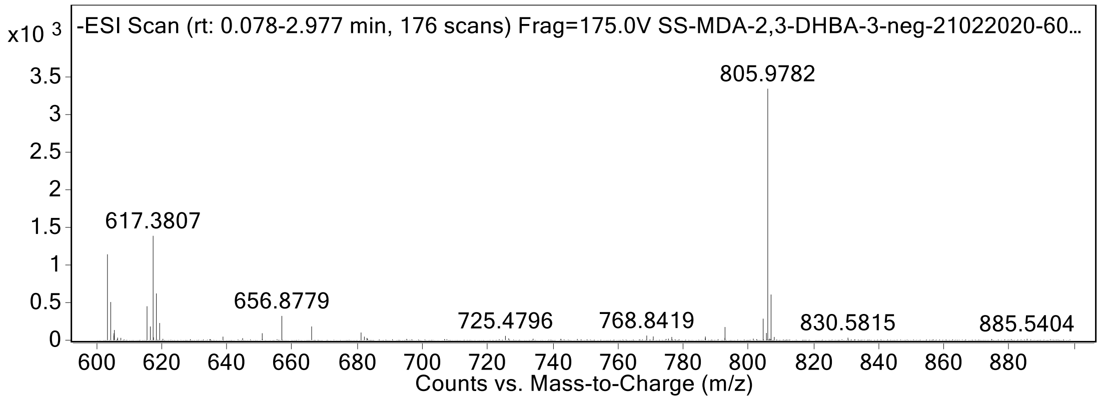

MDA-MB-231 + 2,3-DHBA / Sample 4

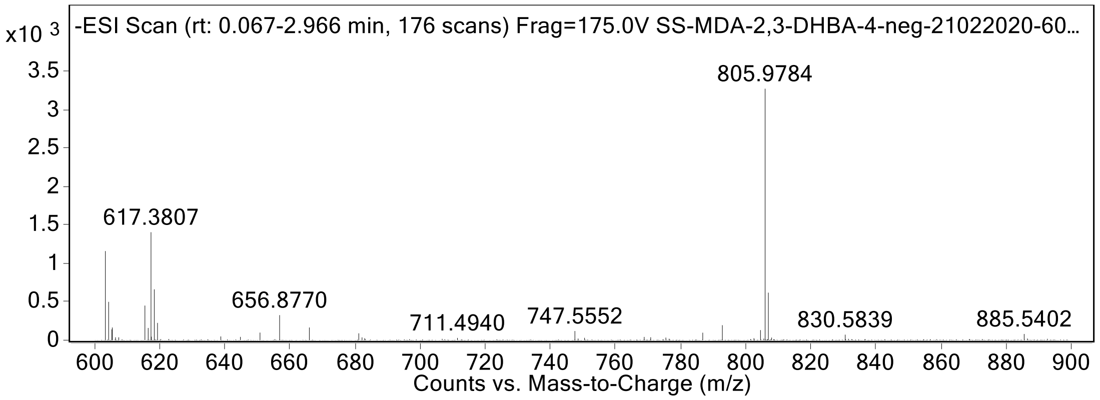

MDA-MB-231 + 2,3-DHBA / Sample 5

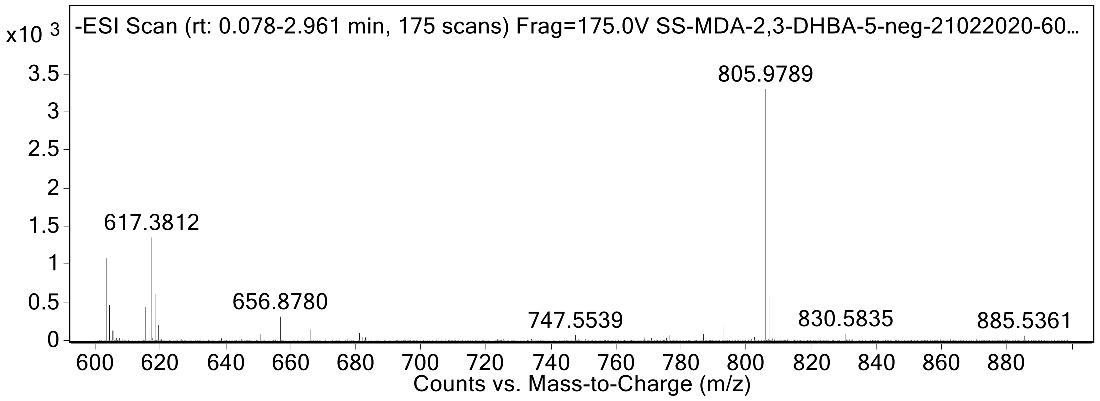

MDA-MB-231 + 2,3-DHBA / Sample 6

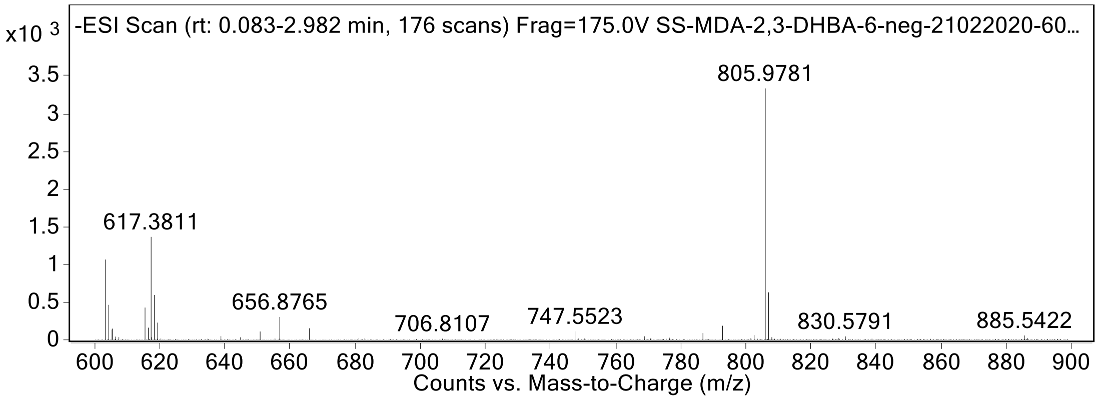

Figure S17. t-test of MCF-7 cells treated with IC_{50} concentration of 2,3-DHBA (8.61 mM) for positive ion mode

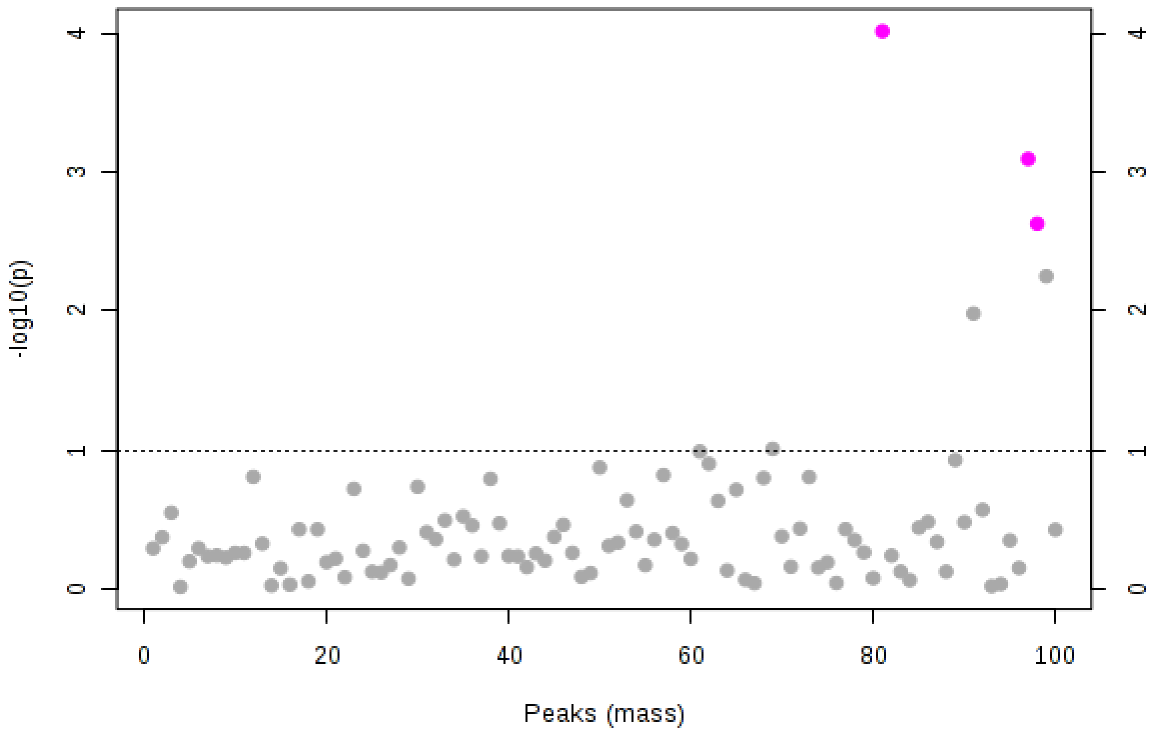

Figure S18. Fold change of MCF-7 cells treated with IC_{50} concentration of 2,3-DHBA (8.61 mM) for positive ion mode

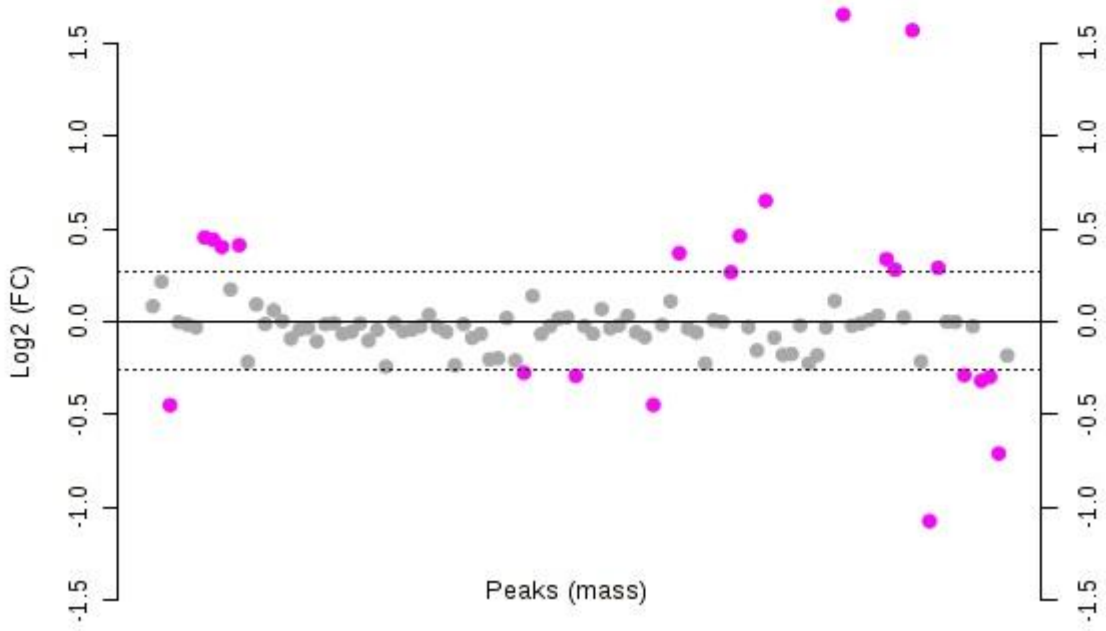

Figure S19. t-test of MCF-7 cells treated with IC_{50} concentration of 2,3-DHBA (8.61 mM) for negative ion mode

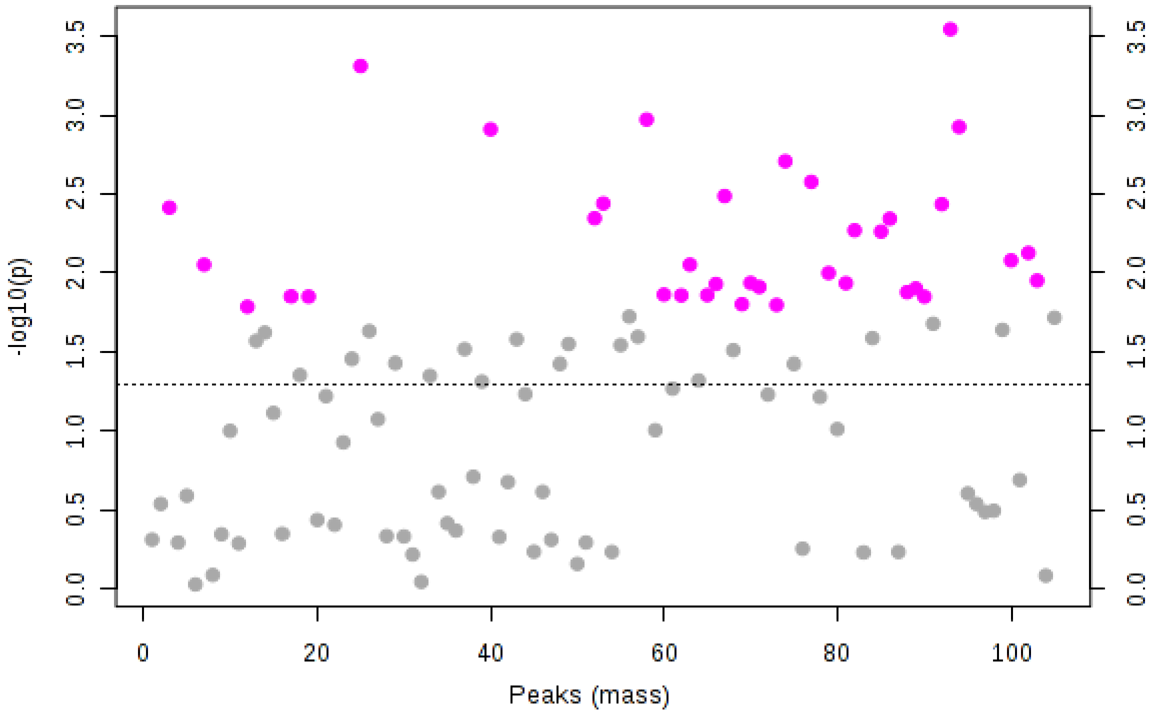

Figure S20. Fold change of MCF-7 cells treated with IC_{50} concentration of 2,3-DHBA (8.61 mM) for negative ion mode

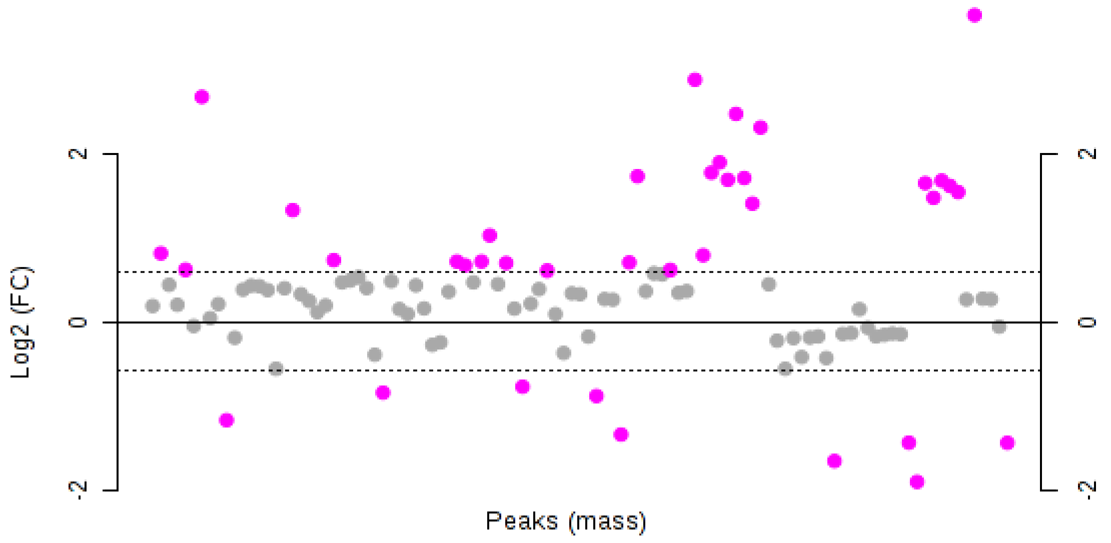

Figure S21. t-test of MDA-MB-231 cells treated with IC_{50} concentration of 2,3-DHBA (5.48 mM) for positive ion mode

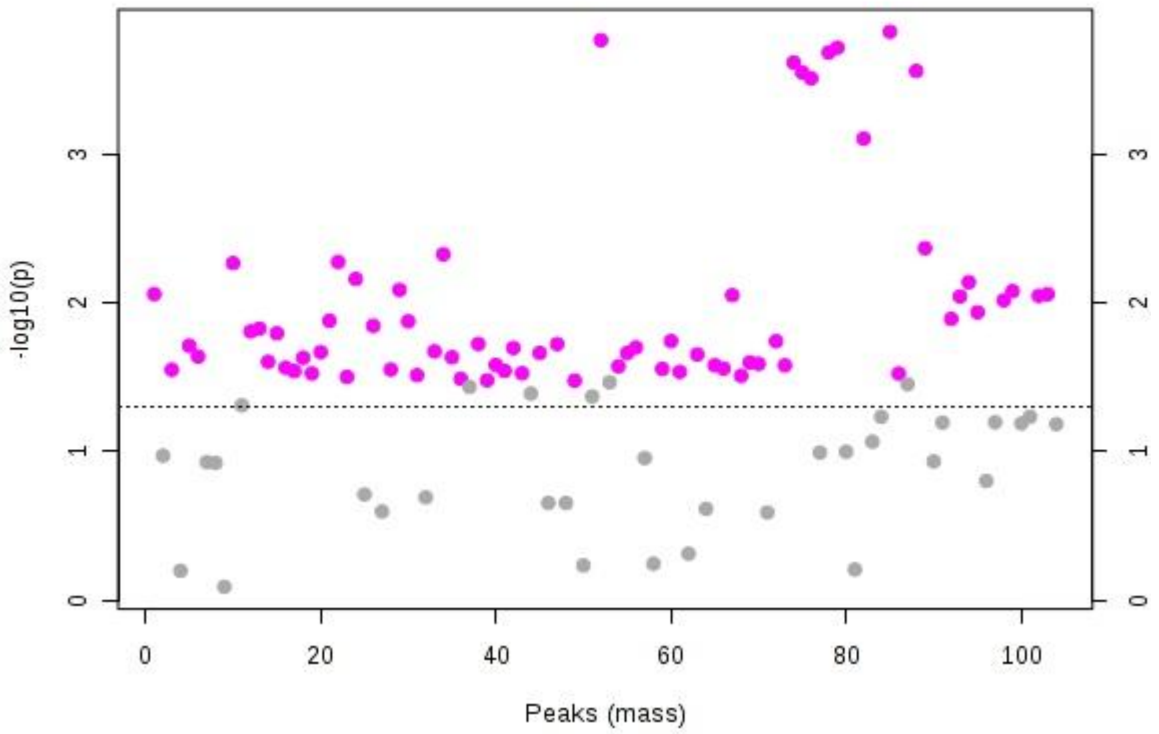

Figure S22. Fold change of MDA-MB-231 cells treated with IC_{50} concentration of 2,3-DHBA (5.48 mM) for positive ion mode

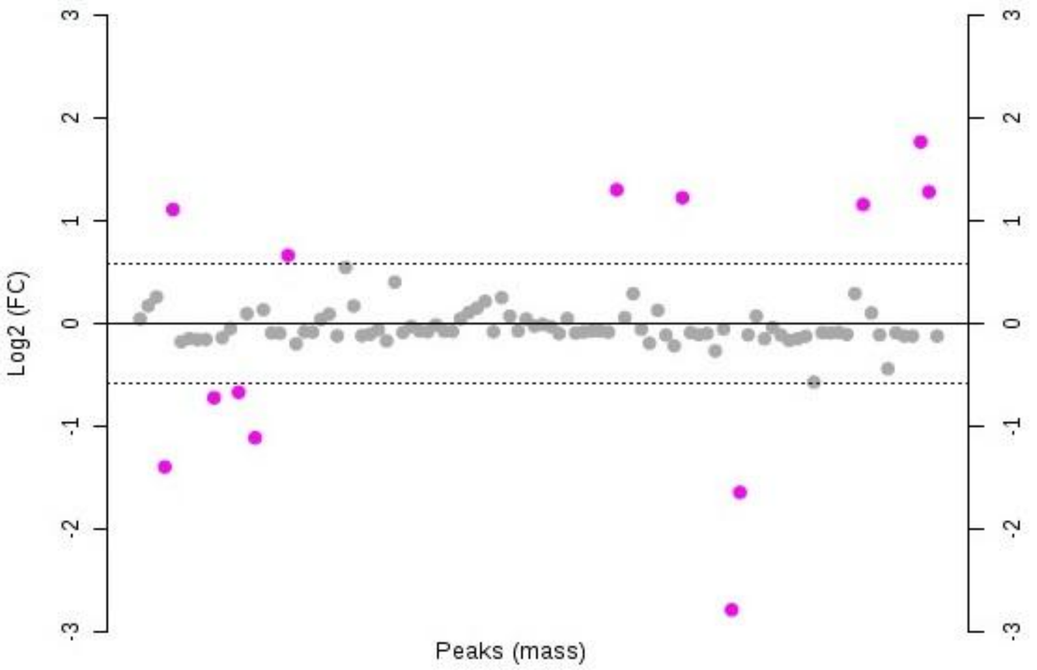

Figure S23. t-test of MDA-MB-231 cells treated with IC_{50} concentration of 2,3-DHBA (5.48 mM) for negative ion mode

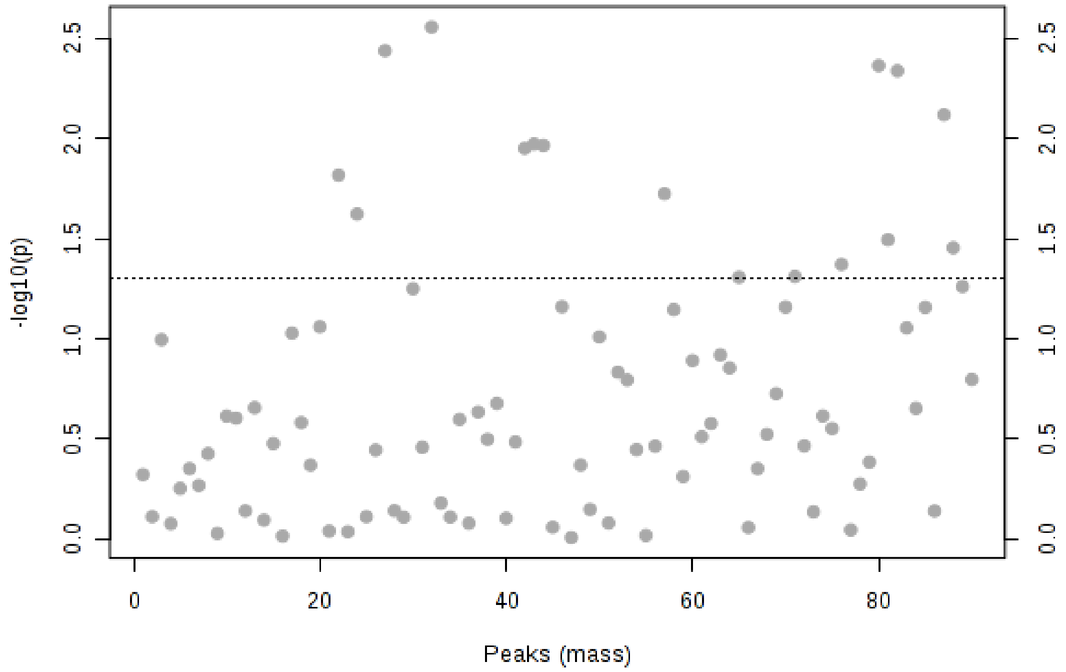

Figure S24. Fold change MDA-MB-231 cells treated with IC_{50} concentration of 2,3-DHBA (5.48 mM) for negative ion mode

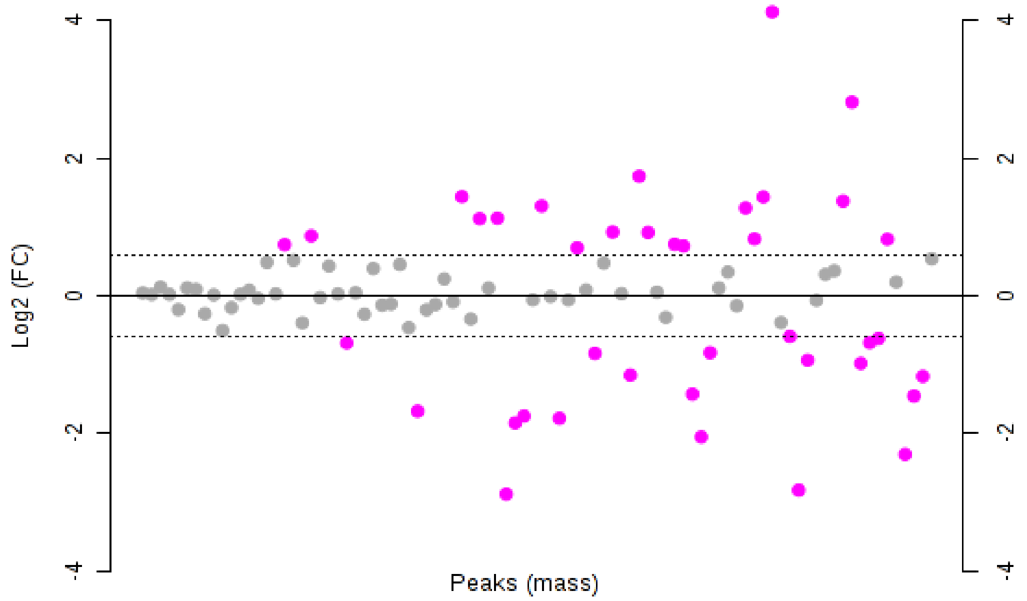

Supplement: Supplementary file 1 [file biomolecules-15-01341-s001.zip › biomolecules-3767697-supplementary.pdf]
